# The global economic burden of antibiotic-resistant infections and the potential impact of bacterial vaccines: a modelling study

**DOI:** 10.1136/bmjgh-2024-016249

**Published:** 2025-06-19

**Authors:** Nichola R Naylor, Mateusz Hasso-Agopsowicz, Chaelin Kim, Yixuan Ma, Isabel Frost, Kaja Abbas, Gisela Aguilar, Naomi Fuller, Julie V Robotham, Mark Jit

**Affiliations:** 1 Department of Health Services Research and Policy, The London School of Hygiene and Tropical Medicine, London, UK; 2 HCAI, Fungal, AMR, AMU & Sepsis Division, UK Health Security Agency, London, UK; 3 Immunization, Vaccines & Biologicals, World Health Organization, Geneva, Switzerland; 4 Department of Infectious Disease Epidemiology, London School of Hygiene & Tropical Medicine, London, UK; 5 Aquarius Population Health Ltd, London, UK; 6 School of Tropical Medicine and Global Health, Nagasaki University, Nagasaki, Japan; 7 Institute of Tropical Medicine, Nagasaki University, Nagasaki, Japan; 8 Public Health Foundation of India, New Delhi, India; 9 University of Oxford, Oxford, UK

**Keywords:** Vaccines, Global Health, Health economics

## Abstract

**Introduction:**

Antibiotic resistance (ABR) may increase hospital costs, utility loss and mortality risk per patient. Understanding these losses at national, regional and global scales is necessary for efficiently tackling ABR. Our aim is to estimate the global economic burden of antibiotic-resistant infections and the potential for bacterial vaccines to mitigate this burden.

**Methods:**

We take healthcare system and labour productivity perspectives. Hospital cost-per-case and length-of-stay estimates were calculated through meta-analyses and reviewing published systematic reviews. Unit labour productivity losses were estimated through a human capital approach. Modelled estimates were used where secondary data were missing. Death and incidence data were combined with unit cost data to estimate the economic burden associated with ABR in 2019, and the potential costs averted (in 2019 US$) based on uptake scenarios of vaccines that currently exist or are likely to be developed.

**Results:**

Multidrug-resistant tuberculosis had the highest mean hospital cost attributable to ABR per patient, the range was US$3000 in lower-income settings to US$41 000 in high-income settings, with carbapenem-resistant infections associated with a high cost-per-case of US$3000–US$7000 depending on syndrome. ABR was associated with a median value of US$693 billion (IQR: US$627 bn–US$768 bn) in hospital costs globally, with US$207 bn (IQR: US$186 bn–US$229 bn) potentially avertable by vaccines. Productivity losses were quantified at almost US$194 billion, with US$76 bn avertable by vaccines.

**Conclusions:**

The economic burden of ABR is associated with high levels of hospital bed-days occupied, hospital spending and labour productivity losses globally and should, therefore, remain high on national and international policy agendas. Vaccines against *Staphylococcus aureus, Escherichia coli and Klebsiella pneumoniae* would avert a substantial portion of the economic burden associated with ABR. More robust evidence, particularly in low-income countries, on the hospital costs, associated with and attributable to ABR, is needed.

**WHAT IS ALREADY KNOWN ON THIS TOPIC**

- Narrative literature reviews, modelling studies and global-level meta-analyses are available for cost-per-case estimates associated with antibiotic resistance (ABR); however, these have not been translated into national and regional costs.
- Vaccine impact on ABR-associated and attributable costs is only available for specific pathogens, in specific settings.

**WHAT THIS STUDY ADDS**

- Quantification of the economic burden, from hospital and labour productivity perspectives, of ABR for 14 bacterial pathogens on a global scale is provided, with a notably high burden of multidrug-resistant tuberculosis and carbapenem resistance shown from the hospital perspective.
- While total economic burden estimates differ across regions, vaccines for *Staphylococcus aureus, Escherichia coli and Klebsiella pneumoniae* could avert substantial burden across countries.

**HOW THIS STUDY MIGHT AFFECT RESEARCH, PRACTICE OR POLICY**

- Under the utilised product-profile assumptions, bacterial vaccines have the potential to avert a substantial proportion (30%–40%) of hospital and labour productivity costs associated with ABR, with this evidence aiding future research funding and investment decisions on new vaccine development.
- Future research can utilise this modelling approach with updated data when/where available for vaccine availability and coverage.

## Introduction

 Antimicrobial resistance has been estimated to cost up to US$29 289 more per patient hospital episode, while macroeconomic modelling by RAND Europe estimated the global economic costs of antimicrobial resistance at US$53 billion to US$3 trillion per year.^1 2^ A large portion of those costs is associated with antibiotic resistance (ABR) in bacterial infections.^1 2^ The economic impacts of ABR have spurred international efforts to design interventions to address this issue.^3^ However, the latter figures are based on broad macroeconomic estimates and do not provide insight into the kind of costs that are used to select interventions which are efficient from healthcare payer and/or provider perspectives. Accurate costs that are specific to disease and intervention are important to inform priority-setting around different types of interventions such as vaccine development and antibiotic stewardship. To address this gap, we must understand the cost of resistance to local healthcare systems.

Evaluations of interventions which target reducing levels of ABR (such as stewardship campaigns) require the incremental cost of an ABR infection in comparison to a susceptible infection (the attributable burden of ABR), while measures that prevent infections entirely (such as vaccination) require the incremental cost of ABR in comparison to no infections (the associated burden of ABR). From healthcare system perspectives, inpatient care is often more expensive than outpatient and community care,^4^ with a large proportion of healthcare burden associated with ABR in hospital settings.^5 6^ Additionally, a larger proportion of these costs will be associated with ABR (with a ‘no infection’ counterfactual), compared with attributable to (with a relevant ‘susceptible infection’ counterfactual).^5 6^ Literature reviews over the past decade provide a narrative synthesis and/or provide a global or regional meta-analysis of incremental impacts of healthcare system costs associated with ABR.^25^,^8^ However, it is unclear how the healthcare system impact should be utilised to estimate the economic benefit of interventions, like vaccination, if the target is a specific bacterial syndrome or within specific settings. Therefore, collating, synthesising and estimating the attributable and associated hospital costs (including associated unit costs) of ABR, broken down by specific bacteria, syndrome and region, is a key step in estimating the direct healthcare system costs.

A recent study estimated bacterial ABR-associated deaths of 4.95 million (95% uncertainty interval (UI): 3.62–6.57) for 23 pathogens and 88 pathogen–drug combinations in 2019, globally.^9^ Vaccines have the potential to play an important role in tackling ABR.^10^ A modelling study estimated that vaccines against 15 bacterial pathogens could lead to reduced drug-resistant infections, resulting in reductions of 0.51 million (95% UI 0.49–0.54) deaths in 2019. This reduction in mortality combined with associated reductions in morbidity implies that vaccines could potentially avert 28 (95% UI: 27–29) million associated disability-adjusted life-years lost, over the same period.^11^ Focusing on low-income and middle-income countries, paediatric vaccines were estimated to potentially avert 181 500 (153 400–206 800) antimicrobial resistance-associated deaths, from both the direct prevention of antimicrobial resistant infections and also via reductions in antibiotic consumption.^12^ The scale of potential impact varies not only depending on setting, but also on target pathogen, with extraintestinal pathogenic *Escherichia coli* vaccines potentially averting 130 016 antimicrobial resistance-associated bloodstream infection (BSI) deaths and a *Shigella* spp vaccine averting 4133 deaths, globally.^10^ A reduction in drug-resistant infections due to vaccines likely not only reduces ABR-associated morbidity and mortality impacts,^13^ but also hospital costs and lost labour productivity.^1^

There is evidence of pneumococcal vaccination averting ABR-related costs^14 15^ and reductions in antibiotic use,^16 17^ and a few modelling studies have evaluated the potential impact of vaccines on antibiotic use.^12 16^ However, the reduction of ABR-associated direct hospital costs and labour productivity losses avertible by vaccination is largely unknown across other priority, bacterial pathogens. Understanding the potential healthcare system cost and labour productivity losses due to such pathogens, and the fraction avertible by vaccination, is key for policy surrounding future research funding and intervention investment decisions, including new vaccine development.^18^

We, therefore, aimed to estimate region-specific and pathogen-specific (1) hospitalisations attributable to, and associated with, ABR, (2) labour productivity losses per excess death, (3) antibiotic unit costs and (4) total inpatient hospital and labour productivity costs associated with ABR in 2019. We also estimated vaccine impact on reducing hospital and labour productivity costs through reduced antibiotic-resistant infections.

## Methods

We conducted this study by following the Consolidated Health Economic Evaluation Reporting Standards recommendations (see online supplemental material S1).^19^ We focused on key bacteria in relation to ABR for which there is known literature on burden and vaccine impact,^11^ see online supplemental material S2A for the list of included exposure groups (defined by pathogen, syndrome and antibiotic class of interest). We used various forms of data retrieval, collation and synthesis from published datasets and the literature, combining this with economic approaches to translate these data into costs. All hospital and antibiotic costs were estimated from the healthcare payer perspective. We developed a unit cost repository for (1)–(3), which is available online,^20^ while (4)–(5) are based on combining these unit costs with health impact estimates of vaccines on antimicrobial resistance from a prior study, with code available online also.^11 21^

Outcome measures (1)–(5) are estimated at the WHO region level, by averaging cost estimates for all countries in that region, weighted by 2019 population values.^22^ All monetary values are reported in 2019 US dollars (US$). To convert cost estimates of different currencies and of different cost years from the literature into 2019 US$, the World Bank (WB) data for Purchasing Power Parity exchange rates and local currency unit exchange rates was used, and IBAN data were used for local currency code matched to country,^23 24^ see online supplemental material S2B for more inflation process information.

### Hospital cost-per-case

To estimate the hospital cost-per-case associated with ABR for the selected bacteria, we conducted a rapid review of previously published systematic reviews due to multiple systematic reviews being published previously within this area (see online supplemental material S2C for search terms, inclusion criteria and data extraction). This focused on penicillin resistance and glycopeptide resistance in Gram-positive bacteria, third-generation cephalosporin (3gc) resistance and carbapenem resistance in Gram-negative bacteria and multidrug resistance (MDR) in tuberculosis (TB), as these combinations have been previously highlighted as key exposure groups for ABR and the cost of ABR.^7 25^

We classified attributable (resistant vs susceptible) and associated (resistant vs no infection) length of hospital stay (LoS) and hospital-cost impact for the exposure groups of interest into broader categories based on predefined hierarchies of evidence. These were based on antibiotic class, Gram-stain and regional groupings. We extracted LoS and cost impacts (see online supplemental material S2B), and converted excess LoS estimates into excess costs by using WHO-CHOICE bed day costs (at the country level) (see online supplemental material S2D).^26^

There were not enough data to inform estimates for every country. Therefore, to use the most relevant data, we grouped countries into regions. If more than one study was available for a particular exposure group-region combination, then outcomes were pooled by conducting random-effects meta-analysis and weighted using the inverse of their variance.^27^ We assigned data to countries based on a regional hierarchy. We assessed the strength of evidence for each outcome by weighting the number of studies used in the meta-analysis by proximity to country of interest (WHO-CHOICE, WB Income, WB Regional and Global were weighted 4, 3, 2 and 1, respectively). Hierarchies were confirmed based on visually inspecting the spread of hospital cost data by group.

Once pooled exposure impacts were estimated through meta-analyses, we generated 1000 random samples from the uncertainty distribution of the pooled estimate (assuming normal distribution). We combined LoS samples with 1000 random samples drawn from the distribution of hospital bed-day costs (assuming log-normal distributions) to estimate the mean and 95% UIs, based on the 2.5th and 97.5th quintiles.

### Antibiotic unit costs

We estimated antibiotic unit costs using supplier median cost data from the 2015 International Medical Products Price Guide (average catalogue prices available from non-profit suppliers, a low-cost estimate) and matched to the 2019 Access, Watch, and Reserve (AWaRe) classification for antibiotics.^28 29^ We adjusted this base price using two previous studies estimating the generic cost of oral and injectable drugs from global perspectives ^30 31^ (see online supplemental material S3A).

### Labour productivity unit costs

We estimated labour productivity losses based on employment-adjusted wage approaches using the human-capital method.^32^ We extracted mean nominal monthly earnings of employees and employment-to-population ratios (from 1990 to 2019) and aggregated over sex and age from the International Labour Organization.^33^ We calculated monthly earnings, adjusted by employment ratios and used as proxies of productivity costs per working month lost. We considered mean annual growth rates for both wage and employment-ratio estimates (see online supplemental material S3B)).

### Scenario sensitivity analyses

We conducted additional scenario sensitivity analyses to test the impact of methodological assumptions. For hospital cost-per-case, an additional ‘scenario 2’ multiplied LoS-based costs by an adjustment factor. We calculated (by income group) the ratio between costs based on multiplying patient LoS by unit costs of bed days and directly estimated costs per hospital episode (see online supplemental material S4). For antibiotic costs, we compared price-guide costs to the unit prices for antibiotics present in both lists for the UK (a proxy for high-income countries), South Africa (a proxy for upper-middle-income countries) and India (a proxy for both lower-middle-income countries (LMICs) and low-income country (LICs), since no LICs data were available) for ‘scenario 2’.^28 30 31^ For labour productivity costs in scenario 2, we used available employment-adjusted wage estimates and inflated to 2019 US$ using the Gross Domesti Product (GDP) deflation functionality, rather than using the trends calculated for wage and employment growth based on previous years’ data.

### Economic burden of drug-resistant infections and potential vaccine impact

To estimate the economic burden of ABR and vaccine impact on hospital costs, hospital bed-days, labour productivity costs and working-life-years-lost (WLYL), we combined unit cost data with epidemiological impact. Point estimates of case numbers and deaths from a previous epidemiology model that estimated the health impact of bacterial vaccines on ABR-associated cases and mortality in 2019 were used (providing efficacy, coverage, duration of protection, target group and type of disease prevented assumptions aligned with previously ustilised WHO assumptions, see online supplemental material S5A).^4^ ^11^ These included the drug-pathogen-syndrome combinations listed in online supplemental material S2B, focusing on ABR-associated burden (where no infection is the counterfactual). If multiple vaccine scenarios for a pathogen were available, the one with the highest potential impact was selected.^9 11^

Unit costs for *Salmonella*, *Shigella* or ABR associated with gastro-related illnesses, Group A *Streptococcus* (GAS) and *Haemophilus influenzae* type b (Hib) were not estimated within the unit cost repository, due to lack of published data in the previous systematic reviews. Therefore, we used results from a systematic review on the impact of ABR on *Salmonella* in hospitals^34^ and results of an expert elicitation exercise conducted by the WHO for the LoS impacts of ABR bacteria linked to gastro-related illness, GAS and Hib (see online supplemental material S5B).

When combining economic and epidemiology outcomes, we sampled LoS estimates and combined them with WHO-CHOICE unit bed-day costs (see online supplemental material S5C for more detail on sampling and missing data methods). To estimate the impact of vaccines on hospital costs, we estimated the proportion of cases which were treated in hospitals from region-syndrome-specific data in previous global ABR analyses,^9^ supplemented by the WHO expert elicitation exercise for gastro-related illness hospitalisation proportions (see online supplemental material S5D). We combined them with the unit hospital costs associated with ABR, at the country level. Antibiotic costs were not used in the estimation of total annual burden as data on antibiotics used per case for each exposure group of interest was not available.

We calculated labour productivity losses, across the lifetime horizon, by combining WHO regional deaths by age group with WHO regional life expectancy (to estimate WLYL) and unit costs^35^ (see online supplemental material S5E).

**Figure 1:**
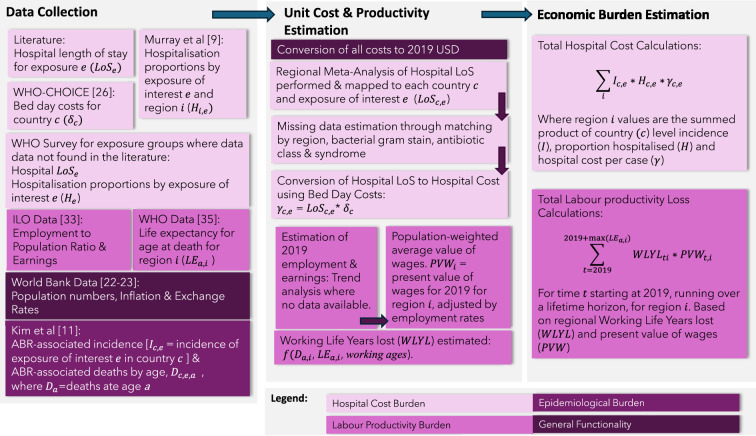
Model processes for estimating the economic burden of drug-resistant infections. ABR, antibiotic resistance; ILO, International Labour Organization; LoS, length of hospital stay.

We used the time horizon of 1 year to estimate healthcare system burden. To convert mortality impacts into labour productivity losses, we calculated the total lifetime productivity losses of the deaths that occurred in 1 year (2019). A discount rate of 3% was used for productivity loss estimation.^25^ Costs are in 2019 US$. We also estimated bed-days and potential WLYL (undiscounted and not considering employment rates) to provide maximum capacity estimates of impact. We estimated median and IQRs of the healthcare system burden. We calculated point estimates for labour productivity as only point estimates in both the unit costs and incidence estimates were available at the time of analysis. An overview of the core model processes is given in figure 1.

### Patient and public involvement

Patients and the public were not involved in this research study.

## Results

### Unit costs

**Figure 2:**
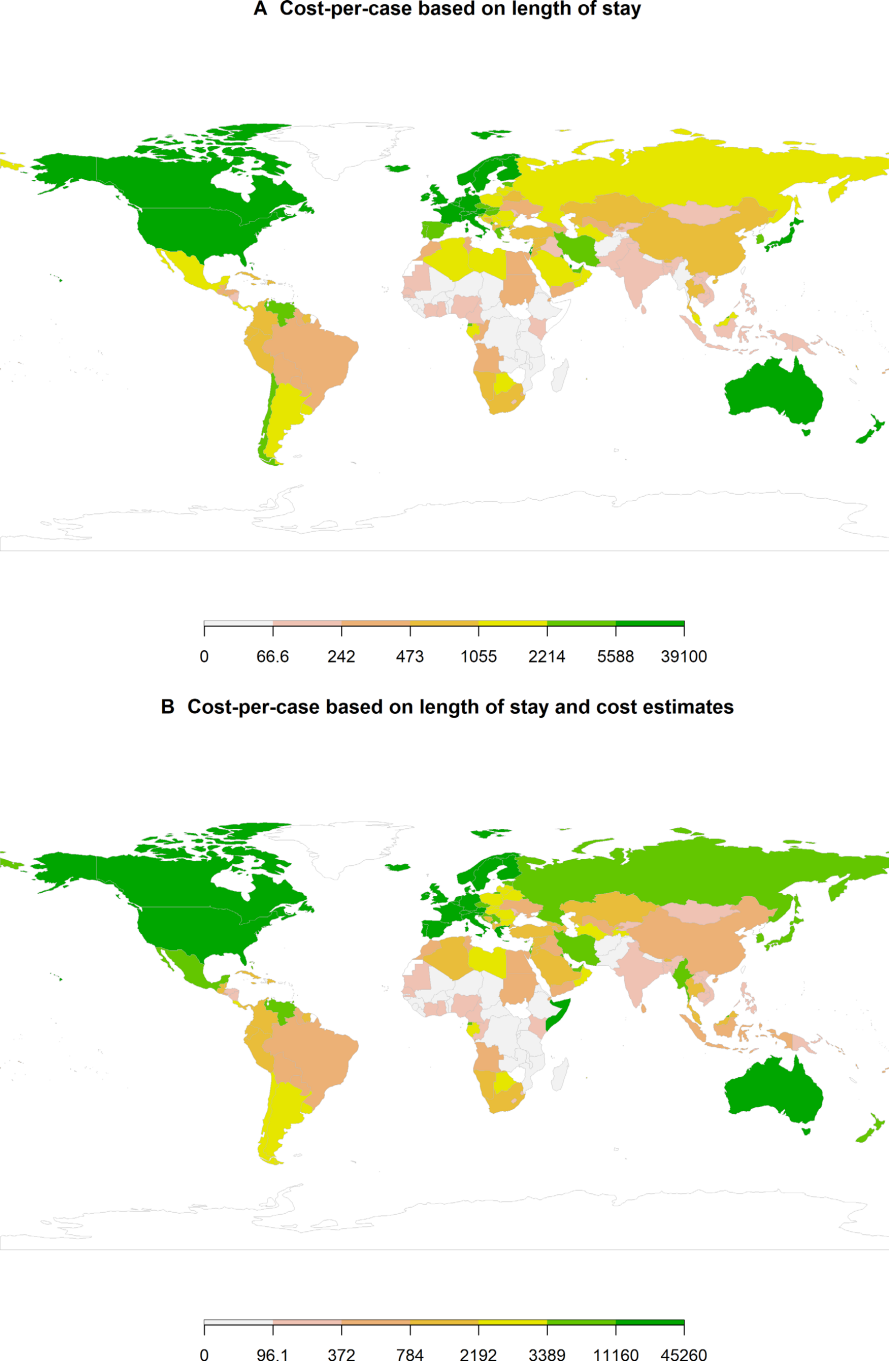
Hospital cost-per-case associated with antibiotic resistance from the meta-analyses. Median values, by country, of statistically significant cost-per-case estimates from the meta-analyses. Costs presented are in US$. (**A**) represents cost estimates based on LoS, combined with WHO-CHOICE, while (**B**) represents averaged values based on LoS and cost estimates from the literature. LoS, length of hospital stay.

There were 246 and 68 estimates for hospital cost-per-case/LoS impacts attributable to and associated with ABR, respectively, coming from 180 studies (see online supplemental material S6A for more information on data retrieval). There was a concentration of input data in the European Region (EUR) and Region of the Americas (AMR), particularly for 3gc and penicillin resistance impact estimates. The African Region (AFR) and Eastern Mediterranean Region (EMR) (in terms of WHO regions), and LMICs (in terms of income classification) have few data across exposure groups of interest, with no data found from LICs (see online supplemental material S6B). These data translated to 3441 estimates of the attributable ABR hospital cost per case and 2487 estimates for the associated ABR hospital cost per case, across individual countries.^20^ Figures2  3 highlight the heterogeneity in hospital cost-per-case estimates across countries and exposure groups of interest.

**Figure 3:**
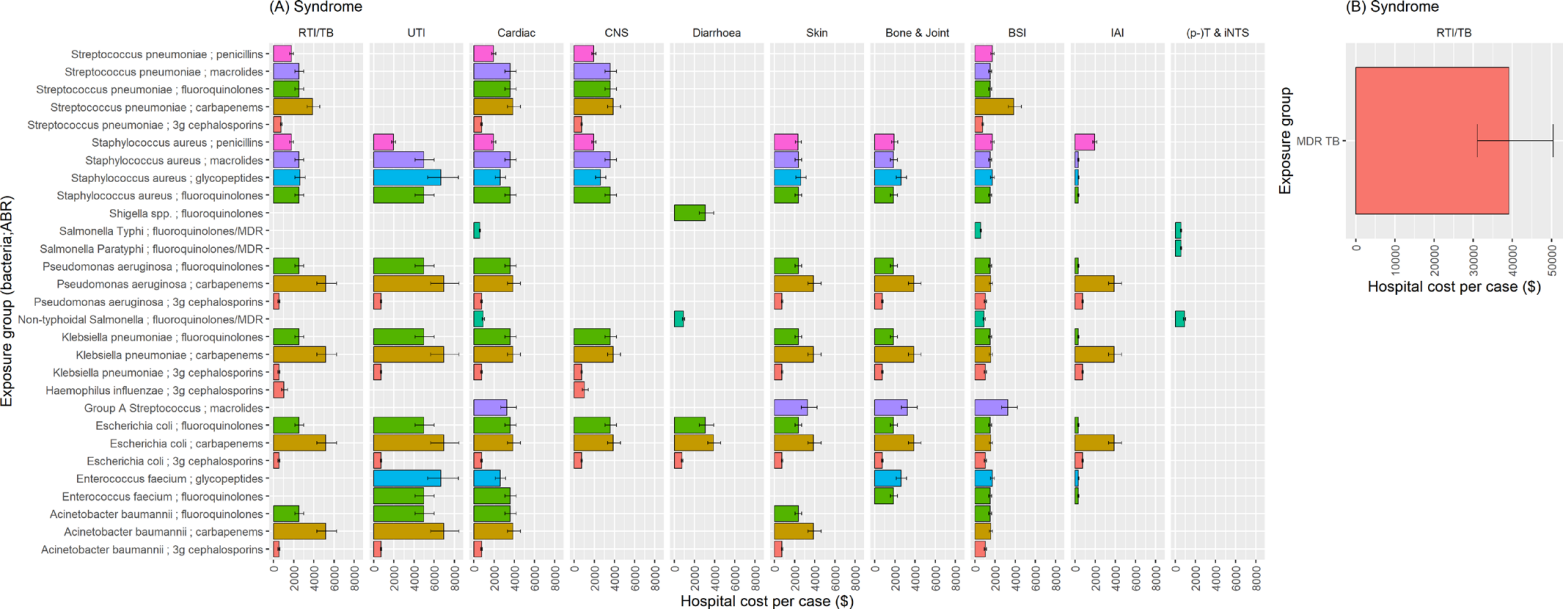
Global averages of hospital cost-per-case associated with antibiotic resistance. Exposure groups are defined by antibiotic (which they are resistant to) and bacteria. Colours represent the antibiotic class resistance (as defined by the exposure group legend). (**A**) Shows results for bacteria, (**B**) for mycobacteria. 3g, third generation; BSI, bloodstream infection; CNS, central nervous system; IAI, intra-abdominal infections; MDR, multidrug resistant; (p-)T & iNTS, (para-)/Typhoid/iNTS; RTI, respiratory tract infection; TB, tuberculosis; UTI, urinary tract infections.

Subsetting the meta-analysis modelling data to include only those where the mean value was equal to or greater than US$0, with a 95% UI not crossing zero, showed cost-per-case estimates ranged from US$2 (95% UI: US$7–US$16) and US$3 (US$7–US$12) for ABR attributable and associated hospital costs in Afghanistan to US$22 800 (95% UI: US$4900–US$57 900) and US$5900 (95% UI: US$12 400–US$147 200) for Monaco respectively, across differing drug-pathogen-syndrome combinations. MDR TB was the pathogen with the highest attributable ABR impact on hospital cost-per-case. MDR TB had the highest hospital cost per case attributable to ABR when aggregated to the regional level (US$40 695 (95% UI: US$2725–US$124 104) for EUR; US$22 964 (US$2181–US$106 518) for AMR; US$20 147 (US$553–US$89 595) for EMR; US$5942 (US$124–US$48 543) for the Western Pacific Region (WPR); US$4281 (US$254–US$30 796) for AFR; US$2951 (US$52–US$11 407) for the South-East Asia Region (SEAR)). In terms of LoS-based cost-per-case, Gram-negative respiratory tract infections (RTIs) that were carbapenem resistant had the highest cost per case that was associated with ABR in some regions (US$9648 (95% UI: US$169–US$44 357) for EUR; US$3810 (US$189–US$20 799) for WPR; US$3392 (US$218–US$18 055) AMR; US$3022 (US$23–US$18 810) for EMR), while carbapenem resistant Gram-negative BSIs had the highest cost per case for AFR and SEAR at US$680 (95% UI: US$17–US$3822) and US$666 (US$40–US$2472), respectively.

Once these data from the meta-analysis had been combined with the other sources and undergone the transformation processes (described in the Methods section and online supplemental material S5C), we generated the cost-per-case (based on LoS) estimates, as shown in figure 3. This shows, aside from MDR TB, that the average cost per case of carbapenem-resistant infections is higher (on average) than infections with resistance to other antibiotics. Carbapenem-resistant Gram-negative RTIs are still one of the leading exposure groups in terms of hospital cost per case. However, they were estimated to be behind carbapenem-resistant and glycopeptide-resistant urinary tract infections (UTIs), with resistant UTIs costing US$5000–US$7000 per case (global population-weighted average). Though patterns vary by syndrome, carbapenem, glycopeptide, fluoroquinolone and macrolide resistant infections have higher costs per case in comparison to penicillin and 3gc resistant infections. See online supplemental material S6C,D for antibiotic and labour productivity unit costs, respectively.

### Total hospital costs and labour productivity losses associated with ABR

**Figure 4:**
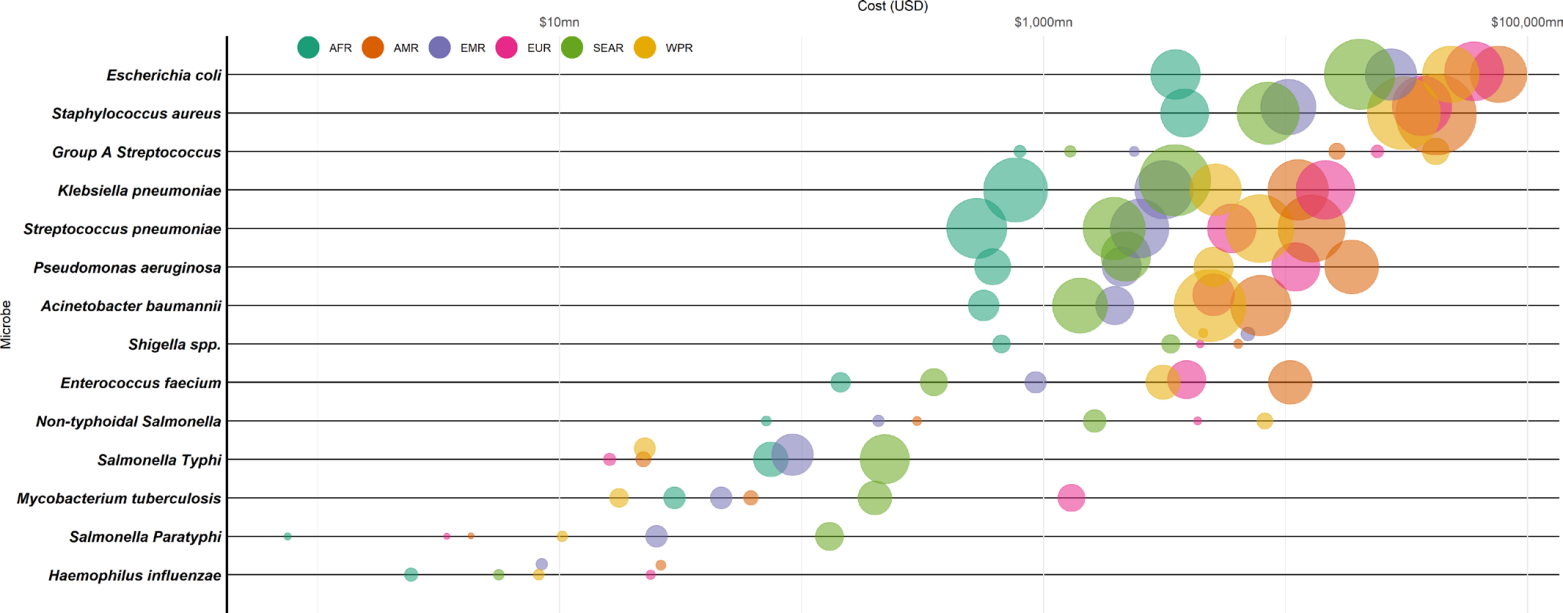
Regional costs in 2019 associated with antibiotic resistance. Log scaled costs in 2019 US$, mn=a million. Where the point on the y-axis represents hospital costs. Circles are sized by productivity loss due to deaths, and colour indicates WHO region. AFR, African Region; AMR, Region of the Americas; EMR, Eastern Mediterranean Region; EUR, European Region; SEAR, South-East Asia Region; WPR, Western Pacific Region.

We estimated the total hospital cost associated with ABR in 2019 to be US$693 billion (bn) (IQR: US$627 bn–US$768 bn) globally (table 1). As shown in figure 4, the two costliest pathogens to hospitals, globally, were estimated to be *E. coli* at US$246 bn (IQR: US$218 bn–US$275 bn) and *S. aureus* at US$135 bn (IQR: US$123 bn–US$149 bn). Infections resistant to fluoroquinolones and macrolides were estimated to cost the most to hospitals, with a combined cost of over US$488 bn (see online supplemental material S7).

**Table 1:** Global economic burden associated with antibiotic resistance in 2019 and potential vaccine-avertable burden

Pathogen	Scenario description	Hospital cost associated with ABRUS$ median (IQR)	Potential hospital cost associated with ABR avertedUS$ median (IQR)	Productivity loss associated with ABRUS$ range*	Potential productivity loss associated with ABR avertedUS$ range*
*Acinetobacter baumannii*	70% coverage, 70% efficacy, 5-year duration, for all age groups and syndromes	22 568 116 422(20 634 903 828–24 619 245 871)	11 058 377 047(10 111 102 876–12 063 430 477)	21 309 653 397–**22 743 692 012**	10 441 730 164–**11 144 409 086**
*Escherichia coli (EXPEC)†*	70% coverage, 70% efficacy, 5-year duration, for all age groups and syndromes (except diarrhoea)	245 657 842 455(217 752 133 181–274 677 722 490)†	92 567 527 534(81 168 808 341–105 348 037 460)	**27 082 484 206**–27 291 358 658	**13 270 417 261**–13 372 765 742
*E. coli (ETEC)†*	70% coverage, 60% efficacy, 5-year duration, for 6-month age group and diarrhoea		1 578 793 147(1 357 786 900–1 804 262 076)	**1 756 773 012**–2 055 454 589	**176 425 010**–219 056 457
*Enterococcus faecium*	70% coverage, 70% efficacy, 5-year duration, for all age groups and all syndromes	19 485 624 438 (16 283 763 580–24 004 458 758)	9 547 955 974(7 979 044 154–11 762 184 791)	7 310 722 756–**7 506 043 496**	3 582 254 151–**3 677 961 313**
*Group A Streptococcus*	70% coverage, 70% efficacy, 5-year duration, for 6 weeks age group and all syndromes	96 431 281 746(74 597 864 859–127 810 375 648)	3 117 950 567(2 278 521 927–4 281 512 425)	**1 216 497 723**–1 137 852 032	**65 635 670**–67 991 155
*Haemophilus influenzae*	90% coverage, varying efficacy‡, 5-year duration for 6, 10, 14 weeks age group and all syndromes	82 668 991(67 245 397–109 218 073)	7 160 930(5 965 238–8 983 971)	**312 025 523**–388 598 152	**99 092 996**–130 941 178
*Klebsiella pneumoniae*	70% coverage, 70% efficacy, 5-year duration, for all age groups and syndromes	40 069 981 315(36 469 471 746–44 097 943 142)	19 634 290 844(17 870 041 156–21 607 992 139)	**32 766 920 728**–35 748 953 876	**16 055 791 157**–17 516 987 399
*Mycobacterium Tuberculosis*	Improved: 70% coverage, 80% efficacy, 10-year duration, for 0 weeks age group+boost every 10 years and all syndromes	1 732 198 682(1 403 730 108–2 082 111 755)	970 031 262(786 088 860–1 165 982 583)	**3 568 859 707**–3 601 277 832	**1 998 561 436**–2 016 715 586
*Non-typhoidal Salmonella*	70% coverage, 80% efficacy, 5-year duration, for 6 weeks and 9 months age group and all syndromes	15 299 829 728(12 611 740 072–18 480 360 750)	1 489 592 674(1 223 804 384–1 772 096 050)	**809 123 748 **–87 435 205	87 435 205–93 943 029
*Pseudomonas aeruginosa*	70% coverage, 70% efficacy, 5-year duration, for all age groups and BSI and RTI syndromes	40 814 388 132(35 982 662 722–46 208 751 890)	4 576 961 347(4 056 344 554–5 318 484 034)	**16 478 686 339**–16 709 150 388	**5 952 753 447**–6 157 790 695
*Salmonella Typhi*	70% coverage, 85% efficacy, 15-year duration, for 9 months age group and all syndromes	459 541 933(406 439 151–536 592 455)	110 545 318(94 640 068–133 958 002)	**7 864 105 976**–8 334 482 816	**2 300 990 421**–2 312 517 736
*Salmonella Paratyphi*	70% coverage, 70% efficacy, 5-year duration, for 9 months age group and all syndromes	178 404 588(146 453 229–224 190 284)	6 492 057 (5 326 979–8 187 912)	1 162 490 387–**1 378 353 646**	73 695 288–86 685 792
*Shigella spp*	70% coverage, 60% efficacy, 5-year duration, for 6 months age group and all syndromes	30 443 436 977(24 902 777 578–37 625 834 835)	1 553 347 754(1 285 343 697–1 918 604 035)	**723 242 802–**898 487 822	**157 605 018**–215 139 771
*Staphylococcus aureus*	70% coverage, 60% efficacy, 5-year duration, for all age groups and all syndromes	135 262 279 776(122 892 761 884–149 309 751 036)	56 810 157 506(51 614 959 991–62 710 095 435)	36 382 697 954–**36 459 809 770**	15 280 733 141–**15 313 120 103**
*Streptococcus pneumoniae*	Improved: 90% coverage, varying efficacy‡, 5-year duration for 6 weeks and elderly age groups and BSI, CNS infections, cardiac infections, LRI syndromes	33 427 525 293(29 065 480 794–38 195 875 350)	2 002 889 897(1 774 917 602–2 285 983 440)	**32 833 638 622**–34 975 263 155	**6 041 167 929**–6 909 676 852

* The range presented in productivity loss estimates represents the two values estimated (1) using employment and trend adjustment —the base case scenario (in bold) and (2) GDP deflation adjusted with no trend adjustments.

† Total amount of hospital cost estimated for all *E. coli* (EXPEC and ETEC).

‡ See online supplemental material S5 table 5table 5 for more detail.

ABR, antibiotic resistance; BSI, bloodstream infection; CNS, central nervous system; ETEC, Enterotoxigenic *E. coli*; EXPEC, extraintestinal pathogenic *E. coli*; LRI, lower respiratory infections; RTI, respiratory tract infection; TB, tuberculosis.

Deaths due to these infections were estimated to cost around US$194 bn in productivity losses globally (US$198 bn in a scenario analysis where latest values were inflated based on GDP deflation rather than trend-adjusted for those without 2019 values). *S. aureus* was a leading cause of ABR associated productivity losses of US$36bn globally, followed by *S. pneumoniae* and *K. pneumoniae* of US$33bn each. This differed depending on region, with the top costs associated with lost labour productivity being associated with *S. aureus* for AMR, EUR and WPR, *K. pneumoniae* for AFR and *S. pneumoniae* for EMR.

### Vaccine impact on hospital costs and labour productivity losses associated with ABR

**Figure 5:**
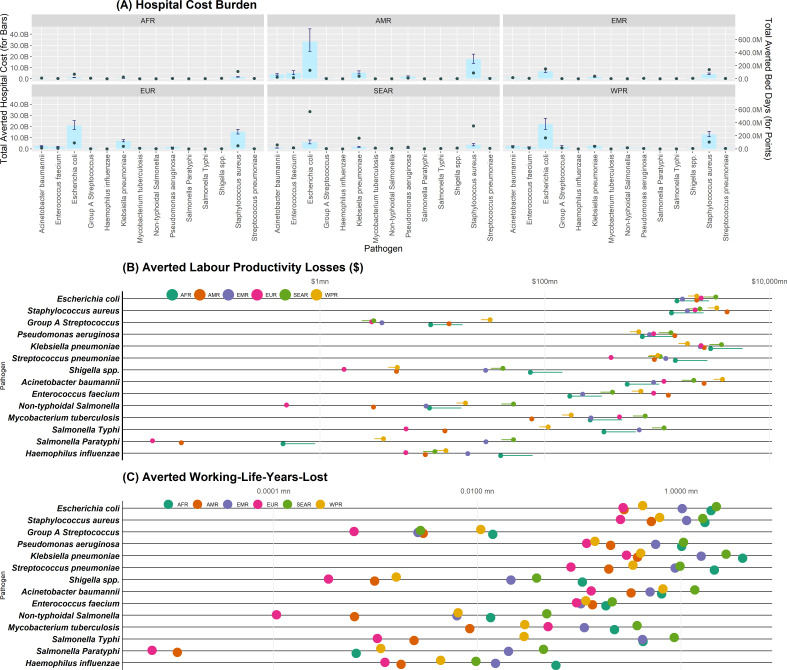
Potential vaccine avertable burden associated with ABR in 2019. (**A**) Hospital cost burden. Panels by WHO region. The primary y-axis relates to the bar plots, which represent median total hospital cost averted, while corresponding error bars represent the IQR of hospital cost ($), where B=billions. The secondary axis is related to the plotted points (total averted bed days), where M=millions. (**B**) Productivity Loss Burden–Costs ($). Point estimates are results in base case scenario, with the corresponding line representing min and max values across scenarios (trend adjusted vs non-trend adjusted). (**C**) Productivity loss burden–working-life-years-lost. ABR, antibiotic resistance; AFR, African Region; AMR, Region of the Americas; EMR, Eastern Mediterranean Region; EUR, European Region; SEAR, South-East Asia Region; WPR, Western Pacific Region.

The global economic burden associated with ABR in 2019 and potential, hypothetical, vaccine-avertable burden is shown in table 1, alongside the assumptions made in relation to vaccine and implementation characteristics. Vaccines could have potentially averted US$2.8 bn bed-days (IQR: US$2.7 bn–3.0 bn), equivalent to US$207 bn (IQR: US$186 bn–US$229 bn) in hospital costs, in 2019. While AMR, EUR and WPR regions have higher hospital costs associated with antibiotic resistant *S. aureus* and *E. coli infections* than other regions, the projected impact of vaccines was higher in SEAR, EMR and WPR in terms of bed-days averted (see figure 5A).

The modelled vaccine scenarios could have potentially averted US$76 bn in productivity losses (see figure 5B across pathogens and regions). In SEAR and AMR, vaccination would avert around US$40 bn per region in productivity losses (the most gains). Undiscounted WLYL was highest in AFR at 23 million WLYL (figure 5C). Under the chosen hypothetical scenarios, vaccines against *K. pneumoniae,* followed by *S. aureus,* could avert the most productivity losses globally. However, the vaccine that would avert the most productivity losses differed across regions (online supplemental material S7). For AMR (the region), this was a vaccine against *S. aureus*, for WPR it was *A. baumannii*, for EUR it was *E. coli*, while for AFR, EMR and SEAR it was *K. pneumoniae*.

In terms of syndromes, most hospital costs could be averted for UTIs (US$116 bn) and skin infections (US$53 bn), while the most labour productivity costs could be averted for RTIs (US$27 bn) and BSIs (US$25 bn).

## Discussion

We estimate ABR was associated with almost US$700 bn in hospital costs globally in 2019. We estimated productivity losses at over US$193 bn due to excess deaths associated with ABR. We estimate that, under the hypothetical vaccine scenarios modelled, 30% of hospital costs (around US$200 bn) and 40% of costs associated with productivity losses (almost US$80 bn) could have been averted. *S. aureus, E. coli* and *K. pneumoniae* vaccines would avert the most hospital costs (US$169 bn in total across all 3) and productivity loss (US$44 bn), globally.

This is the first study to provide hospital cost-per-case and total, annual burden estimates by WHO region and exposure group of interest. We inferred that vaccines which target antibiotic-resistant BSIs and RTIs would likely avert the most productivity losses, driven by reductions in fatal infections, particularly in neonatal populations. For hospital costs, UTIs and skin infections make up a large proportion of vaccine avertable costs, largely driven by large case numbers.

A prior meta-analysis found that the attributable cost to ABR ranged from −US$2371 to +US$29 289 per patient episode, with the mean excess LoS estimated at 7.4 days (95% CI 3.4 to 11.4).^2^ However, this study did not adjust for (beyond the use of random effects) heterogeneity in exposure group or setting. The estimates of this paper are aligned with a recent global study which estimated ABR hospital cost-per-case attributable to ABR ranged from around US$100–US$20 000 per case, on average.^36^ On a hospital cost per-case basis, at the global level, we estimated that MDR-TB and carbapenem-resistant infections were most costly. However, more observational data in relation to hospital costs associated with ABR exposures, particularly in LICs, are needed to fill the evidence gaps. Again, this aligns with previous literature that suggests antibiotic-resistant TB infections are the most costly in terms of hospital admissions.^36^ Our total, global ABR associated burden for hospital costs (around US$700 bn) is much higher than previously estimated (almost US$70 bn). This is likely because (1) we estimate associated (total infection) cost burden while previous estimates are of attributable (excess) cost burden and (2) previous estimates assume a lower number of hospital admissions related to resistant infections (at 25 million, which would be less than 20% of ABR associated healthcare-acquired infection estimated to occur globally every year).^36 37^ More research on the likelihood of hospitalisation based on infection type and setting is needed.

Our study has limitations. First, the meta-analyses for hospital cost per case were based on a review of reviews, meaning data are dependent on the different search strategies and inclusion criteria utilised across these reviews. For example, there may be heterogeneity in ABR definitions and scope of cost inclusion between studies reviewed, which may have added additional uncertainty to the cost estimates. Our results rely on data from previous systematic reviews, which themselves are further dependent on the way individual studies were conducted. For example, there might be potential for double-counting if they included patients with multiple syndromes due to resistant organisms and do not adjust for these accordingly. Additionally, excluding studies based on the quality of statistical methods was not done, which can lead to overestimation of attributable costs if time dependency is not adequately accounted for. Although grouping exposure groups of interest by Gram-stain, antibiotic class and region was used to maximise study data while maintaining relevance, most summary effect measures were based on a small number of studies. In some instances, the cost per case estimate is possibly non-robust due to there being very large differences across effect estimates that were extracted within exposure groups of interest, potentially due to heterogeneity in study populations and statistical methods. Our process allows for a larger sample and has partly accounted for these issues through random effects processes. Second, pooling hospital costs across countries is a debatable procedure.^38^ Another limitation is the geographical limitations in the data. We often had to map findings from a single country to an entire region, particularly for LMIC, AFR and EMR regions. We, therefore, provide cost-per-case estimates based on just the meta-analyses of LoS, as well as the meta-analyses of costing studies. Though, in doing so, converting LoS estimates to cost-per-case required the use of WHO-CHOICE estimates, which could lead to an underestimation as these exclude the costs of drugs and diagnostic tests.^26^ More primary cost studies in LMICs would enable countries to estimate their own hospital costs without needing to rely on pooling estimates or WHO-CHOICE. Third, we calculate the unit cost of antibiotics, but do not include this in our total burden estimation process, as calculating the relevant antibiotic course for each exposure group in each setting was beyond the scope of this study. However, previous studies suggest bacterial vaccines do have the power to substantially reduce antibiotic use,^16 17^ and future work could combine our work with such estimates to understand these wider cost impacts of vaccines on antibiotic use. Additionally, our labour productivity costs were estimated using the human capital method which relies on the employment ratio and wage rate in each country. However, these data were unavailable for 26% of countries. We imputed the missing data using both trend analyses and inflation adjustment, though uncertainty due to this is not propagated through the model. Our estimate of labour productivity losses may also be an underestimate since we used mean nominal monthly earnings of employees and employment-to-population ratios. In many LMICs, there is potentially a substantial informal economic sector that is not fully captured in employment figures. Other key limitations include the exclusion of modelling changes in the evolution of resistance and of patient-borne and/or caregiver costs. By providing code and data open access, we allow for capacity building in the field of economic evaluations of ABR.

In relation to our total burden estimation for 2019, our analysis is limited to a 1-year (2019) impact time horizon. This is in keeping with the time horizon used in the recent WHO vaccines and antimicrobial resistance assessment.^39^ Hence, we did not try to project how these costs may change in the future cohorts. Some of these costs (such as productivity costs) may have been affected by the economic impact of the COVID-19 pandemic. We sampled the meta-analyses results using a truncated normal distribution to combine these with epidemiological estimates, potentially incorporating bias based on set upper and lower bounds. Future work could integrate Bayesian methods into the meta-analysis and modelling processes. The epidemiological estimates were based on point estimates from a static model with its own limitations,^11^ including potentially high coverage, efficacy and duration scenarios in hypothetical vaccine scenarios used, for many of which current vaccines are not available. In some cases, they represent hypothetical assumptions (eg, WHO preferred product characteristics) rather than actual clinical trial or field study findings.^39^ More robust dynamic transmission models and resource use studies (including on hospitalisation rates) for ABR-associated pathogens and syndromes will help ensure future vaccine evaluation appropriately considers ABR.

Care should be taken in using our regional estimates for cross-regional comparisons of societal costs, given the ethical implications of valuing life differently based on wages. Using capacity measures such as WLYL and bed-days lost alongside such estimates provides a more extensive picture. Additionally, vaccines provide one element of a comprehensive infection, prevention and control strategy. Vaccines are an important intervention to reduce the economic burden of ABR and have a key role alongside other infection control measures such as antibiotic stewardship and water, sanitation and hygiene programmes. These other interventions are particularly important to prevent ABR from worsening in pathogens against which vaccines are still being developed. Future research to translate these impacts into economic burden estimates, such as those presented here, would be useful in understanding efficient combinations of such measures in relation to ABR.^10 12^

Our study shows that ABR is associated with significant hospital costs and can markedly reduce labour-supply-related productivity, and these associated costs can be substantially reduced by vaccination. The economic burden of ABR and vaccine impact varies substantially across drug-pathogen-syndrome-setting combinations. Vaccines preventing antibiotic-resistant BSIs and RTIs have relatively the highest impact on reducing productivity losses. Vaccines preventing antibiotic-resistant UTIs and skin infections have relatively the highest impact on reducing hospital costs. Specifically, vaccines against *S. aureus, E. coli* and *K. pneumoniae* would avert a substantial portion of the economic burden associated with ABR.

## Supplementary material

10.1136/bmjgh-2024-016249 online supplemental file 1

## Data Availability

Data are available in a public, open access repository.
