# Supplementary material for: The global economic burden of antibiotic-resistant infections and the potential impact of bacterial vaccines: a modelling study
Source: BMJ Glob Health. 2025 Jun 19;10(6):e016249. doi: 10.1136/bmjgh-2024-016249 (PMC12182023; doi:10.1136/bmjgh-2024-016249)
Supplement: online supplemental file 1 [file bmjgh-10-6-s001.pdf]

Supplementary Material for The Global Cost of Antibiotic Resistant Infections and the Potential Impact of Bacterial Vaccines: An Economic Modelling Study

Contents

Supplementary Material for The Global Cost of Antibiotic Resistant Infections and the Potential Impact of Bacterial Vaccines: An Economic Modelling Study 1

S1. CHEERS 2022 Checklist 2

S2. Additional Information on the Rapid Review of Systematic Reviews 4

A. Mapping Data to Gram, antibiotic class, syndromes and regional classifications 4

B. Exposure Impact Transformation Formulae 4

C. Additional Retrieval and Inclusion Information 5

D. Stepwise and Sampling Calculations Performed to Estimate Hospital Cost per Case 7

S3. Estimating Antibiotic Costs and Productivity Costs 7

A. Additional information on antibiotic cost estimation 7

B. Additional information on productivity cost estimation 8

S4. Adjustment Factors for length-of-stay based costs 8

S5. Methods for Combining Unit Costs and Epidemiological Estimates 9

A. Vaccine Scenarios 9

B. Expert elicitation exercise performed by WHO 9

C. Sampling unit costs from meta-analysis 11

D. Proportions of cases hospitalised 12

E. Labour productivity losses calculations 13

S6. Additional Cost Results 13

A. Data retrieval and completeness 13

B. Hospital cost per case estimates 17

C. Antibiotic unit costs 24

D. Unit productivity costs estimates 27

S7. Additional 2019 burden and vaccine impact results 28

S8. References 81

S1. CHEERS 2022 Checklist

Topic	No.	Item	Location where item is reported
**Title**			
	1	Identify the study as an economic evaluation and specify the interventions being compared.	Title, Page1
**Abstract**			
	2	Provide a structured summary that highlights context, key methods, results, and alternative analyses.	Abstract, Page 1
**Introduction**			
**Background and objectives**	3	Give the context for the study, the study question, and its practical relevance for decision making in policy or practice.	Introduction, Pages 1 -2
**Methods**			
**Health economic analysis plan**	4	Indicate whether a health economic analysis plan was developed and where available.	Not Applicable
**Study population**	5	Describe characteristics of the study population (such as age range, demographics, socioeconomic, or clinical characteristics).	Methods, Pages 2-4
**Setting and location**	6	Provide relevant contextual information that may influence findings.	Methods, Pages 2-4
**Comparators**	7	Describe the interventions or strategies being compared and why chosen.	Introduction, Page 2 & Methods, Pages 2-4
**Perspective**	8	State the perspective(s) adopted by the study and why chosen.	Methods, Pages 2-4
**Time horizon**	9	State the time horizon for the study and why appropriate.	Methods, Page 4
**Discount rate**	10	Report the discount rate(s) and reason chosen.	Methods, Page 4
**Selection of outcomes**	11	Describe what outcomes were used as the measure(s) of benefit(s) and harm(s).	Methods, Pages 2-4
**Measurement of outcomes**	12	Describe how outcomes used to capture benefit(s) and harm(s) were measured.	Methods, Pages 2-4
**Valuation of outcomes**	13	Describe the population and methods used to measure and value outcomes.	Methods, Pages 2-4
**Measurement and valuation of resources and costs**	14	Describe how costs were valued.	Methods, Pages 3-4
**Currency, price date, and conversion**	15	Report the dates of the estimated resource quantities and unit costs, plus the currency and year of conversion.	Methods, Page 4 & online supplementary material
**Rationale and description of model**	16	If modelling is used, describe in detail and why used. Report if the model is publicly available and where it can be accessed.	Methods, Pages 3-4
**Analytics and assumptions**	17	Describe any methods for analysing or statistically transforming data, any extrapolation methods, and approaches for validating any model used.	Methods, Pages 3-4

Topic	No.	Item	Location where item is reported
**Characterising heterogeneity**	18	Describe any methods used for estimating how the results of the study vary for subgroups.	Methods, Pages 3-4
**Characterising distributional effects**	19	Describe how impacts are distributed across different individuals or adjustments made to reflect priority populations.	Methods, Pages 3-4
**Characterising uncertainty**	20	Describe methods to characterise any sources of uncertainty in the analysis.	Methods, Page 4
**Approach to engagement with patients and others affected by the study**	21	Describe any approaches to engage patients or service recipients, the general public, communities, or stakeholders (such as clinicians or payers) in the design of the study.	Not Applicable/ expert engagement described in online supplementary material
**Results**			
**Study parameters**	22	Report all analytic inputs (such as values, ranges, references) including uncertainty or distributional assumptions.	Results, pages 4-8
**Summary of main results**	23	Report the mean values for the main categories of costs and outcomes of interest and summarise them in the most appropriate overall measure.	Results, pages 4-8 & Figures 2-5
**Effect of uncertainty**	24	Describe how uncertainty about analytic judgments, inputs, or projections affect findings. Report the effect of choice of discount rate and time horizon, if applicable.	Results, Table 1
**Effect of engagement with patients and others affected by the study**	25	Report on any difference patient/service recipient, general public, community, or stakeholder involvement made to the approach or findings of the study	Not reported
**Discussion**			
**Study findings, limitations, generalisability, and current knowledge**	26	Report key findings, limitations, ethical or equity considerations not captured, and how these could affect patients, policy, or practice.	Discussion, pages 8-11
**Other relevant information**			
**Source of funding**	27	Describe how the study was funded and any role of the funder in the identification, design, conduct, and reporting of the analysis	Page 11
**Conflicts of interest**	28	Report authors conflicts of interest according to journal or International Committee of Medical Journal Editors requirements.	Page 11

From: Husereau D, Drummond M, Augustovski F, et al. Consolidated Health Economic Evaluation Reporting Standards 2022 (CHEERS 2022) Explanation and Elaboration: A Report of the ISPOR CHEERS II Good Practices Task Force. Value Health 2022;25. doi:10.1016/j.jval.2021.10.008

S2. Additional Information on the Rapid Review of Systematic Reviews

A. Mapping Data to Gram, antibiotic class, syndromes and regional classifications

Bacterial exposure groups of interest are outlined in Table 1.

Table 1. Drug-Pathogen-Syndrome Combinations

Abbreviations: 3g – third-generation, BSI – bloodstream infection, CNS – central nervous system, LRI – lower respiratory tract infection, UTI – urinary tract infections

Pathogen	Syndrome	Antibiotic class
Acinetobacter baumannii	BSI, Bacterial skin infections, Cardiac infections, LRI and thorax infections, UTI	3g cephalosporins, carbapenems, fluoroquinolones
Enterococcus faecium	BSI, Bone and joint infections, Cardiac infections, Intra-abdominal infections, UTI	fluoroquinolones, glycopeptides
Escherichia coli	BSI, Bacterial skin infections, Bone and joint infections, CNS infections, Cardiac infections, Diarrhoea, Intra-abdominal infections, LRI and thorax infections, UTI	3g cephalosporins, carbapenems, fluoroquinolones
Group A Streptococcus	BSI, Bacterial skin infections, Bone and joint infections, Cardiac infections	macrolides
Haemophilus influenzae	CNS infections, LRI and thorax infections	3g cephalosporins
Klebsiella pneumoniae	BSI, Bacterial skin infections, Bone and joint infections, CNS infections, Cardiac infections, Intra-abdominal infections, LRI and thorax infections, UTI	3g cephalosporins, carbapenems, fluoroquinolones
Mycobacterium tuberculosis	Tuberculosis	Multi-drug resistance
Pseudomonas aeruginosa	BSI, Bacterial skin infections, Bone and joint infections, Cardiac infections, Intra-abdominal infections, LRI and thorax infections, UTI	3g cephalosporins, carbapenems, fluoroquinolones
Salmonella Paratyphi & TYPHI & Non-typhoidal Salmonella	BSI, Cardia infections, Typhoid, paratyphoid, and iNTS	Fluoroquinolones or MDR in Salmonella
Shigella spp.	Diarrhoea	Fluoroquinolones
Staphylococcus aureus	BSI, Bacterial skin infections, Bone and joint infections, CNS infections, Cardiac infections, Intra-abdominal infections, LRI and thorax infections, UTI	fluoroquinolones, glycopeptides, macrolides, penicillins
Streptococcus pneumoniae	LRI and thorax infections, BSI, CNS infections, Cardiac infections	3g cephalosporins, carbapenems, fluoroquinolones, macrolides, penicillins

Bacteria were mapped to their Gram stain (Gram-positive, Gram-negative or Tuberculosis)[1] (see "bug_gram.csv" in NikkiR08/AMR-UCR: Antimicrobial Resistance Unit Costs (github.com)). Individual antibiotics were mapped to antibiotic classes[2-6]. Assumptions were made about enzyme production as an exposure definition; for example, studies noting extended-spectrum-beta-lactamase (ESBL+) were mapped to 3GC resistance. Carbapenemases were found to cover ESBL and carbapenems, but this was linked to carbapenem antibiotic group so as to not bias the 3GC resistance estimates of impact upwards. Countries were mapped to WHO-CHOICE, WHO and World Bank income and geographical regions [7,8]. Note that certain countries appear on some lists and not others, the final list of countries linked to regions had to appear in all. Some papers which were used to extract hospital costs from were based in "Europe", which spans different regions depending on the definition of region used. We therefore grouped these estimates by the World Bank (WB) Geographic region, which was then mapped to 1 country (first in the list) in any respective groups that are covered within the area (e.g., 1 EURO A, 1 EURO B, 1 upper income). When using WHO regional definitions, only 1 low-income country and 4 LMIC countries out of 51 (<10%) were present in Europe, therefore Europe results were not mapped to "low income" and "low and middle income" so as not to skew other low/low-middle income country cost results towards potentially higher European costs. To map back down Gram-stain and syndrome combination estimates the clinical literature was consulted as to causative microbes for syndromes[1]. For example, the LoS impact of a Gram-negative urinary tract infection would be mapped back down to *E. coli* impacts, but not *Campylobacter.*

Countries were then assigned data for their region based on the following hierarchy, in order to use the most relevant data: 1. WHO-CHOICE region, 2. WB Income region, 3. WB geographic region, and if no data were available by any of these then, 4. Global. WHO-CHOICE and Income groups were preferential to geographic region as they are indicators of more similar healthcare systems either based on outcomes (WHO-CHOICE) or resource available for healthcare spending (Income group).

B. Exposure Impact Transformation Formulae

Reliant on the assumption of normality, if a mean (μ) was given, the following were performed, dependent on the information available per study[9].

- Converting standard deviation (SD) to standard error (SE) with sample size (N) available:

$$SE = SD/\sqrt{N}$$

- Converting 95% confidence intervals to standard error:

$$SE = (upper\ 95\%\ confidence\ interval\ limit - lower\ 95\%\ confidence\ interval\ limit)\ /\ 3.92$$

- Converting a p-value to standard error:

$$SE = \mu / abs(qnorm(pvalue/2))$$

If a median value was given, the bellow formulae were used to estimate mean treatment effects, based on those available within *Wan et al 2014*[10]. See *Wan et al* 2014 directly for corresponding calculations for SE derivation.[10]

- For when median, range and interquartile range (IQR) values are available:

$$x = \frac{([Minimum\ value] + [Low\ IQR] + [median] + [High\ IQR])/4) + ([4 * Maximum\ value] - [Minimum\ Value] - [Low\ IQR] - [Median] - [High\ IQR])}{4 * N}$$

$$y = \frac{([Low\ IQR] + [median] + [High\ IQR] + [Maximum\ value])/4) + ([4 * Minimum\ Value] - [Low\ IQR] - [Median] - [High\ IQR] - [Maximum\ value])}{(4 * N)}$$

$$\mu = \frac{(x + y)}{2}$$

- For when median and range variable are available:

$$\mu = \frac{[Low\ IQR] + (2 * Median) + [High\ IQR]}{4} + \frac{[Low\ IQR] + (2 * Median) + [High\ IQR]}{4 * N}$$

- For when median and IQR values are available:

$$\mu = \frac{[Low\ IQR] + [Median] + [High\ IQR]}{3}$$

If these were calculated for the exposed and unexposed groups separately the following formula was used to estimate the exposure effect (to then be input in the meta-analyses processes):

$$\mu_{ABR} = \mu_{exposed} - \mu_{unexposed}$$

$$SE_{ABR} = \sqrt{(SE_{exposed})^2 - (SE_{unexposed})^2}$$

To convert cost estimates of different currencies and of different cost years from the literature into 2019 USD (United States Dollar), the World Bank data for Purchasing Power Parity exchange rates, local currency unit exchange rates was used and IBAN data was used for local currency code matched to country[11,12]. The inflation and exchange rate conversion process took cost estimates and (i) converted from study currency to national, local currencies, (ii) inflated based on national GDP deflation data (or Eurozone equivalents if applicable) and (iii) subsequently converted into USD. If data were missing, USD values and US GDP deflation estimates were used. "PA.NUS.PPP", "PA.NUS.FCRF" and "NY.GDP.DEFL.ZS" and "NY.GDP.DEFL.KD.ZG" indicators were utilised from the "wbstats" package for the inflation calculations throughout this study.[11]

C. Additional Retrieval and Inclusion Information

To find systematic reviews for direct cost estimates of antimicrobial resistance (AMR) impact on healthcare systems WebofScience was searched on 4th September 2019 using the following search strategy: "TITLE: (cost or economic or length or stay or burden) AND TITLE: (antibiotic or antimicrobial) AND TITLE: (resistance or resistant) AND TITLE: (review)". This returned 13 results. The reasons for the exclusion of 9 studies: 2 were protocols, 2 were conference abstracts/proceedings, 1 only estimated mortality impact, 1 was a market-review, 2 were letters, and was a review on the effectiveness of a diagnostic.

Table 2 describes the inclusion/exclusion criteria applied to finding the systematic reviews. This led to 4 systematic literature reviews that estimate the monetary cost of AMR being included; Naylor[13], Founou[14], Zhen[15], Wozniak[16]. Additionally, Cassini et al 2018 [17] was utilised as a source for excess length of stay estimates, as known by the authors as a key resource for such impact estimates.

Table 2. Inclusion and Exclusion Criteria Applied for the Rapid Review of Systematic Review

Screening Stage	Criteria
Title screening	Include if mentioned bacteria of interest OR antibiotic resistance, plus mention of burden (not including mention of mortality and/or incidence/prevalence only)
	Exclude if a general "update"/review on the status of treatment/general literature
	Exclude if just epidemiology/microbiology/transmission focused
	Exclude if not related to human infections/colonization
	Exclude meeting/conference abstracts
	Include if English language
Abstract screening	Include if mention of antibiotic resistance and a mention of cost or length of stay outcome measures
	Exclude if focused on microbe which is not an exposure of interest
	Exclude if not related to human infections/colonization
	Exclude meeting/conference abstracts
Full Text Screening	Include if cost per case estimates or length of hospital stay estimates for antibiotic for microbes of interest
	Include if English language

The scope of bacteria profiles investigated were outlined by the World Health Organisation (WHO) Value Attribution Framework [18], these included; *Acinetobacter baumannii*, *Campylobacter, Chlamydia, Cholera*, *Clostridium difficile*, *Enterococcus faecium*, *Escherichia coli*, *Klebsiella pneumonia*, *Mycobacterium tuberculosis* (TB), *Pseudomonas aeruginosa*, *Salmonella typhi*, *Salmonella paratyphi*, *non-typhoidal Salmonella, Shigella spp, Staphylococcus aureus* and *Streptococcus pneumonia.* Unfortunately, on review of the systematic reviews found, data were lacking from the systematic review data extraction processes on bacteria related to gastro-related illnesses or sexual-transmitted diseases (such as *Campylobacter, Shigella, Helicobacter pylori, Neisseria gonorrhoeae* and *Chlamydia*). Rapid reviews with targeted search terms, such as "TOPIC: (Campylobacter) AND TOPIC: ((cost or economic or length or stay or burden)) AND TOPIC: ((resistance or resistant))" in Web of Science, were performed at the end of 2019. However, these generally focused on duration of illness (not necessarily alongside hospitalisation) without primary estimation of monetary cost, and therefore hard to integrate with the rest of the cost estimates calculated within this study. Relevant data were also not readily accessible for meningococcal and Group A/B Streptococcus antibiotic resistance impact in terms of unit costs. Additionally, epidemiology estimates on vaccine impact were not available for *Chlamydia, Cholera* and *H. pylori.* Given no data were available for the cost of resistance for hospitals for STIs found through these reviews, *Neisseria gonorrhoeae* was also not included in the final study.

Individual studies cited in the systematic reviews were included or excluded based on the criteria listed in Table 3. If included, the following variable were extracted for each study point of interest: retrieval; study reference; microbe; bacteria code; exposure definition; syndrome; first author(s); study year; study period; study type; country; number exposed; number of nonexposed; length of stay dummy; cost year; cost currency; measure of average impact; standard error/uncertainty measure; average for exposed; low uncertainty estimate for exposed; high uncertainty estimate for exposed; other uncertainty estimate for exposed; average for non-exposed; low uncertainty estimate for non-exposed; high uncertainty estimate for non-exposed; other uncertainty estimate for non-exposed; p-value; minimum value exposed; maximum value exposed; minimum value non-exposed; maximum value non-exposed; does it have both LoS and cost estimates and if so a unique id across the two rows for each estimate.

Table 3. Inclusion and Exclusion Criteria Applied to Individual Study Inclusion

Inclusion Criteria	Exclusion Criteria
Include if it has comparator values for either (i) antibiotic susceptible or (ii) no infection cases.	Exclude if has no measure of spread of data and/or statistical significance
Infections within the hospital setting	Exclude if only pre-infection or pre-infection costs reported
If the data were not readily available from the systematic literature review results table, the corresponding individual papers were reviewed (if available), only English language papers were subsequently included if this review step was needed.	Exclude if only antibiotic cost measured
	Post-initial extraction, additionally those who utilise just "colonised" as the exposure "syndrome" were excluded from the analysis

D. Stepwise and Sampling Calculations Performed to Estimate Hospital Cost per Case

To estimate hospital bed-day costs from hospital length of stay (LoS) estimates, hospital costs estimated through WHO-CHOICE at the country level were used. Mean and standard deviation values were therefore extracted from primary-level hospitals for each country[19]. Primary-level hospital values (which cover district and general hospitals) were chosen; these provided the lowest cost estimates. If data were available on the distribution across primary, secondary, and tertiary hospitals these estimates could be individually sampled and a weighted average could be used, this was beyond the scope of this global analysis. To sample these, a lognormal distribution was used, with shape and scale parameters calculated based on the extract data and method of moments[20]. To sample LoS and cost estimates generated from the meta-analyses, which used the "meta" R package[21], a normal distribution was used, given the meta-analyses outputs of mean and standard errors (additionally, negative values meant lognormal and gamma distributions were less suitable). Figure 1 provides an overview of how these were combined. This process was performed for both associated and attributable antibiotic resistance (ABR) effects separately.

Figure 1. Overview of Evidence Synthesis and Modelling Summary for Hospital Cost per Case

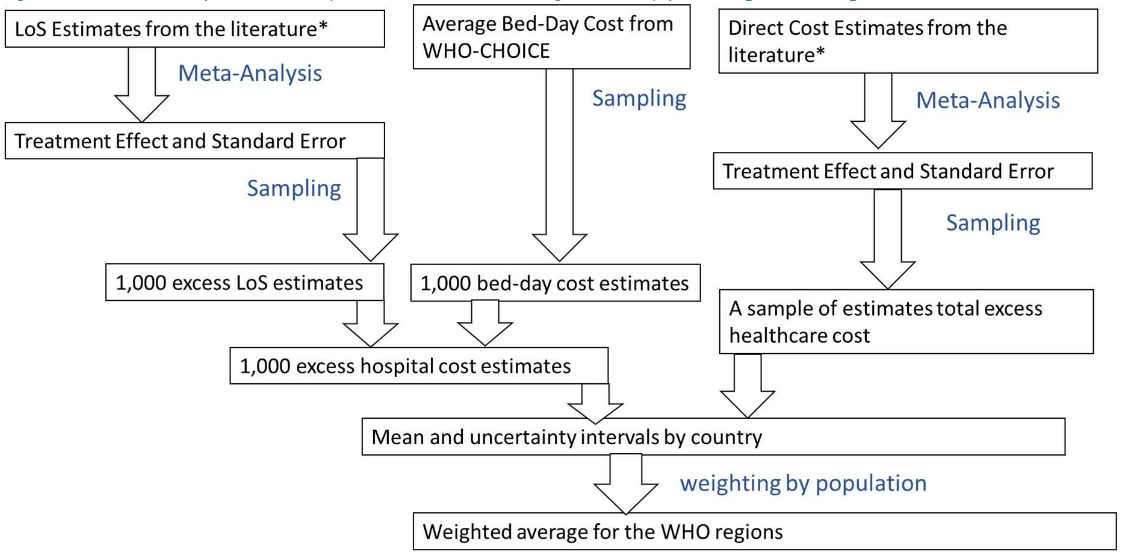

**By regional classification, per drug-bug-syndrome combination*

S3. Estimating Antibiotic Costs and Productivity Costs

A. Additional information on antibiotic cost estimation

Antibiotics listed were matched using the Jaro-Winkler matching method in R to account for potential spelling differences, followed by hand-checks[22,23]. If costs were available through the latter sources, these were used. If not, the median differential factor between the supplier median costs and generic medicine cost estimates, for that country, was applied to the supplier median cost estimate. This was performed to account for the International Medical Products Price Guide being a low-cost estimate.

As antibiotic costs were estimated using fuzzy joining to match antibiotic names across two databases, with set limits and manual checking of matches, some matches may have been lost during the data manipulation stage. However, some quality assurance within the process was utilised and this approach is likely to provide increased inclusion in comparison to methods relying on perfect matches provided through the software. It was limited to antibiotics listed in the 2019 AWARE list[23]. For the matching of injectable drugs, only 1 drug matched dose and formulation (Clindamycin) of suspension products was not matched to vial/ampoule equivalents in MSH[24,25]. Additionally, solid oral dosage costs were mapped to tablet-capsule costs in the absence of specified tablet or capsules from *Hill et al*.[26] Due to the differences in the way data were formatted across sources, automatic data manipulation often wasn't feasible leading to a reliance on manual checking and manipulation. This highlights the importance of internationally defined and utilised data dictionaries for antibiotic naming, dosing, formulation categorization to enable future analyses. Applying UK, India and South Africa adjustments to other countries in respective income groups is highly flawed due to heterogeneous importing/exporting industries for pharmaceuticals and varied healthcare system financing models across countries, however similar processes have been used in previous regional analyses[27], and estimates are converted back to local values and inflated in

line with local GDP growth, and are presented alongside alternative estimates based on more generic cost estimates.

B. Additional information on productivity cost estimation

For each country (after converting wages into 2019 USD), the following process was used to estimate the 2019 wage where (i) 2019 wage data were not available and (ii) previous years (with more than 1 year) wage data were available. A data table for each country where "wage" and "year" were variables was created to estimate average growth rates in wage using the following formulae:

$$Difference\ in\ Time\ =\ year_x - lag(year_x)$$

Where $lag(year_x)$ calls the previous "year" value available for each country at time point x. A similar process was done to calculate the corresponding difference in wage in that country at time point x:

$$Difference\ in\ Wage =\ 1 + (\frac{wage_x - lag(wage_x)}{lag(wage_x)})$$

There were potentially different lengths of time in between reporting, for example a country may have data for 2009, 2010, 2015 and 2016. We therefore need to estimate the average annual growth across these different time points, we do this using the following formulae:

$$Annual\ Growth\ in\ Wage = `Difference\ in\ Wage`\ \hat{}\ (\frac{1}{Difference\ in\ Time})$$

$$Mean\ average\ growth\ in\ wages =\ mean\ (`Annual\ Growth\ in\ Wage`)$$

We then used the latest year available ($year_M$) and compared this to 2019 to calculate how many years of growth were needed to adjust $year_M$ to 2019 values (Y):

$$Y =\ 2019\ -\ year_M$$

$$Last\ Available\ Wage\ =\ wage_{year_M}$$

Then estimated 2019 wages for that country using:

$$Wage_{2019} = `Last\ Available\ Wage`\ *\ (`Mean\ average\ growth\ in\ wages`^{Y})$$

The same process was used for employment rates, capping employment rates at 99.9%. For employment rates and wages, rates in the top and bottom 2.5% percentiles were hand checked, subsequently thresholds of over 10% for employment to population ratios and $10 dollars for nominal monthly wage were set accordingly, with estimates out of these bounds assumed to be data errors. "EAR_4MTH_SEX_ECO_CUR_NB_A" and "EMP_DWAP_SEX_AGE_RT_A" were the indicators used for wage and employment-to-population rations respectively from the "Rilostat" package[28].

Additionally, to note that PA.NUS.PPP", "PA.NUS.FCRF" and "NY.GDP.DEFL.ZS" and "NY.GDP.DEFL.KD.ZG" indicators were utilised from the "wbstats" package for the inflation calculations throughout this study.[11]

S4. Adjustment Factors for length-of-stay based costs

There were 66 extractions that had both costs and length of stay impact estimates that could be used in the cost per bed day adjustment factor across attributable- and associated-ABR studies on hospital cost/LoS impact. Most of these studies were based in High Income countries (see Table 4). As only a few studies were in middle-income studies, cost adjustment factors were not further split into other groupings, e.g., by syndrome. There were no low-income-based data extracted, therefore the lower-middle income adjustment factor was used in practice for low-income countries. Because of the skewedness of cost-adjustment factors in upper-middle income countries and outliers present in high income country data, using the median cost-adjustment factor was more appropriate than the mean for Scenario 2.

All adjustment factors were found to be between 2 and 3 times more. For example, if the mean estimated cost of an infection in an upper middle-income country, based on LOS, was $250, applying the adjustment factor would translate this into $250 + ($250*2.691) = $922.75.

Table 4. Cost Adjustment Factors for Length-of-stay based Excess Cost Estimates

**Income Region**	**Median Adjustment Factor Multiplier (%, rounded to 3 decimal places)**	**Number of Studies used to estimate adjustment factor**
High income	1.870792	54
Upper middle income	2.690948	11
Lower middle income	2.686232	1
Low income	*[Not Available]*	0

S5. Methods for Combining Unit Costs and Epidemiological Estimates

A. Vaccine Scenarios

The vaccine scenarios in Table 5 were taken from a previously published epidemiology analysis[29].

Table 5. Vaccine Scenarios included in this study

**Pathogen**	**Efficacy**	**Coverage**	**Duration**	**Target Disease**	**Target population**
Acinetobacter baumannii	0.7	0.7	5 years	All	All age groups
E. coli - non-diarrheagenic	0.7	0.7	5 years	All except Diarrhoea	All age groups
Enterococcus faecium	0.7	0.7	5 years	All	All age groups
ETEC	0.6	0.7	5 years	Diarrhoea	6 months
Group A streptococcus	0.7	0.7	5 years	All	6 weeks
Klebsiella pneumoniae – all	0.7	0.7	5 years	All	All age groups
Mycobacterium tuberculosis - Improved	0.8	0.7	10 years	All	0 weeks + boost every 10 years
Non-typhoidal Salmonella	0.8	0.7	5 years	All	6 weeks & 9 months
Pseudomonas aeruginosa	0.7	0.7	5 years	BSI, LRI and thorax infections	All age groups
Salmonella paratyphi	0.7	0.7	5 years	All	9 months
Salmonella Typhi	0.85	0.7	15 years	All	9 months
Shigella	0.6	0.7	5 years	All	6 months
Staphylococcus aureus	0.6	0.7	5 years	All	All age groups
Streptococcus pneumoniae – Improved	0.7 (0.5 for RTI)	0.9	5 years	BSI, CNS infections, Cardiac infections, LRI	6 weeks & elderly age group
Haemophilus influenzae type B	59; 92; 93† (69 for LRI)	0.9	5 years	All	6, 10, 14 weeks

†Efficacy corresponding to first, second and third doses respectively.

B. Expert elicitation exercise performed by WHO

Seven pathogens, *Chlamydia trachomatis, Vibrio cholerae, Clostridium difficile, Heliobacter pylori, Group A Streptococcus, Haemophilus influenzae b (Hib),* and *Neisseria meningitidis,* were identified as not having much/any publicly available or published data to be included in the economic evaluation by authors. An expert elicitation survey was conducted by the WHO to estimate the potential vaccine-avertible burden of these pathogens, the results of this survey then fed into this study. For the first four listed pathogens, the WHO aimed to collect both health and economic burden estimates, whilst aiming to collect only economic burden estimates for the latter three pathogens. Methods were based on Bojke et al. (2021)[30] , aiming for consistent yet flexible expert input, allow for individual uncertainty, recognise bias and specialist questions that are suitable for experts alone.

To follow these suggestions, the WHO conducted an online survey that could be sent out virtually to experts globally, using *Typeform*. Experts could then choose which pathogens they wished to answer for, with all

information about the project being presented at the beginning of the survey. For each pathogen, background information was provided to aid experts in their decisions and account for consistency between answers. The background information provided included vaccination target population, disease burden (deaths and DALYs per 100,000), antimicrobial resistance threat (evidence of multi- and pan-drug resistance, past 10-year antimicrobial resistance trend) and economic burden (usual treatment, treatability, treatment cost and hospital length of stay in days). Two health and two economic burden questions were asked per relevant pathogen (see table below). For each pathogen, experts were asked to select an economy, country or WHO region for which they were answering, provided with relevant information such as syndromes or antibiotics, and allowed to skip a question if it was out of their expertise.

Table 6. WHO Survey Questions

Section	Pathogens	Questions	Possible answers:
Health Burden	*Chlamydia trachomatis, Vibrio cholerae, Clostridium difficile, Heliobacter pylori*	Q1: What proportion of patients infected with [*pathogen*] are hospitalised?	0-5%;6-20%;21-50%;51-70%;71-100%
		Q2: What proportion of hospitalised patients are infected with a pathogen resistant to first-line therapy?	0-1%;1-5%;6-20%;21-50%;51-70%;71-100%
Economic Burden	*Chlamydia trachomatis, Vibrio cholerae, Clostridium difficile, Heliobacter pylori, Group A Streptococcus, Haemophilus influenzae b (Hib),* and *Neisseria meningitidis*	Q1: What is the increase in cost for patients with infections from a resistant vs susceptible pathogen when considering hospital costs such as medication, diagnostics, surgery costs, use of hospital equipment, etc.? (Numerical response)	$0-$350;$351-$2,000;$2,001-$6,000;$6,000-10,000;>$10,000
		Q2: What is the increase in length of stay for patients with infections caused by resistant vs susceptible pathogens in hospital?	0-2 days;3-6 days;7-10 days;11-14 days;>15 days

Multiple choice for answers with predetermined values following Leal et al.'s (2007) findings that providing experts with interval answers produced a more cohesive response with possible distributions[31]. Expert groups in AMR were selected by the WHO for the survey to be sent to. The survey was limited to English, French and Spanish speaking languages. No expert identifiable data (such as name, location or contact information) was shared as part of this modelling study. Any engagement with experts did not involve any personal information and the Ethical Committee of the World Health Organization agreed that the ethical approval was not required.

There were 57 respondents in total who responded across all areas of expertise, pathogens, and regions (see below). Some experts provided references for their answers; all were checked by WHO, and any that did not match the reference were not included in the final output.

The aggregated data is available in Table 7. The distributional information based on responses (Table 8) were the outputs that were used in this modelling study. Because of the small sample size per pathogen, analysis was not disaggregated by region.

Table 7. WHO Survey Aggregated Response Data

Question area for experts	Possible answers (multiple selection allowed)	Maximum number of respondents
Expertise	AMR	37
	Clinical	15
	General practitioner	2
	Microbiology	30
	Economics	2
	Epidemiology	29
	Policy	16
Pathogens	*Chlamydia trachomatis*	20
	Vibrio cholerae	20
	Clostridium difficile	15
	Heliobacter pylori	11
	Group A Streptococcus	20
	Haemophilus influenzae b (Hib)	7
	Neisseria meningitidis	13
WHO regions	AFRO	26
	EMRO	9
	EURO	20
	SEAR	10
	PAMR	12
	WPRO	8
	Global	5

Table 8. Shape and scale parameters of excess length of stay estimated through expert elicitation

	**Gamma distribution**		**Lognormal distribution**	
**Microbe**	**Alpha value**	**Beta value**	**Ln(mean)**	**Ln(variance)**
Clostridium difficile	1.930447	4.079365	NA	NA
Cholera	0.778359	19.59251	NA	NA
Group A Streptococcus	0.963048	11.26631	NA	NA
Haemophilus influenzae	0.816638	4.373333	NA	NA
H. Pylori	1.37336	4.472868	NA	NA
Salmonella Typhi	NA	NA	1.530395	0.1013638
Salmonella Paratyphi	NA	NA	1.530395	0.1013638
Non-typhoidal Salmonella	NA	NA	1.969906	0.2367459

Proportion of cases hospitalised data were also used in this modelling study for gastro-related illnesses. The uniform distribution was chosen. Given that large ranges were similar across pathogens, the largest range which encompassed all estimates was chosen (0.035 – 0.85) and the mean value utilised for gastro-related pathogen hospitalisation proportions (see S5.D).

C. Sampling unit costs from meta-analysis

To utilise the available data to estimate the potential associated ABR unit costs in hospital, the following steps were conducted:

1. 17 unique study-exposure groups of interest had both attributable and associated ABR unit costs, these data were utilised to estimate susceptible infection unit costs of those exposure groups of interest, using the same process ABR unit costs estimates were generated.
2. The associated ABR unit costs, attributable ABR unit costs and susceptible unit costs shape and scale parameters estimated through the meta-analyses and expert elicitation exercises were randomly sampled 1,000 times to create 3 sample sets. For estimates in from the meta-analyses conducted as part of this study, a truncated normal distribution was utilised. The upper limit for attributable unit costs was set to 500 days (the maximum out of the highest values in the ranges extracted from the literature) and the upper limit for the associated unit costs was set to 15x this (as this was the maximum difference found when using data from the literature). The lower limit was set to zero, as it was hypothesised by the authors and experts consulted that drug resistant infections would not have less stay than susceptible or non-infected counter parts. This assumption also excluded data with mean excess LoS

values being below zero (data from 1 study). For estimates from the process outlined in S5B, the distributional information used to create sample sets is in Table 8.

3. These 3 sample sets were then combined by country, syndrome, Gram-stain and antibiotic class.
4. If associated ABR costs were available for that exposure group, these were used, if these were not but both susceptible and attributable ABR unit costs estimates were available these were added together to estimate associated ABR unit costs. If neither of these were available for a particular exposure group, but attributable ABR costs were, adjustment factors were utilised.
5. Adjustment factors were calculated by (i) WHO-choice region and antibiotic class and (ii) WHO-choice region comparing attributable and associated unit costs for those which had both.
6. These adjustment factors were then applied to data where only attributable unit costs was available, with preference given to (i) if available for that antibiotic class.
7. This created a sample of 1,000 associated ABR hospital unit cost estimates of exposure groups of interest.
8. These data were combined with the epidemiological exposure group data. If there was not an exact match for exposure groups across the unit cost and epidemiological data the following average values were used (in order) to fill, with lack of data in (a) meaning you would use (b) and so on until (d):
    a. Same WHO-Choice region, gram stain and class
    b. Same WHO-Choice region, antibiotic class
    c. Same WHO-Choice region, syndrome
    d. Same WHO-Choice region

D. Proportions of cases hospitalised

Values utilised to convert cases to cases hospitalised are displayed in Table 9. The expert elicitation used was the process described in S5B.

Table 9. Proportions of cases hospitalised

Abbreviations; BSI – bloodstream infection, CNS – central nervous system, GRAM – data that was used in the GRAM study [32]. RTI - respiratory tract infection, SSTI – skin and soft tissue infection, UTI – urinary tract infections.

Syndrome	Region	med	Source
Gastro-related illness	AFRO	44.5	Expert elicitation
Bone and Joint; BSI; CNS; Cardiac; Intra-abdominal; non-specified. Typhoid, paratyphoid, and iNTS	AFRO	100	Assumption
RTI	AFRO	9.6	GRAM
SSTI	AFRO	27.5	GRAM
UTI	AFRO	28.3	GRAM
Gastro-related illness	EMRO	44.5	Expert Elicitation
Bone and Joint; BSI; CNS; Cardiac; Intra-abdominal; non-specified.<br>Typhoid, paratyphoid, and iNTS	EMRO	100	Assumption
RTI	EMRO	16.5	GRAM
SSTI	EMRO	38.1	GRAM
UTI	EMRO	53.2	GRAM
Gastro-related illness	EURO	44.5	Expert Elicitation
Bone and Joint; BSI; CNS; Cardiac; Intra-abdominal; non-specified.<br>Typhoid, paratyphoid, and iNTS	EURO	100	Assumption
RTI	EURO	21.8	GRAM
SSTI	EURO	34	GRAM
UTI	EURO	35.5	GRAM
Gastro-related illness	AMRO	44.5	Expert Elicitation
Bone and Joint; BSI; CNS; Cardiac; Intra-abdominal; non-specified. Typhoid, paratyphoid, and iNTS	AMRO	100	Assumption
RTI	AMRO	25.2	GRAM
SSTI	AMRO	32.4	GRAM
UTI	AMRO	27.6	GRAM
Gastro-related illness	SEARO	44.5	Expert Elicitation
Bone and Joint; BSI; CNS; Cardiac; Intra-abdominal; non-specified. Typhoid, paratyphoid, and iNTS	SEARO	100	Assumption
RTI	SEARO	19.3	GRAM
SSTI	SEARO	32.8	GRAM
UTI	SEARO	34.7	GRAM
Gastro-related illness	WPRO	44.5	Expert Elicitation
Bone and Joint; BSI; CNS; Cardiac; Intra-abdominal; non-specified. Typhoid, paratyphoid, and iNTS	WPRO	100	Assumption
RTI	WPRO	19	GRAM
SSTI	WPRO	41.8	GRAM
UTI	WPRO	61.8	GRAM

E. Labour productivity losses calculations

Total labour force productivity burden 2019 USD for a particular WHO region i was estimated using.

$$\sum_{t=20}^{2019+\max(LE_{a,i})} WLYL_{ti} * PVW_{t,i}$$

$PVW_{t,i}$ represents present value of the annual average wage at time $t(2019)$ for region i. These were taken from the unit cost repository created using the steps outlined above (the AMR-UCR) and discounted at 3% beyond 2019 [33,34]. LE_a, i = life expectancy for age at death for region i. Additionally $WLYL_i$ represents working life years lost for region i where, total $WLYL = 65 - A$ over time, for A = age at death and $LE_{a,i}$ = life expectancy for age at death when $A \geq 15$ & $LE_{a,i} \geq 65. For\ A < 15\ \ and/or\ LE_{a,i} < 65.\ WLYL = LE_{a,i} - 15$. This was calculated by combining the cases averted data with the WHO Life table data, by age group[35]. The mid-point with age categories was utilised in calculations.

To estimate the impacts accrued in the year 2019, we assume that cases and deaths are evenly distributed throughout the year. Beyond 2019, working life years lost and wages were combined as detailed in the formulae above.

As an additional scenario analyses the potential maximum working life years lost was calculated by estimating life years lost from deaths, at a 0% discount rate and with no employment rate adjustments. For example, if a person died at 60 years old, this would lead to 5 years of working life years lost in the model.

S6. Additional Cost Results

A. Data retrieval and completeness

The process of where the data came from and how much data were utilised for estimating the excess hospital costs associated and attributable to ABR is outlined in Figure 2; Naylor[13], Founou[14], Zhen[15], Wozniak[16] and Cassini et al 2018 [17] included as sources. Table 10 depicts the spread of this data across syndrome/infection-site and antibiotic classes of interest.

Figure 2. Data retrieval process

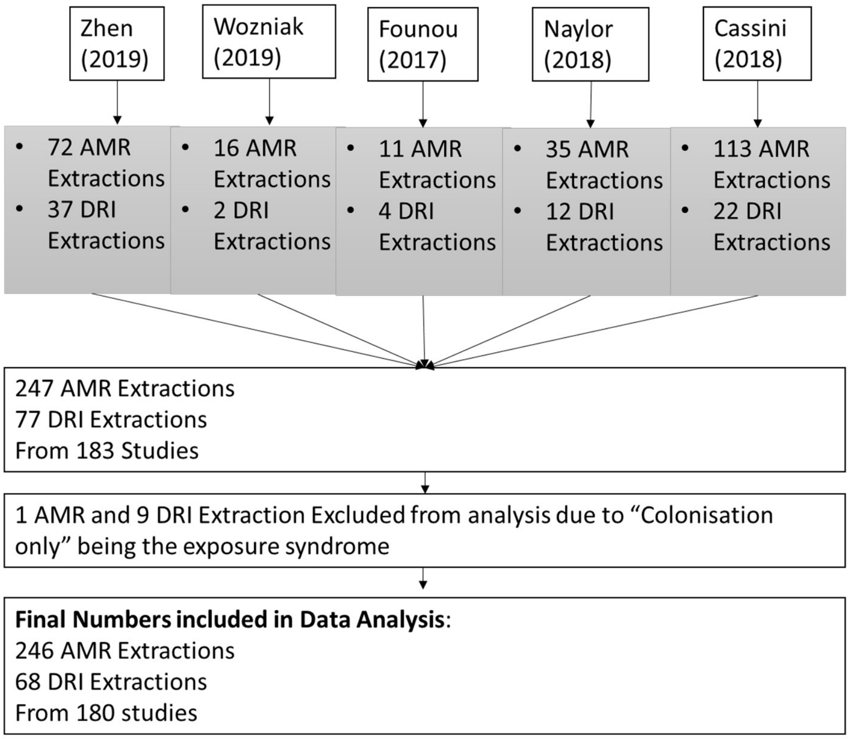

Table 10. Data available by syndrome/infection site and antibiotic class

Abbreviations: ABR – antibiotic resistance, BSI – bloodstream infection, COL/INF – colonization and/or infection, IAI – intra-abdominal infections, INF – infection (non-specified), LoS – length of stay, RTI – respiratory tract infection, SSI – surgical site infection, SSTI – skin and soft tissue infection, UTI – urinary tract infection

syndrome	class	Attributable ABR cost	Associated ABR cost	Attributable ABR LoS	Associated ABR LoS
Bone & joint	penicillins	0	0	1	0
BSI	3g cephalosporins	11	4	37	5
BSI	carbapenems	0	0	13	4
BSI	glycopeptides	5	2	14	3
BSI	penicillins	14	5	31	7
COL/INF	3g cephalosporins	1	0	2	1
COL/INF	carbapenems	5	0	4	5
COL/INF	glycopeptides	0	1	1	1
COL/INF	penicillins	2	0	2	1
IAI	glycopeptides	0	1	0	1
INF	3g cephalosporins	5	2	11	4
INF	carbapenems	5	0	17	4
INF	glycopeptides	4	4	6	2
INF	penicillins	5	4	13	6
Joint	penicillins	0	0	2	0
RTI	3g cephalosporins	0	0	13	0
RTI	carbapenems	1	1	4	1
RTI	MDR	1	0	3	0
RTI	penicillins	5	0	15	0
SSI	penicillins	2	2	2	3
SSTI	penicillins	2	0	3	1
UTI	3g cephalosporins	2	0	4	0
UTI	carbapenems	0	0	2	0
UTI	glycopeptides	0	1	1	0

The number of studies available for different exposure groups, used in the meta-analysis is shown in Figure 3. The mean level of evidence (a composite measure of number of studies that fed into the estimate and from what regional category these studies came from) is displayed in Figure 4. This shows that, on average, for countries in EURO and EMRO regions ABR unit costs associated with blood steam infections (BSIs) and "colonisation and infection with unspecified source" have the highest level of evidence, with more studies from the same WHO-Choice region feeding into their cost per case estimates. For example, there was evidence from 13 studies to inform estimates of costs for 3GC-cephalosporin resistant BSIs for the EURO-A region, but only 1 study to inform glycopeptide resistant urinary tract infection estimates in the SEARO-B region, which was from a country that was not in the same WHO-CHOICE or World Bank (income or geographical) region.

Figure 3. Number of studies reporting hospital cost-per-case data included in the meta-analysis

A. WHO Region

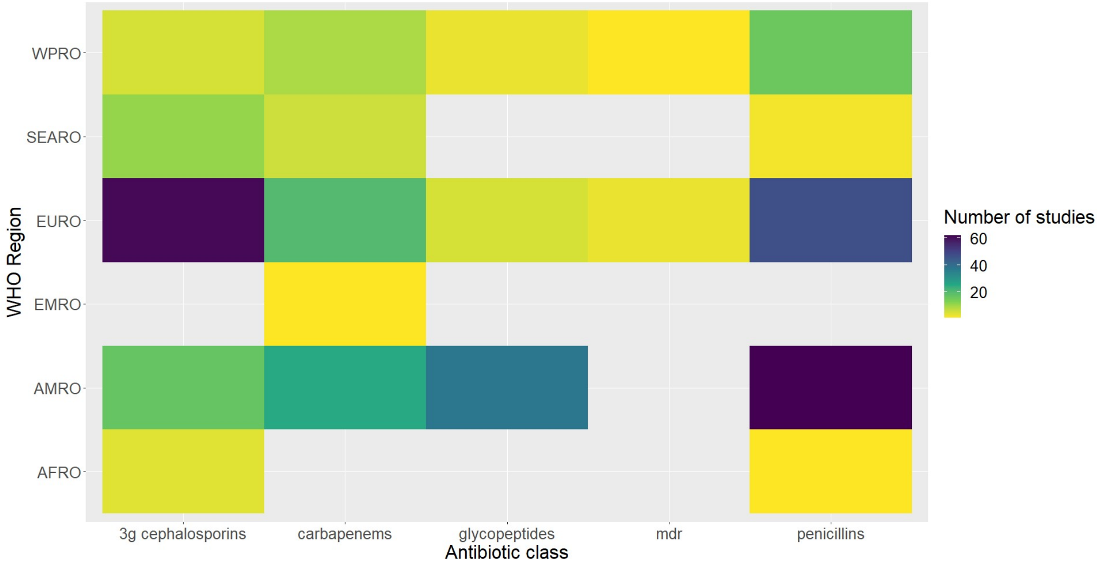

B. World Bank Income group

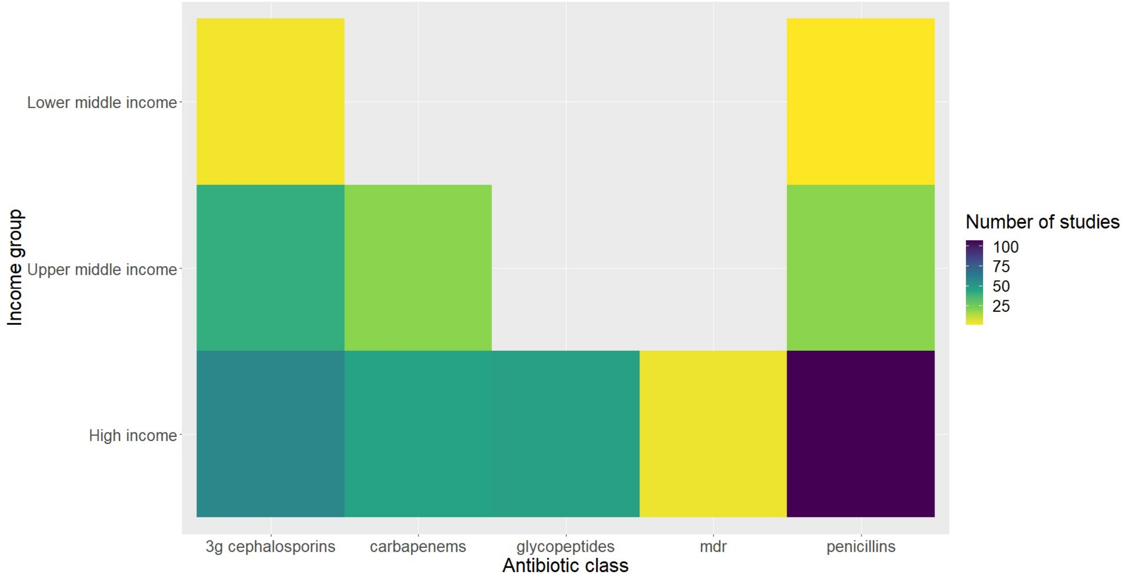

Figure 4. Evidence available for cost per-case estimates by syndrome

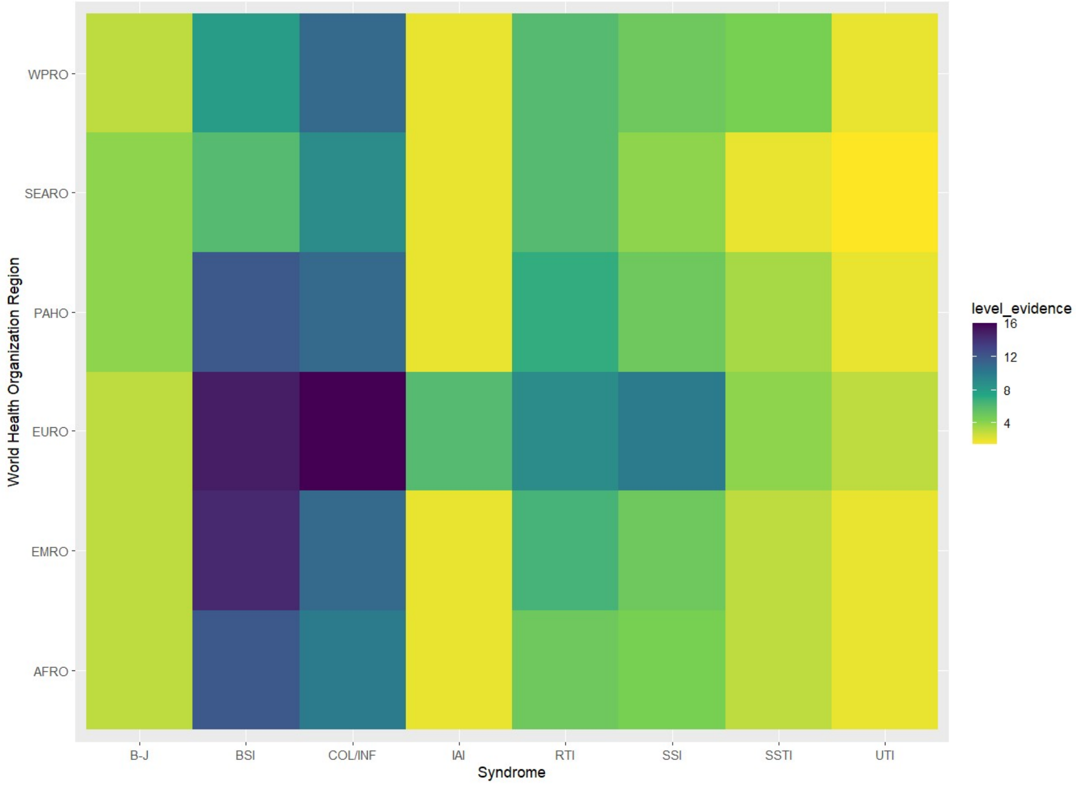

Data completeness for labour productivity estimates can be shown in Figure 5 below. This highlights that there was only complete data on earnings and employment for 37% of the 194 countries, however around 74% had some form of earnings and employment data (from 2019 or previous years).

Figure 5. Evidence available for country productivity estimates

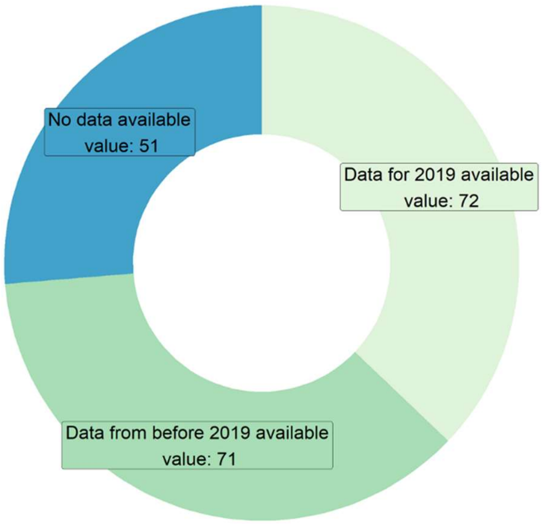

B. Hospital cost per case estimates

We can see countries which have cost-per-case estimates at the lower end of the scale in Figure 6.A (based on both direct cost estimate and LoS estimates converted into monetary costs) provide much higher costs than when compared to Figure 6.C (based on only LoS estimates converted into costs). However, if we adjust LoS-based estimates using an adjustment factor (Figure 6.D, see Supplementary Material S4 for adjustment factors used), this potentially leads to an over-inflation of costs, especially at the upper end of the scale.

Figure 6. Mean Hospital Cost per Case

Average values across significant hospital cost per case across exposure groups, per country. Costs are in USD, presented as a key at the bottom of each map.

Figure 6.A Attributable to ABR based on costs and length of hospital stay estimates.

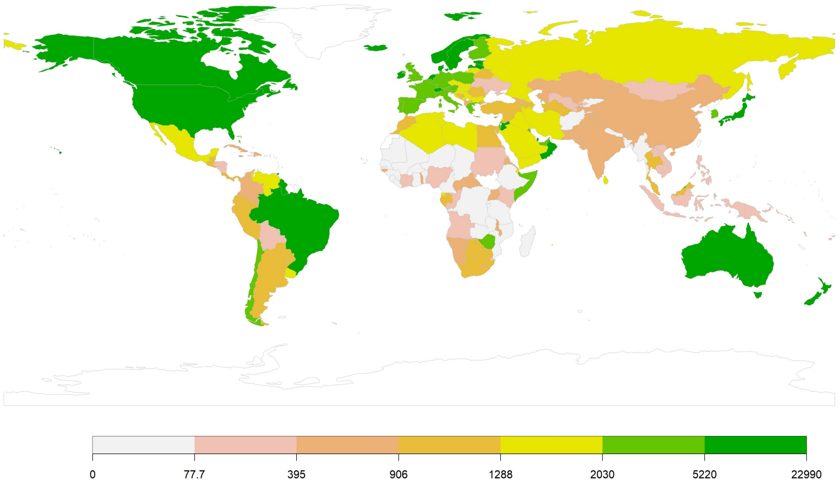

Figure 6.B Associated with ABR based on costs and length of hospital stay estimates.

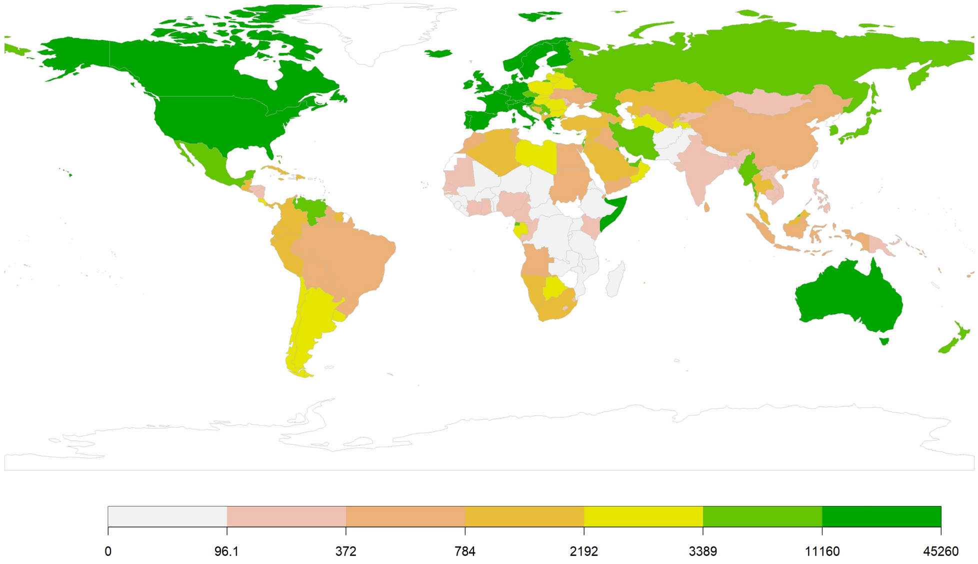

Figure 6.C Attributable to ABR based on length of hospital stay estimates.

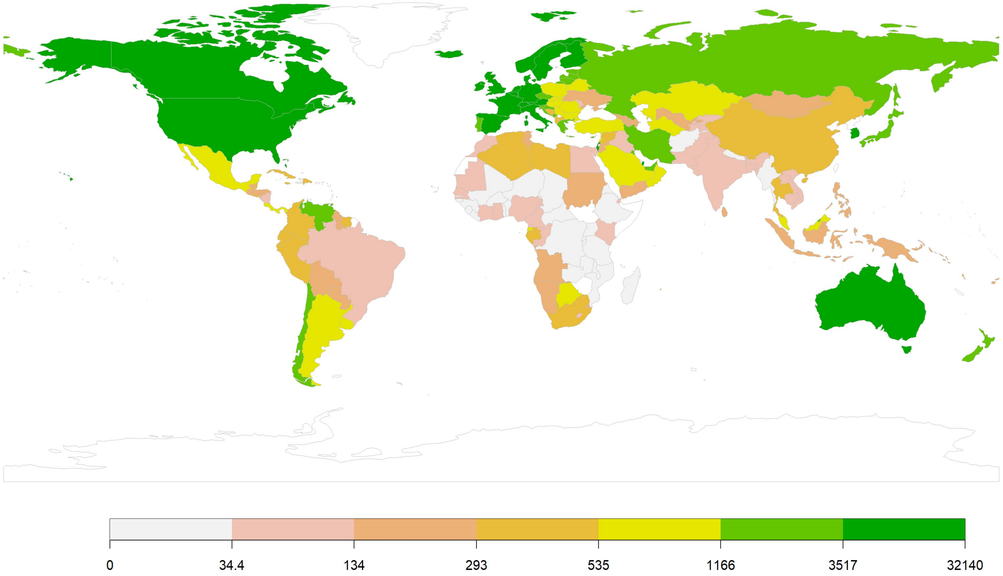

Figure 6.D Attributable to ABR based on costs and length of hospital stay estimates and an adjustment factor.

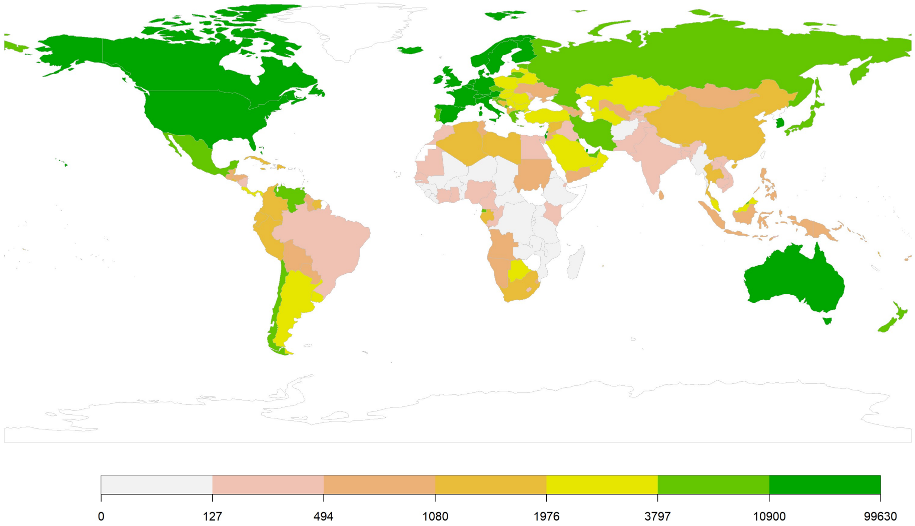

Figure 7 depicts the global values of hospital cost per case, broken down by bacteria and antibiotic class, highlighting the large UIs for such estimates, see below for specific cost estimates by region.

Figure 7. Global average of hospital cost per case, based on length of stay, from the unit cost repository.

Abbreviations: ABR – antibiotic resistance, B-J – bone & joint, BSI – bloodstream infection, COL/INF – colonization and/or infection, IAI – intra-abdominal infections, RTI – respiratory tract infection, SSI – surgical site infection, SSTI – skin and soft tissue infection, UTI – urinary tract infection

A. Attributable to ABR

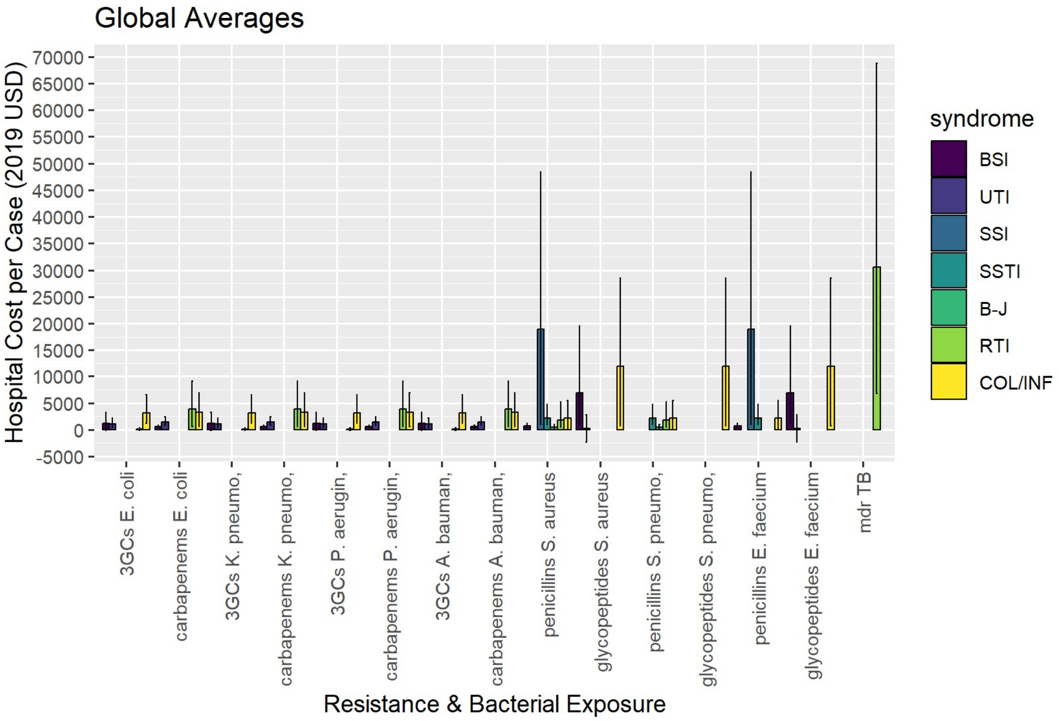

B. Associated with ABR

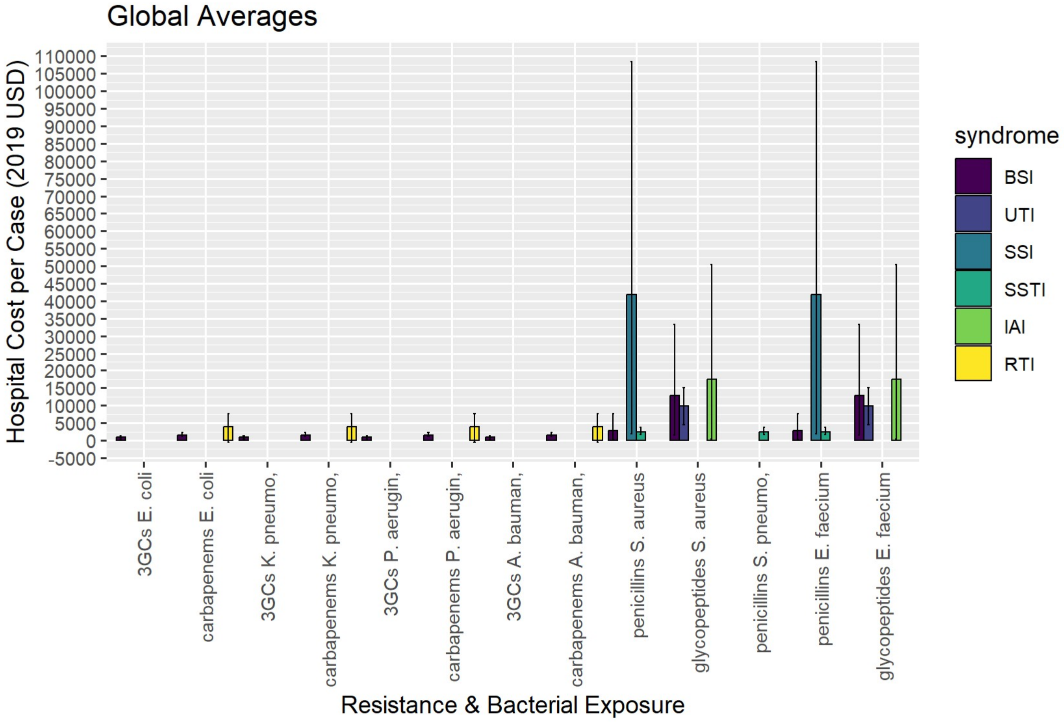

For individual country, antibiotic class and bacteria level estimates please see the online repository: https://github.com/NikkiR08/AMR-UCR/blob/main/cost_per_case/outputs/Results_Table_Bug_Country.csv

Table 11. Regional values for excess hospital cost per case attributable to ABR

WHO region	Syndrome	Gram stain	Antibiotic class	Mean cost across all evidence	low 95% UI across all evidence	High 95% UI across all evidence	Mean cost based on LoS	low 95% UI based on LoS	Low 95% UI across all evidence
AFRO	BSI	Gram-negative	3g cephalosporins	235	166	631	140	8	973
AFRO	BSI	Gram-negative	carbapenems	27	-315	203	26	-316	203
AFRO	BSI	Gram-positive	glycopeptides	1618	1444	2392	232	14	1696
AFRO	BSI	Gram-positive	penicillins	253	126	730	298	32	1313
AFRO	UTI	Gram-negative	3g cephalosporins	884	787	1210	102	6	742
AFRO	UTI	Gram-negative	carbapenems	266	16	2017	266	16	2016
AFRO	UTI	Gram-positive	glycopeptides	81	4	502	80	4	497
AFRO	SSI	Gram-positive	penicillins	19529	18593	20270	217	13	1603
AFRO	SSTI	Gram-positive	penicillins	910	333	3769	328	20	2128
AFRO	COL/INF	Gram-negative	3g cephalosporins	169	61	939	140	7	888
AFRO	COL/INF	Gram-negative	carbapenems	2001	1618	3406	139	11	681
AFRO	COL/INF	Gram-positive	glycopeptides	11514	10821	11767	147	8	1084
AFRO	COL/INF	Gram-positive	penicillins	1673	1533	2097	57	6	207
AFRO	B-J	Gram-positive	penicillins	263	8	1529	262	8	1527
AFRO	RTI	Gram-negative	3g cephalosporins	5	0	52	5	0	52
AFRO	RTI	Gram-negative	carbapenems	3720	3134	6238	793	4	5137
AFRO	RTI	Gram-positive	penicillins	-311	-379	-161	51	4	304
AFRO	RTI	tb	mdr	27584	24417	41930	4243	255	29699
EMRO	BSI	Gram-negative	3g cephalosporins	453	167	1832	602	6	3331
EMRO	BSI	Gram-negative	carbapenems	619	-653	5113	619	-654	5111
EMRO	BSI	Gram-positive	glycopeptides	2055	1460	4612	1098	9	6346
EMRO	BSI	Gram-positive	penicillins	385	112	1475	581	6	2722
EMRO	UTI	Gram-negative	3g cephalosporins	1075	799	2221	481	4	2814
EMRO	UTI	Gram-negative	carbapenems	1168	11	7571	1168	11	7567
EMRO	UTI	Gram-positive	glycopeptides	339	2	1341	336	2	1321
EMRO	SSI	Gram-positive	penicillins	20356	18496	37188	1020	9	5937
EMRO	SSTI	Gram-positive	penicillins	1615	330	4400	1350	14	7262
EMRO	COL/INF	Gram-negative	3g cephalosporins	5019	72	19389	1847	13	12330
EMRO	COL/INF	Gram-negative	carbapenems	4574	1517	13084	939	8	6307
EMRO	COL/INF	Gram-positive	glycopeptides	11943	10986	21276	696	6	4038
EMRO	COL/INF	Gram-positive	penicillins	1871	1478	3247	406	4	2786
EMRO	B-J	Gram-positive	penicillins	476	7	3294	475	7	3291
EMRO	RTI	Gram-negative	3g cephalosporins	-69	-713	145	-69	-714	144
EMRO	RTI	Gram-negative	carbapenems	4086	3170	8933	1389	2	11248
EMRO	RTI	Gram-positive	penicillins	-194	-736	620	294	2	1880
EMRO	RTI	tb	mdr	35055	24934	66916	19789	559	86364
EURO	BSI	Gram-negative	3g cephalosporins	1489	148	5477	1967	29	9235
EURO	BSI	Gram-negative	carbapenems	3019	-432	15026	3017	-433	15019
EURO	BSI	Gram-positive	glycopeptides	20087	2128	34863	6767	112	32785
EURO	BSI	Gram-positive	penicillins	1151	152	3550	1294	14	5273
EURO	UTI	Gram-negative	3g cephalosporins	1617	702	5031	1829	30	8683
EURO	UTI	Gram-negative	carbapenems	2938	74	13013	2936	74	13004
EURO	UTI	Gram-positive	glycopeptides	1270	20	7336	1260	20	7286
EURO	SSI	Gram-positive	penicillins	20662	18678	27157	3425	56	16130
EURO	SSTI	Gram-positive	penicillins	3548	265	13327	4259	67	19514
EURO	COL/INF	Gram-negative	3g cephalosporins	12126	92	30174	6939	50	33335
EURO	COL/INF	Gram-negative	carbapenems	8167	1627	16331	2906	41	13258
EURO	COL/INF	Gram-positive	glycopeptides	18139	11560	28434	4818	83	24202
EURO	COL/INF	Gram-positive	penicillins	6977	1926	16943	4484	27	23315
EURO	B-J	Gram-positive	penicillins	634	45	2775	632	45	2765
EURO	RTI	Gram-negative	3g cephalosporins	-380	-1844	92	-381	-1847	92

EURO	RTI	Gram-negative	carbapenems	5096	3303	10071	3722	46	13572
EURO	RTI	Gram-positive	penicillins	17717	-177	30766	5045	36	26351
EURO	RTI	tb	mdr	44895	26242	88556	40751	2719	126728
PAHO	BSI	Gram-negative	3g cephalosporins	1941	209	15165	1419	100	5700
PAHO	BSI	Gram-negative	carbapenems	156	-182	2223	155	-183	2222
PAHO	BSI	Gram-positive	glycopeptides	2089	1514	4444	1257	105	6211
PAHO	BSI	Gram-positive	penicillins	614	122	3725	667	68	2751
PAHO	UTI	Gram-negative	3g cephalosporins	1127	830	2458	458	35	1724
PAHO	UTI	Gram-negative	carbapenems	1799	97	13153	1798	96	13147
PAHO	UTI	Gram-positive	glycopeptides	488	16	2793	484	15	2777
PAHO	SSI	Gram-positive	penicillins	19596	18617	21563	1171	98	5858
PAHO	SSTI	Gram-positive	penicillins	2547	393	4362	1561	136	7396
PAHO	COL/INF	Gram-negative	3g cephalosporins	2682	102	17870	1648	54	8733
PAHO	COL/INF	Gram-negative	carbapenems	2314	1216	12975	700	30	6279
PAHO	COL/INF	Gram-positive	glycopeptides	11410	10715	12156	720	66	2884
PAHO	COL/INF	Gram-positive	penicillins	1559	275	1836	150	13	701
PAHO	B-J	Gram-positive	penicillins	1418	44	5445	1416	44	5439
PAHO	RTI	Gram-negative	3g cephalosporins	-73	-711	60	-74	-712	59
PAHO	RTI	Gram-negative	carbapenems	4392	3158	7078	2319	22	7880
PAHO	RTI	Gram-positive	penicillins	-189	-353	245	271	20	1218
PAHO	RTI	tb	mdr	36544	25416	82019	23251	2178	113469
SEARO	BSI	Gram-negative	3g cephalosporins	422	180	861	178	22	479
SEARO	BSI	Gram-negative	carbapenems	-132	-955	455	-132	-956	455
SEARO	BSI	Gram-positive	glycopeptides	7037	1490	22423	192	12	627
SEARO	BSI	Gram-positive	penicillins	422	122	1230	168	24	449
SEARO	UTI	Gram-negative	3g cephalosporins	938	827	1555	104	12	273
SEARO	UTI	Gram-negative	carbapenems	264	26	740	264	26	740
SEARO	UTI	Gram-positive	glycopeptides	82	10	255	81	10	254
SEARO	SSI	Gram-positive	penicillins	20834	18557	38379	205	20	575
SEARO	SSTI	Gram-positive	penicillins	2399	344	5690	342	50	920
SEARO	COL/INF	Gram-negative	3g cephalosporins	501	65	1181	187	10	705
SEARO	COL/INF	Gram-negative	carbapenems	1976	1552	3750	128	14	506
SEARO	COL/INF	Gram-positive	glycopeptides	12241	10880	22678	163	23	428
SEARO	COL/INF	Gram-positive	penicillins	1784	1528	3107	74	15	251
SEARO	B-J	Gram-positive	penicillins	213	16	680	213	16	679
SEARO	RTI	Gram-negative	3g cephalosporins	43	1	192	43	1	192
SEARO	RTI	Gram-negative	carbapenems	3886	3151	6230	811	10	3491
SEARO	RTI	Gram-positive	penicillins	-377	-756	-258	3	-155	166
SEARO	RTI	tb	mdr	28162	23723	43792	2903	52	10977
WPRO	BSI	Gram-negative	3g cephalosporins	780	261	2050	1010	85	3774
WPRO	BSI	Gram-negative	carbapenems	1102	52	5778	1102	52	5776
WPRO	BSI	Gram-positive	glycopeptides	11403	1746	13701	728	22	4228
WPRO	BSI	Gram-positive	penicillins	1147	682	3644	1194	152	5931
WPRO	UTI	Gram-negative	3g cephalosporins	1184	830	2716	735	39	3874
WPRO	UTI	Gram-negative	carbapenems	1693	84	8822	1692	84	8817
WPRO	UTI	Gram-positive	glycopeptides	540	12	3324	536	12	3303
WPRO	SSI	Gram-positive	penicillins	19503	18050	21795	1323	69	6571
WPRO	SSTI	Gram-positive	penicillins	3489	2072	6509	2117	163	8548
WPRO	COL/INF	Gram-negative	3g cephalosporins	5295	78	20414	2559	34	14024
WPRO	COL/INF	Gram-negative	carbapenems	4118	1847	13611	1276	49	7197
WPRO	COL/INF	Gram-positive	glycopeptides	11693	10949	13278	969	77	4336
WPRO	COL/INF	Gram-positive	penicillins	2116	1655	3674	1017	82	4027
WPRO	B-J	Gram-positive	penicillins	327	55	1044	326	55	1043
WPRO	RTI	Gram-negative	3g cephalosporins	472	-767	4429	471	-768	4427
WPRO	RTI	Gram-negative	carbapenems	3724	3153	5228	895	31	4086
WPRO	RTI	Gram-positive	penicillins	-78	-389	1062	505	7	2811
WPRO	RTI	tb	mdr	28070	23809	48482	5891	126	48000

Table 12. Regional values for excess hospital cost per case associated with ABR

WHO region	syndrome	Gram stain	Antibiotic class	Mean cost across all evidence	Low 95% UI across all evidence	High 95% UI across all evidence	Mean cost based on LoS	Low 95% UI based on LoS	High 95% UI across all evidence
AFRO	BSI	Gram-negative	3g cephalosporins	426	312	1133	225	13	1645
AFRO	BSI	Gram-negative	carbapenems	680	17	3824	680	17	3822
AFRO	BSI	Gram-positive	glycopeptides	12913	11956	14521	396	23	2881
AFRO	BSI	Gram-positive	penicillins	690	517	1682	326	20	2360
AFRO	UTI	Gram-positive	glycopeptides	10320	9786	10813	NA	NA	NA
AFRO	SSI	Gram-positive	penicillins	44490	42107	46187	496	30	3634
AFRO	SSTI	Gram-positive	penicillins	535	32	3889	534	32	3887
AFRO	COL/INF	Gram-negative	3g cephalosporins	1127	916	1941	389	16	2062
AFRO	COL/INF	Gram-negative	carbapenems	241	17	1393	241	17	1392
AFRO	COL/INF	Gram-positive	glycopeptides	7120	6634	8147	303	18	2211
AFRO	COL/INF	Gram-positive	penicillins	17530	16311	19412	466	27	3431
AFRO	IAI	Gram-positive	glycopeptides	18424	16919	19362	74	4	528
AFRO	RTI	Gram-negative	carbapenems	3011	2588	5123	624	34	4748
EMRO	BSI	Gram-negative	3g cephalosporins	834	314	3473	1066	9	6240
EMRO	BSI	Gram-negative	carbapenems	1766	-59	8180	1765	-60	8176
EMRO	BSI	Gram-positive	glycopeptides	13829	12013	24705	1881	16	10988
EMRO	BSI	Gram-positive	penicillins	1296	524	5123	1571	14	9226
EMRO	UTI	Gram-positive	glycopeptides	10307	9416	10972	NA	NA	NA
EMRO	SSI	Gram-positive	penicillins	46259	42782	84732	2337	20	13517
EMRO	SSTI	Gram-positive	penicillins	2548	22	14904	2546	22	14896
EMRO	COL/INF	Gram-negative	3g cephalosporins	1289	918	3194	700	13	4503
EMRO	COL/INF	Gram-negative	carbapenems	902	12	5215	902	12	5213
EMRO	COL/INF	Gram-positive	glycopeptides	7821	6702	13393	1440	13	8358
EMRO	COL/INF	Gram-positive	penicillins	18669	16504	32474	2202	19	12762
EMRO	IAI	Gram-positive	glycopeptides	19087	16994	34343	348	3	2039
EMRO	RTI	Gram-negative	carbapenems	4208	2666	12278	3022	23	18810
EURO	BSI	Gram-negative	3g cephalosporins	2421	337	10458	4176	58	20031
EURO	BSI	Gram-negative	carbapenems	5128	103	21669	5125	103	21654
EURO	BSI	Gram-positive	glycopeptides	15486	12086	26562	6295	102	29052
EURO	BSI	Gram-positive	penicillins	4672	560	21138	8208	86	40597
EURO	UTI	Gram-positive	glycopeptides	10381	9731	10957	NA	NA	NA
EURO	SSI	Gram-positive	penicillins	45566	41448	55003	5231	81	23935
EURO	SSTI	Gram-positive	penicillins	8481	138	39479	8477	138	39462
EURO	COL/INF	Gram-negative	3g cephalosporins	1390	981	2637	950	58	3332
EURO	COL/INF	Gram-negative	carbapenems	3744	66	17601	3741	66	17586
EURO	COL/INF	Gram-positive	glycopeptides	9217	6777	18406	4860	77	22853
EURO	COL/INF	Gram-positive	penicillins	18922	16751	27066	4366	67	19901
EURO	IAI	Gram-positive	glycopeptides	18778	17442	21214	1176	18	5477
EURO	RTI	Gram-negative	carbapenems	7554	2727	25551	9648	169	44357
PAHO	BSI	Gram-negative	3g cephalosporins	921	340	3629	1217	78	6558
PAHO	BSI	Gram-negative	carbapenems	2540	101	7631	2539	101	7627
PAHO	BSI	Gram-positive	glycopeptides	13475	12217	17081	2149	181	10745
PAHO	BSI	Gram-positive	penicillins	1407	589	5097	1790	152	9062
PAHO	UTI	Gram-positive	glycopeptides	10362	9833	10807	NA	NA	NA
PAHO	SSI	Gram-positive	penicillins	44604	42543	50584	2778	224	14929
PAHO	SSTI	Gram-positive	penicillins	2916	212	15164	2914	212	15158
PAHO	COL/INF	Gram-negative	3g cephalosporins	1405	967	2417	973	85	3005
PAHO	COL/INF	Gram-negative	carbapenems	2077	77	17286	2076	77	17276
PAHO	COL/INF	Gram-positive	glycopeptides	7637	6793	11023	1652	141	8243
PAHO	COL/INF	Gram-positive	penicillins	18134	16690	24070	2670	210	14932
PAHO	IAI	Gram-positive	glycopeptides	18315	17113	19513	403	34	2017
PAHO	RTI	Gram-negative	carbapenems	4302	2694	11787	3392	218	18055
SEARO	BSI	Gram-negative	3g cephalosporins	434	334	609	213	21	602
SEARO	BSI	Gram-negative	carbapenems	666	40	2473	666	40	2472
SEARO	BSI	Gram-positive	glycopeptides	13519	11975	23818	375	37	1063
SEARO	BSI	Gram-positive	penicillins	3211	542	11590	311	34	893
SEARO	UTI	Gram-positive	glycopeptides	10267	9826	10976	NA	NA	NA
SEARO	SSI	Gram-positive	penicillins	47424	42102	84790	470	47	1328
SEARO	SSTI	Gram-positive	penicillins	508	50	1423	508	50	1423
SEARO	COL/INF	Gram-negative	3g cephalosporins	1137	946	1802	305	21	1308
SEARO	COL/INF	Gram-negative	carbapenems	222	26	761	222	26	761
SEARO	COL/INF	Gram-positive	glycopeptides	7551	6738	13580	291	29	821
SEARO	COL/INF	Gram-positive	penicillins	18487	16551	32657	451	47	1244
SEARO	IAI	Gram-positive	glycopeptides	20098	18014	36144	71	7	198
SEARO	RTI	Gram-negative	carbapenems	3117	2634	4997	600	58	1876

WPRO	BSI	Gram-negative	3g cephalosporins	1002	343	3887	1375	68	7069
WPRO	BSI	Gram-negative	carbapenems	1511	62	8246	1510	62	8242
WPRO	BSI	Gram-positive	glycopeptides	13642	11959	18155	2419	127	11919
WPRO	BSI	Gram-positive	penicillins	6174	3012	8298	2097	116	10080
WPRO	UTI	Gram-positive	glycopeptides	10313	9657	11009	NA	NA	NA
WPRO	SSI	Gram-positive	penicillins	44758	42459	49233	3023	157	14814
WPRO	SSTI	Gram-positive	penicillins	3261	163	16716	3259	163	16709
WPRO	COL/INF	Gram-negative	3g cephalosporins	1169	954	1692	498	68	1507
WPRO	COL/INF	Gram-negative	carbapenems	2136	85	11430	2135	85	11424
WPRO	COL/INF	Gram-positive	glycopeptides	7775	6693	11304	1870	95	9179
WPRO	COL/INF	Gram-positive	penicillins	18455	16558	24736	3029	159	15077
WPRO	IAI	Gram-positive	glycopeptides	18270	17155	19430	453	24	2249
WPRO	RTI	Gram-negative	carbapenems	4579	2722	13434	3810	189	20799

Figure 8. Comparison of approaches for estimating hospital cost per case attributable to and associated with ABR

Note this represents mean values per region for exposure groups that were found to have a significant impact on hospital cost based on the combined sample (based on both LoS and direct cost effect estimates).

A. Attributable to ABR

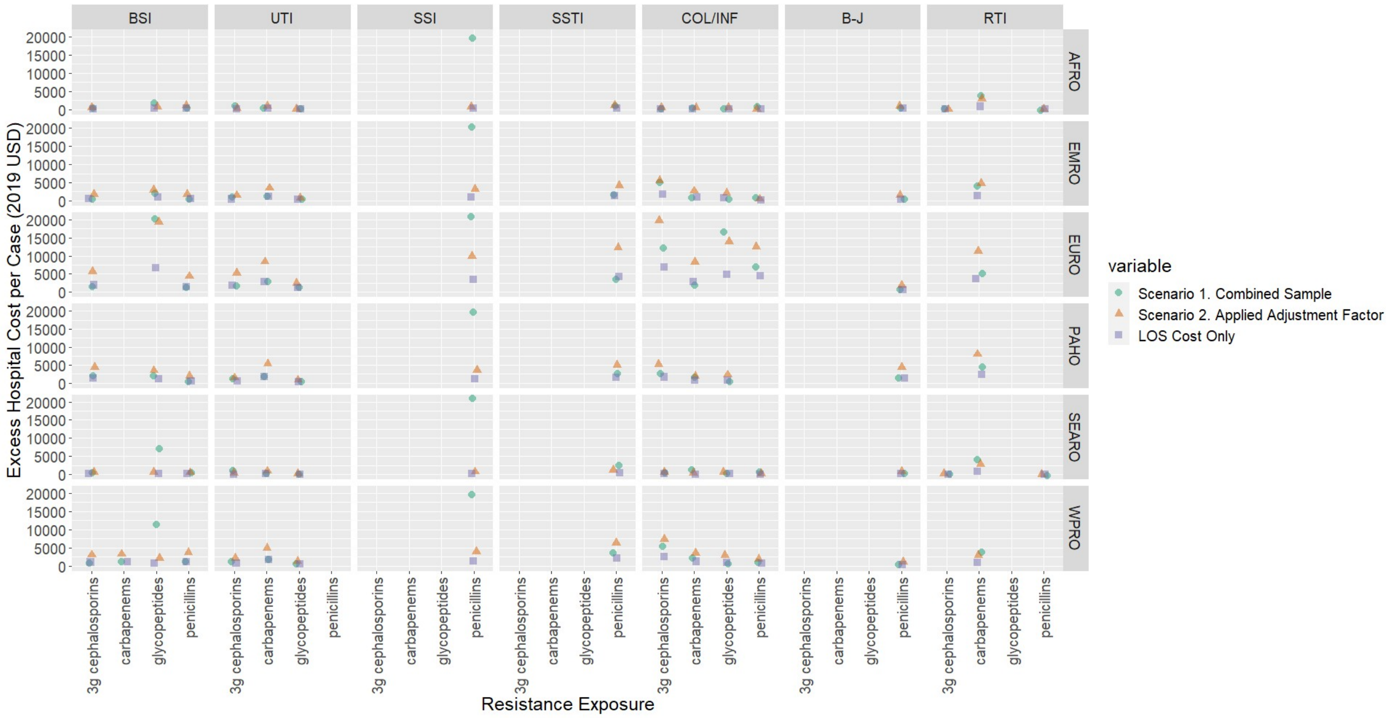

B. Associated with ABR

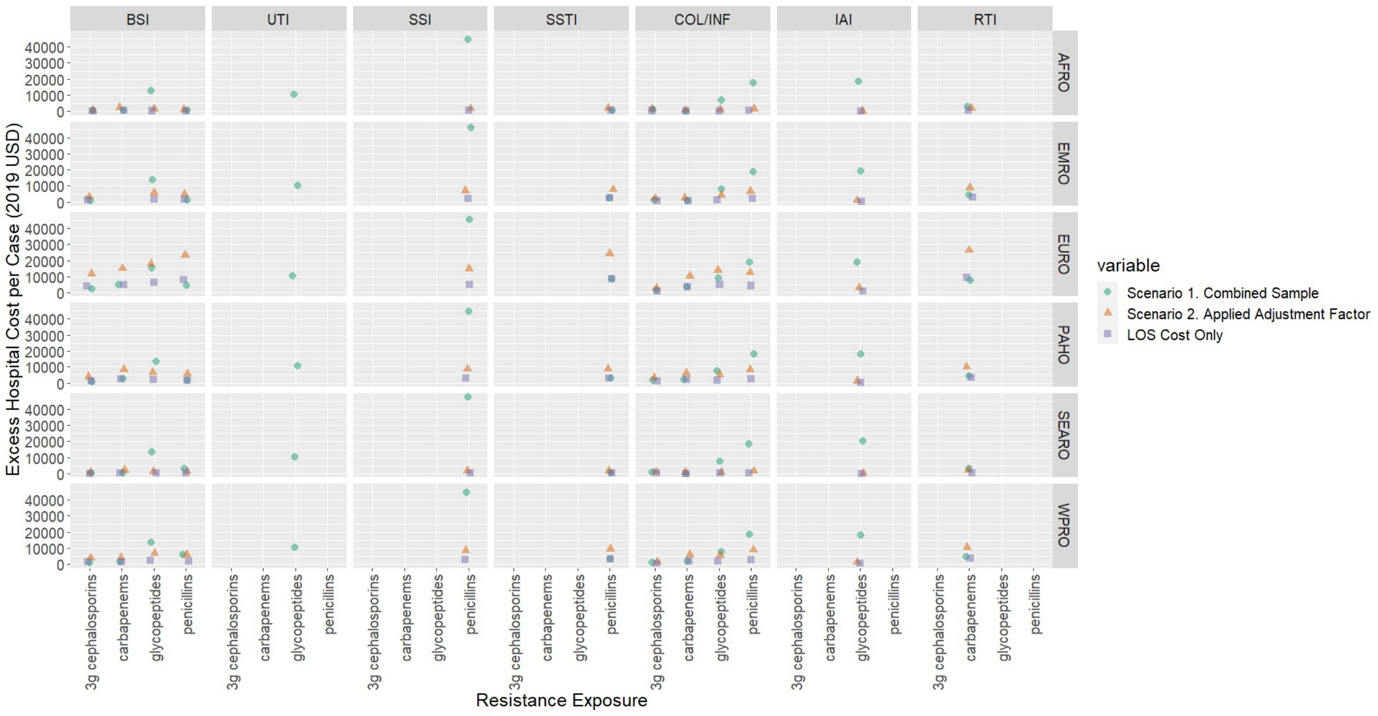

C. Antibiotic unit costs

There were 76 antibiotic formulations with International Medical Products Price Guide MSH supplier costs[24]; 35 oral tablet-capsules and 41 suitable for injection, estimated across 194 countries. This led to 14,678 combinations of estimates for drug-dosage-formulation costs at the country level. 99.6% of these (14,618) of these had an adjusted cost estimated through this study, those that didn't were due to those countries not being listed in the World Bank regional grouping data[7]. There are 17 classes covered, Aminoglycosides, Amphenicols, Aminocyclitols, Beta lactams, Carbapenems, First-generation cephalosporins, Fluoroquinolones, Imidazole, Lincosamides, Macrolides, Nitrofurantoin, Oxazolidinones, Penicillins, Rifamycin, Tetracyclines, Third-generation cephalosporins, Trimethoprim – sulfonamide, Second-generation cephalosporins.

The cost difference seen between the supplier median cost given in the MSH and the generic costs estimated by *Hill et al*[26] and *Gotham et al*[25] showed a much higher differential across injectable formulations (with injectable generic costs being around two times higher than MSH costs) than across oral formulations (with oral generic costs being around 3/4s of the MSH costs comparatively). Individual price estimates will vary on region and country due to inflation and exchange rate differences, see the online repository for the table of regional estimates (https://github.com/NikkiR08/AMR-UCR/blob/main/antibiotic/outputs/regional_abx_cost.csv).

Stratifying by AWARE classifications, the only "Reserve" antibiotic costed in this analysis is linezolid tablet/capsule at a 600 mg dose (utilised to treat pulmonary TB patients). This had, across countries, median (and IQR) values of $0.51 (0.49 - 0.53) to $7.81 (0.48 - 30.64) based on Scenario 1 "Generic" and Scenario 2 "Income" groups respectively. Though total treatment costs will be dependent on the prescribed antibiotic course, for oral 250mg antibiotic doses there was a median cost per antibiotic of $0.03 and $0.06 for Access and Watch antibiotics respectively. From Figure 9 we can see that for oral 250 mg tablet-capsules, "Watch" antibiotics tend to cost more across per antibiotic. However, for 1000 mg vials/injectable antibiotics, "Watch" antibiotics cost more on average utilizing Scenario 1 estimates, but "Access" antibiotics slightly more utilizing Scenario 2 estimates correspondingly. However, it is also important to note that the recommended antibiotic course will have an impact on total costs, as it could be Access antibiotics have longer durations/higher defined daily doses than Watch antibiotics. Though, we do produce through this study an accessible resource for antibiotic stewardship evaluations which incorporate costs.

Outliers seen in Scenario 2 data (see Figure 9) can be partly attributed to countries that experienced substantial GDP growth between 2015 and 2019 that are classified as High income, leading to large inflation of unit costs across that timeline and subsequently a relatively large adjustment factor. This study suggests that using

individual countries as proxy estimates by income group may cause an overestimation at the country and regional level.

Figure 9. Antibiotic Costs for Access and Watch Antibiotics

(a) 250 mg Oral Comparison

This includes amoxicillin, ampicillin, azithromycin, cephalexin, cefuroxime, chloramphenicol, ciprofloxacin, erythromycin, flucloxacillin, levofloxacin and tetracycline hcl.

(i) All data visible

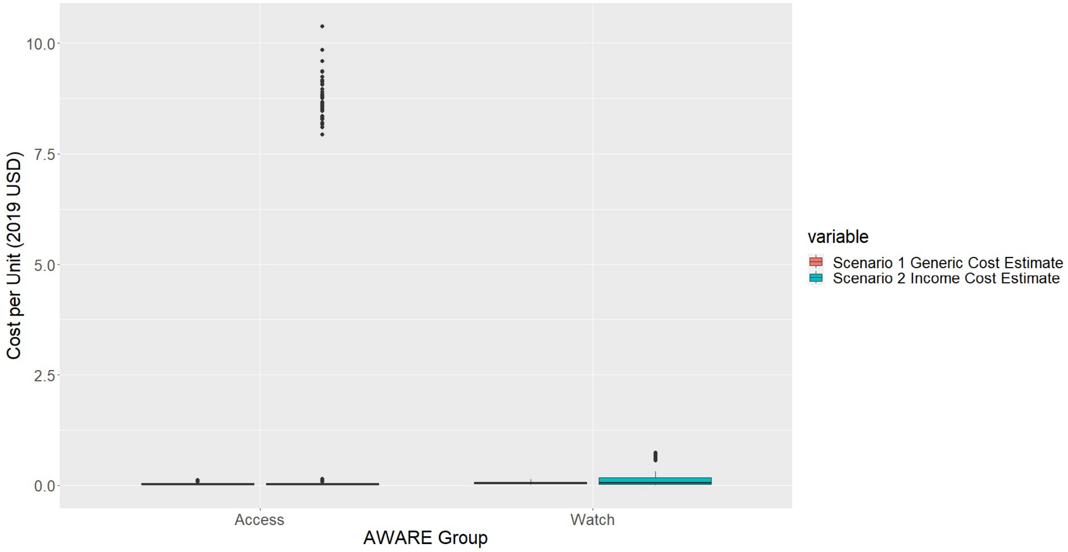

(ii) Zoomed-in with y-axis specified between 0 and 0.5, excluding outliers

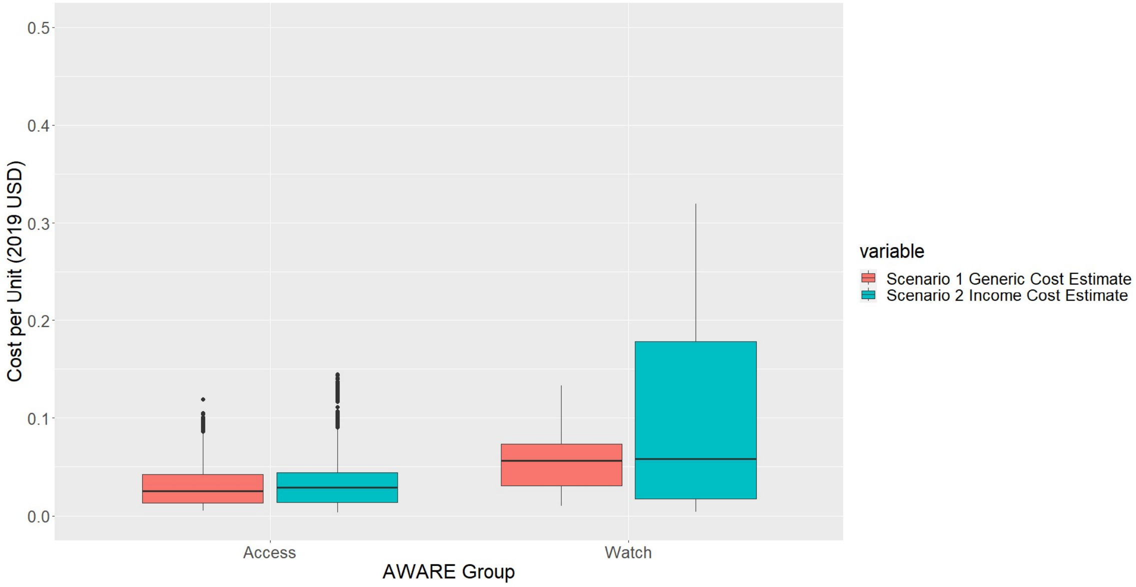

(b) 1000 mg Vial Comparison

This includes ampicillin, cefotaxime, ceftazidime, ceftriaxone, chloramphenicol cloxacillin, kanamycin sulfate, meropenem, streptomycin sulfate

(i) All data visible

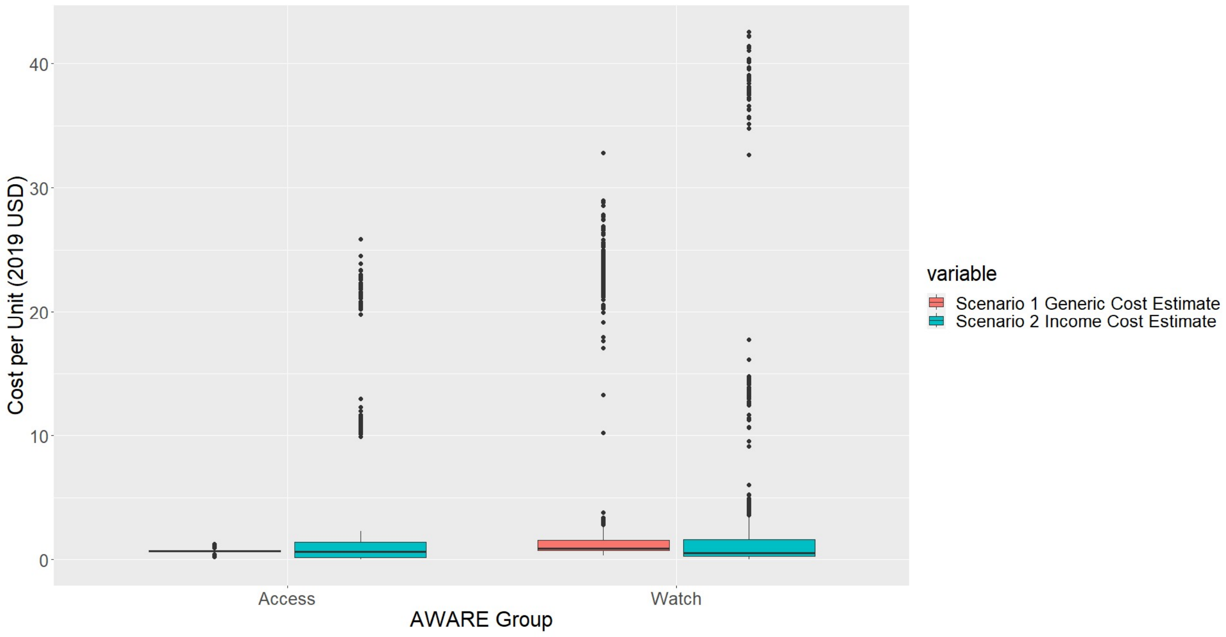

(ii) Zoomed-in with y-axis cut-off specified between 0 and 5, excluding outliers

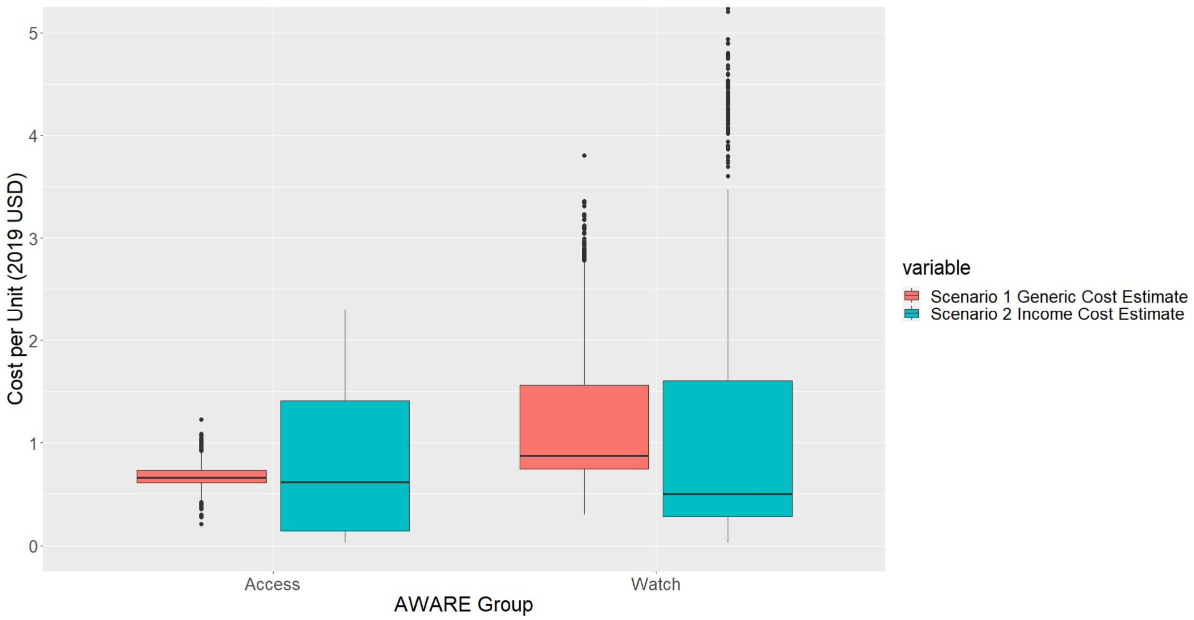

D. Unit productivity costs estimates

Regional average monthly earnings are presented in Table 13. For country level average monthly earnings that have been adjusted by employment rates please refer to the unit cost repository (https://github.com/NikkiR08/AMR-UCR/blob/main/labour_productivity/outputs/labour_wage_2019USD.csv).

Table 13. Average monthly wages (adjusted for employment ratios)

These are the weighted-mean estimates of adjusted nominal, monthly wage in 2019 USD, weighted by country population.

WHO Region	Base Case Scenario where trends are used (USD)	Scenario where latest values are used (USD)
**AFRO**	154	297
**AMRO**	1128	1106
**EMRO**	315	301
**EURO**	1095	1084
**SEARO**	252	197
**WPRO**	709	590

S7. Additional 2019 burden and vaccine impact results

Figure 10. Global costs in 2019 associated with ABR by pathogen and antibiotic class

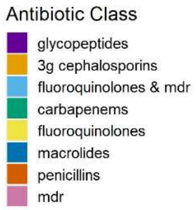

*"fluroquinolones & mdr": These two exposure groups were combined for *Salmonella spp*., due to having only one ABR unit cost available which was a non-specific "ABR in Salmonella" (which was a comparatively very low cost).

A. Median Hospital Costs

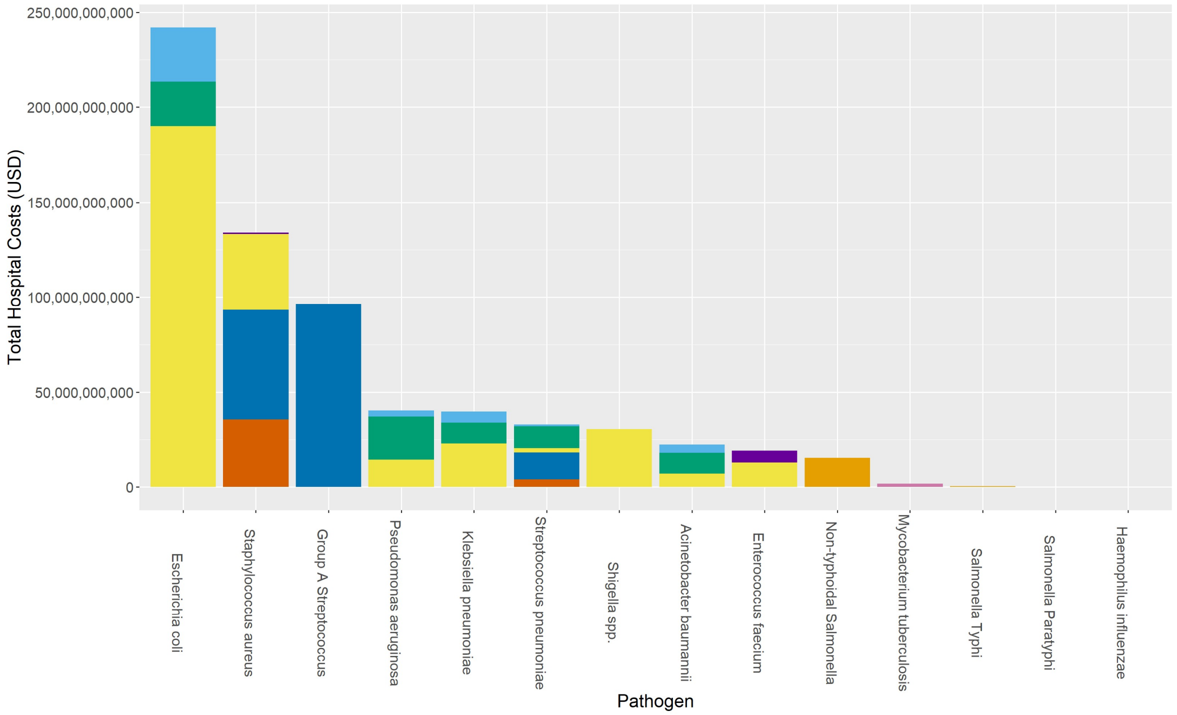

B. Productivity costs due to excess deaths

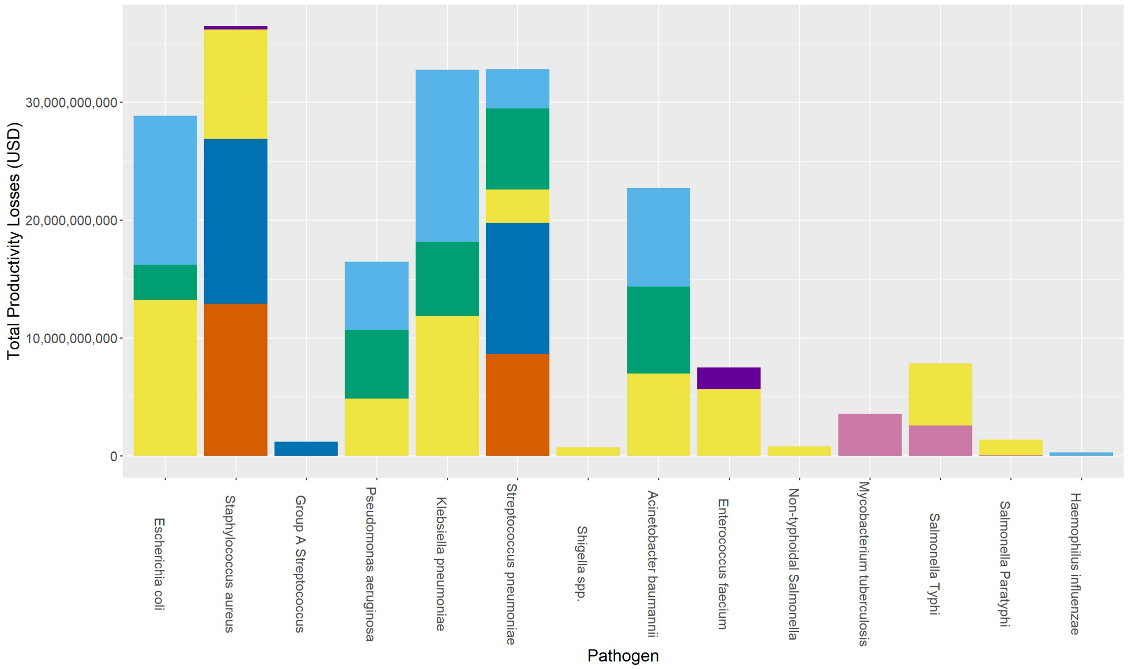

Figure 11. Hospital and productivity costs in 2019 by Pathogen and Syndrome

A. Hospital costs

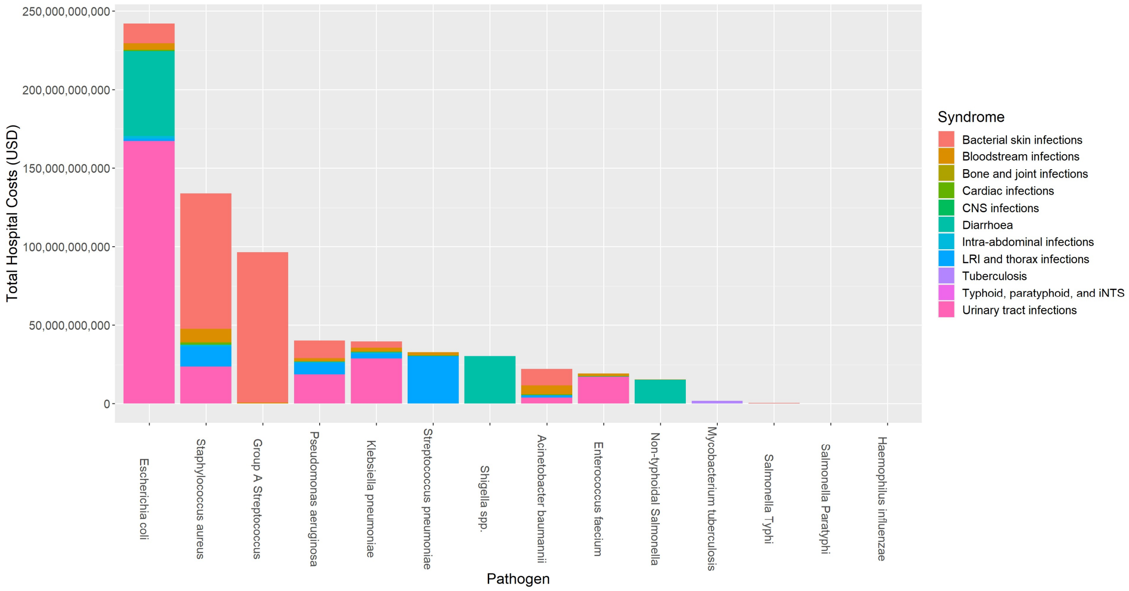

B. Productivity costs

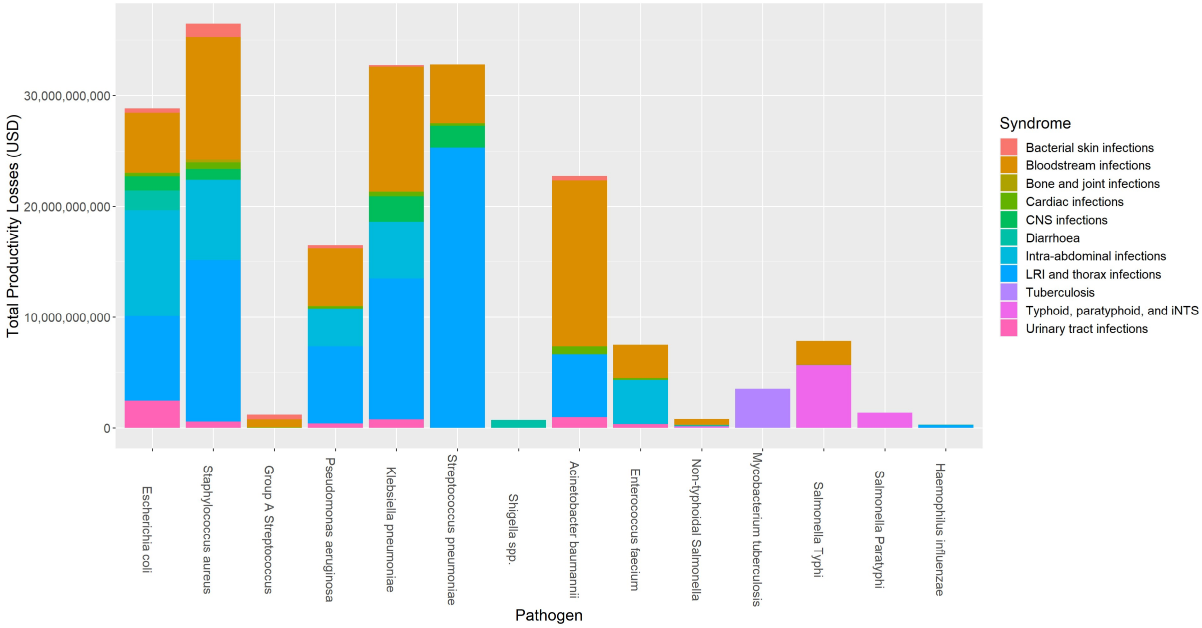

Figure 12, below, compares the different methods for filling in missing data for using earnings and employment rates to estimate labour productivity losses due to excess deaths. Additionally, we explored the use of a friction cost method, whereby only deaths of working age populations were costed. They were costed using 6-month values of WHO-regional averages for employment-adjusted earnings. However, were not included in the main analyses given lack of evidence on appropriate surplus labour thresholds and friction periods, with human capital methods were preferred in the base case as is the case with previous literature[36].

Figure 12 . Productivity Losses by Method of Estimation and WHO Region

Black dots in the centre represent estimates through Friction Cost methods. Human capital scenario 1 refers to trend adjustments being incorporated in the estimation of 2019 employment and earnings. Human capital scenario 2 refers to only earnings being adjusted through GDP deflation mechanisms.

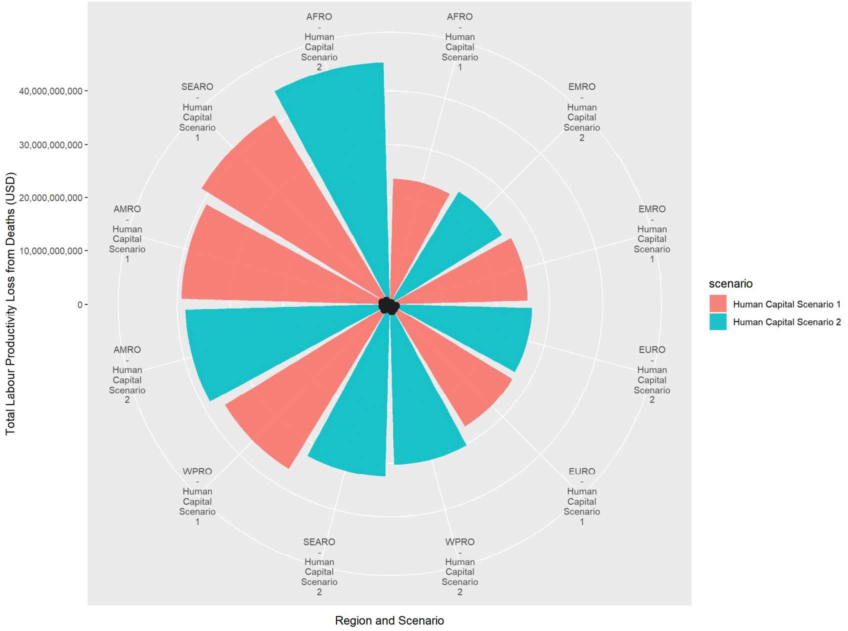

Table 14. Regional values for total hospital cost in 2019

Vaccine scenarios are aligned with those presented in Kim C, Holm M, Frost I, Hasso-Agopsowicz M, Abbas K. Global and regional burden of attributable and associated bacterial antimicrobial resistance avertable by vaccination: modelling study. *BMJ Glob Health* 2023; **8**(7).

Vaccine -target disease	efficacy	coverage	duration	Target population	Infectious syndrome	AFRO	AMRO	EMRO	EURO	SEARO	WPRO
**Acinetobacter baumannii - BSI**	0.7	0.7	5 years	6 weeks & elderly age group	BSI	195,078,429 (162,883,091-237,184,725)	2,132,080,504 (1,786,404,923-2,582,178,488)	800,998,763 (633,925,859-1,022,717,497)	696,419,428 (624,341,014-775,072,302)	373,238,428 (308,288,715-451,795,431)	1,323,329,802 (1,033,210,505-1,687,801,937)
**Acinetobacter baumannii - BSI**	0.7	0.7	5 years	All age groups	BSI	195,078,429 (162,883,091-237,184,725)	2,132,080,504 (1,786,404,923-2,582,178,488)	800,998,763 (633,925,859-1,022,717,497)	696,419,428 (624,341,014-775,072,302)	373,238,428 (308,288,715-451,795,431)	1,323,329,802 (1,033,210,505-1,687,801,937)
**Acinetobacter baumannii - all**	0.7	0.7	5 years	6 weeks & elderly age group	BSI	195,078,429 (162,883,091-237,184,725)	2,132,080,504 (1,786,404,923-2,582,178,488)	800,998,763 (633,925,859-1,022,717,497)	696,419,428 (624,341,014-775,072,302)	373,238,428 (308,288,715-451,795,431)	1,323,329,802 (1,033,210,505-1,687,801,937)
**Acinetobacter baumannii - all**	0.7	0.7	5 years	All age groups	BSI	195,078,429 (162,883,091-237,184,725)	2,132,080,504 (1,786,404,923-2,582,178,488)	800,998,763 (633,925,859-1,022,717,497)	696,419,428 (624,341,014-775,072,302)	373,238,428 (308,288,715-451,795,431)	1,323,329,802 (1,033,210,505-1,687,801,937)
**Acinetobacter baumannii - BSI**	0.7	0.7	5 years	6 weeks & elderly age group	Bacterial skin infections	279,670,215 (239,664,225-332,256,621)	3,031,338,847 (2,494,854,797-3,691,038,830)	776,275,142 (633,205,981-923,061,028)	3,413,852,771 (3,017,323,513-3,904,440,695)	522,124,712 (394,794,521-679,564,523)	2,424,713,857 (2,029,631,923-2,932,185,053)
**Acinetobacter baumannii - BSI**	0.7	0.7	5 years	All age groups	Bacterial skin infections	279,670,215 (239,664,225-332,256,621)	3,031,338,847 (2,494,854,797-3,691,038,830)	776,275,142 (633,205,981-923,061,028)	3,413,852,771 (3,017,323,513-3,904,440,695)	522,124,712 (394,794,521-679,564,523)	2,424,713,857 (2,029,631,923-2,932,185,053)
**Acinetobacter baumannii - all**	0.7	0.7	5 years	6 weeks & elderly age group	Bacterial skin infections	279,670,215 (239,664,225-332,256,621)	3,031,338,847 (2,494,854,797-3,691,038,830)	776,275,142 (633,205,981-923,061,028)	3,413,852,771 (3,017,323,513-3,904,440,695)	522,124,712 (394,794,521-679,564,523)	2,424,713,857 (2,029,631,923-2,932,185,053)
**Acinetobacter baumannii - all**	0.7	0.7	5 years	All age groups	Bacterial skin infections	279,670,215 (239,664,225-332,256,621)	3,031,338,847 (2,494,854,797-3,691,038,830)	776,275,142 (633,205,981-923,061,028)	3,413,852,771 (3,017,323,513-3,904,440,695)	522,124,712 (394,794,521-679,564,523)	2,424,713,857 (2,029,631,923-2,932,185,053)
**Acinetobacter baumannii - BSI**	0.7	0.7	5 years	6 weeks & elderly age group	Cardiac infections	3,657,041 (2,968,438-4,409,939)	262,476,142 (194,003,378-346,811,066)	36,923,789 (28,938,430-48,752,908)	63,602,706 (57,408,139-71,635,847)	15,198,182 (12,655,143-18,570,499)	134,800,038 (105,746,755-173,330,233)
**Acinetobacter baumannii - BSI**	0.7	0.7	5 years	All age groups	Cardiac infections	3,657,041 (2,968,438-4,409,939)	262,476,142 (194,003,378-346,811,066)	36,923,789 (28,938,430-48,752,908)	63,602,706 (57,408,139-71,635,847)	15,198,182 (12,655,143-18,570,499)	134,800,038 (105,746,755-173,330,233)
**Acinetobacter baumannii - all**	0.7	0.7	5 years	6 weeks & elderly age group	Cardiac infections	3,657,041 (2,968,438-4,409,939)	262,476,142 (194,003,378-346,811,066)	36,923,789 (28,938,430-48,752,908)	63,602,706 (57,408,139-71,635,847)	15,198,182 (12,655,143-18,570,499)	134,800,038 (105,746,755-173,330,233)
**Acinetobacter baumannii - all**	0.7	0.7	5 years	All age groups	Cardiac infections	3,657,041 (2,968,438-4,409,939)	262,476,142 (194,003,378-346,811,066)	36,923,789 (28,938,430-48,752,908)	63,602,706 (57,408,139-71,635,847)	15,198,182 (12,655,143-18,570,499)	134,800,038 (105,746,755-173,330,233)
**Acinetobacter baumannii - BSI**	0.7	0.7	5 years	6 weeks & elderly age group	LRI and thorax infections	33,133,110 (27,184,371-39,963,985)	543,822,776 (423,116,734-722,298,932)	130,499,849 (107,074,431-166,892,802)	234,022,794 (205,168,569-267,610,129)	303,079,814 (212,828,474-445,784,159)	192,669,742 (148,878,991-260,978,674)
**Acinetobacter baumannii - BSI**	0.7	0.7	5 years	All age groups	LRI and thorax infections	33,133,110 (27,184,371-39,963,985)	543,822,776 (423,116,734-722,298,932)	130,499,849 (107,074,431-166,892,802)	234,022,794 (205,168,569-267,610,129)	303,079,814 (212,828,474-445,784,159)	192,669,742 (148,878,991-260,978,674)

**Acinetobacter baumannii - all**	0.7	0.7	5 years	6 weeks & elderly age group	LRI and thorax infections	33,133,110 (27,184,371-39,963,985)	543,822,776 (423,116,734-722,298,932)	130,499,849 (107,074,431-166,892,802)	234,022,794 (205,168,569-267,610,129)	303,079,814 (212,828,474-445,784,159)	192,669,742 (148,878,991-260,978,674)
**Acinetobacter baumannii - all**	0.7	0.7	5 years	All age groups	LRI and thorax infections	33,133,110 (27,184,371-39,963,985)	543,822,776 (423,116,734-722,298,932)	130,499,849 (107,074,431-166,892,802)	234,022,794 (205,168,569-267,610,129)	303,079,814 (212,828,474-445,784,159)	192,669,742 (148,878,991-260,978,674)
**Acinetobacter baumannii - BSI**	0.7	0.7	5 years	6 weeks & elderly age group	UTI	48,250,986 (39,579,769-57,127,500)	1,877,043,141 (1,431,948,270-2,470,884,680)	218,625,619 (178,758,358-272,866,078)	601,317,654 (522,450,478-692,052,402)	180,842,281 (134,969,875-240,042,329)	764,415,649 (613,525,923-941,583,292)
**Acinetobacter baumannii - BSI**	0.7	0.7	5 years	All age groups	UTI	48,250,986 (39,579,769-57,127,500)	1,877,043,141 (1,431,948,270-2,470,884,680)	218,625,619 (178,758,358-272,866,078)	601,317,654 (522,450,478-692,052,402)	180,842,281 (134,969,875-240,042,329)	764,415,649 (613,525,923-941,583,292)
**Acinetobacter baumannii - all**	0.7	0.7	5 years	6 weeks & elderly age group	UTI	48,250,986 (39,579,769-57,127,500)	1,877,043,141 (1,431,948,270-2,470,884,680)	218,625,619 (178,758,358-272,866,078)	601,317,654 (522,450,478-692,052,402)	180,842,281 (134,969,875-240,042,329)	764,415,649 (613,525,923-941,583,292)
**Acinetobacter baumannii - all**	0.7	0.7	5 years	All age groups	UTI	48,250,986 (39,579,769-57,127,500)	1,877,043,141 (1,431,948,270-2,470,884,680)	218,625,619 (178,758,358-272,866,078)	601,317,654 (522,450,478-692,052,402)	180,842,281 (134,969,875-240,042,329)	764,415,649 (613,525,923-941,583,292)
**Enterococcus faecium**	0.7	0.7	5 years	6 weeks & elderly age group	BSI	30,725,799 (26,808,459-36,032,591)	699,838,492 (544,242,040-913,862,059)	63,497,858 (56,069,782-73,144,530)	486,105,188 (429,603,489-546,265,314)	23,571,983 (19,852,137-28,267,045)	188,537,250 (160,550,063-223,597,639)
**Enterococcus faecium**	0.7	0.7	5 years	All age groups	BSI	30,725,799 (26,808,459-36,032,591)	699,838,492 (544,242,040-913,862,059)	63,497,858 (56,069,782-73,144,530)	486,105,188 (429,603,489-546,265,314)	23,571,983 (19,852,137-28,267,045)	188,537,250 (160,550,063-223,597,639)
**Enterococcus faecium**	0.7	0.7	5 years	6 weeks & elderly age group	Bone and joint infections	104,175.3 (87,026.79-126,463.2)	9,222,000 (6,197,657-12,739,017)	549,214.2 (404,284.9-735,845.1)	5,163,389 (3,749,367-6,711,872)	267,402.8 (216,547.2-334,829.5)	3,306,868 (2,218,685-4,622,339)
**Enterococcus faecium**	0.7	0.7	5 years	All age groups	Bone and joint infections	104,175.3 (87,026.79-126,463.2)	9,222,000 (6,197,657-12,739,017)	549,214.2 (404,284.9-735,845.1)	5,163,389 (3,749,367-6,711,872)	267,402.8 (216,547.2-334,829.5)	3,306,868 (2,218,685-4,622,339)
**Enterococcus faecium**	0.7	0.7	5 years	6 weeks & elderly age group	Cardiac infections	902,917.9 (727,819.6-1,118,926)	124,823,788 (88,649,123-174,493,460)	5,329,201 (4,181,995-6,843,791)	84,244,991 (74,037,234-95,288,839)	1,017,136 (842,165.6-1,234,908)	22,568,637 (18,992,284-27,522,383)
**Enterococcus faecium**	0.7	0.7	5 years	All age groups	Cardiac infections	902,917.9 (727,819.6-1,118,926)	124,823,788 (88,649,123-174,493,460)	5,329,201 (4,181,995-6,843,791)	84,244,991 (74,037,234-95,288,839)	1,017,136 (842,165.6-1,234,908)	22,568,637 (18,992,284-27,522,383)
**Enterococcus faecium**	0.7	0.7	5 years	6 weeks & elderly age group	Intra-abdominal infections	1,761,932 (1,369,418-2,172,711)	117,257,713 (83,918,890-168,500,535)	4,959,563 (3,823,302-6,309,592)	84,649,778 (62,358,172-108,617,263)	3,910,368 (2,962,414-5,182,096)	37,500,794 (26,939,012-47,650,243)
**Enterococcus faecium**	0.7	0.7	5 years	All age groups	Intra-abdominal infections	1,761,932 (1,369,418-2,172,711)	117,257,713 (83,918,890-168,500,535)	4,959,563 (3,823,302-6,309,592)	84,649,778 (62,358,172-108,617,263)	3,910,368 (2,962,414-5,182,096)	37,500,794 (26,939,012-47,650,243)
**Enterococcus faecium**	0.7	0.7	5 years	6 weeks & elderly age group	UTI	110,407,147 (89,586,652-137,382,315)	9,475,152,533 (6,606,222,726-13,790,132,238)	851,408,196 (670,395,432-1,096,268,963)	3,232,758,410 (2,509,501,824-4,051,453,661)	323,945,845 (230,667,347-442,245,307)	2,879,950,066 (2,163,489,231-3,643,020,846)
**Enterococcus faecium**	0.7	0.7	5 years	All age groups	UTI	110,407,147 (89,586,652-137,382,315)	9,475,152,533 (6,606,222,726-	851,408,196 (670,395,432-1,096,268,963)	3,232,758,410 (2,509,501,824-4,051,453,661)	323,945,845 (230,667,347-442,245,307)	2,879,950,066 (2,163,489,231-3,643,020,846)

							13,790,132,238)				
**E. coli - non-diarrhogenic**	0.7	0.7	5 years	6 weeks & elderly age group	BSI	73,784,644 (65,072,937-85,026,373)	956,450,699 (777,039,317-1,197,058,201)	183,117,669 (158,864,937-213,695,282)	2,300,820,795 (2,049,400,346-2,601,736,441)	95,794,044 (80,803,152-116,188,369)	516,686,561 (428,139,587-613,625,950)
**E. coli - non-diarrhogenic**	0.7	0.7	5 years	All age groups	BSI	73,784,644 (65,072,937-85,026,373)	956,450,699 (777,039,317-1,197,058,201)	183,117,669 (158,864,937-213,695,282)	2,300,820,795 (2,049,400,346-2,601,736,441)	95,794,044 (80,803,152-116,188,369)	516,686,561 (428,139,587-613,625,950)
**ETEC**	0.6	0.7	5 years	6 months	BSI	73,784,644 (65,072,937-85,026,373)	956,450,699 (777,039,317-1,197,058,201)	183,117,669 (158,864,937-213,695,282)	2,300,820,795 (2,049,400,346-2,601,736,441)	95,794,044 (80,803,152-116,188,369)	516,686,561 (428,139,587-613,625,950)
**ExPEC - BSI**	0.7	0.7	5 years	6 weeks & elderly age group	BSI	73,784,644 (65,072,937-85,026,373)	956,450,699 (777,039,317-1,197,058,201)	183,117,669 (158,864,937-213,695,282)	2,300,820,795 (2,049,400,346-2,601,736,441)	95,794,044 (80,803,152-116,188,369)	516,686,561 (428,139,587-613,625,950)
**ExPEC - BSI**	0.7	0.7	5 years	All age groups	BSI	73,784,644 (65,072,937-85,026,373)	956,450,699 (777,039,317-1,197,058,201)	183,117,669 (158,864,937-213,695,282)	2,300,820,795 (2,049,400,346-2,601,736,441)	95,794,044 (80,803,152-116,188,369)	516,686,561 (428,139,587-613,625,950)
**ExPEC - UTI**	0.7	0.7	5 years	6 weeks & elderly age group	BSI	73,784,644 (65,072,937-85,026,373)	956,450,699 (777,039,317-1,197,058,201)	183,117,669 (158,864,937-213,695,282)	2,300,820,795 (2,049,400,346-2,601,736,441)	95,794,044 (80,803,152-116,188,369)	516,686,561 (428,139,587-613,625,950)
**ExPEC - UTI**	0.7	0.7	5 years	All age groups	BSI	73,784,644 (65,072,937-85,026,373)	956,450,699 (777,039,317-1,197,058,201)	183,117,669 (158,864,937-213,695,282)	2,300,820,795 (2,049,400,346-2,601,736,441)	95,794,044 (80,803,152-116,188,369)	516,686,561 (428,139,587-613,625,950)
**E. coli - non-diarrhogenic**	0.7	0.7	5 years	6 weeks & elderly age group	Bacterial skin infections	244,811,733 (209,217,314-285,953,646)	2,026,849,676 (1,649,761,635-2,472,825,576)	979,213,598 (801,048,772-1,185,057,392)	4,749,018,924 (3,943,043,849-5,651,675,537)	825,341,573 (619,034,674-1,092,016,360)	3,322,142,772 (2,695,103,387-3,971,674,630)
**E. coli - non-diarrhogenic**	0.7	0.7	5 years	All age groups	Bacterial skin infections	244,811,733 (209,217,314-285,953,646)	2,026,849,676 (1,649,761,635-2,472,825,576)	979,213,598 (801,048,772-1,185,057,392)	4,749,018,924 (3,943,043,849-5,651,675,537)	825,341,573 (619,034,674-1,092,016,360)	3,322,142,772 (2,695,103,387-3,971,674,630)
**ETEC**	0.6	0.7	5 years	6 months	Bacterial skin infections	244,811,733 (209,217,314-285,953,646)	2,026,849,676 (1,649,761,635-2,472,825,576)	979,213,598 (801,048,772-1,185,057,392)	4,749,018,924 (3,943,043,849-5,651,675,537)	825,341,573 (619,034,674-1,092,016,360)	3,322,142,772 (2,695,103,387-3,971,674,630)
**ExPEC - BSI**	0.7	0.7	5 years	6 weeks & elderly age group	Bacterial skin infections	244,811,733 (209,217,314-285,953,646)	2,026,849,676 (1,649,761,635-2,472,825,576)	979,213,598 (801,048,772-1,185,057,392)	4,749,018,924 (3,943,043,849-5,651,675,537)	825,341,573 (619,034,674-1,092,016,360)	3,322,142,772 (2,695,103,387-3,971,674,630)
**ExPEC - BSI**	0.7	0.7	5 years	All age groups	Bacterial skin infections	244,811,733 (209,217,314-285,953,646)	2,026,849,676 (1,649,761,635-2,472,825,576)	979,213,598 (801,048,772-1,185,057,392)	4,749,018,924 (3,943,043,849-5,651,675,537)	825,341,573 (619,034,674-1,092,016,360)	3,322,142,772 (2,695,103,387-3,971,674,630)
**ExPEC - UTI**	0.7	0.7	5 years	6 weeks & elderly age group	Bacterial skin infections	244,811,733 (209,217,314-285,953,646)	2,026,849,676 (1,649,761,635-2,472,825,576)	979,213,598 (801,048,772-1,185,057,392)	4,749,018,924 (3,943,043,849-5,651,675,537)	825,341,573 (619,034,674-1,092,016,360)	3,322,142,772 (2,695,103,387-3,971,674,630)
**ExPEC - UTI**	0.7	0.7	5 years	All age groups	Bacterial skin infections	244,811,733 (209,217,314-285,953,646)	2,026,849,676 (1,649,761,635-2,472,825,576)	979,213,598 (801,048,772-1,185,057,392)	4,749,018,924 (3,943,043,849-5,651,675,537)	825,341,573 (619,034,674-1,092,016,360)	3,322,142,772 (2,695,103,387-3,971,674,630)
**E. coli - non-diarrhogenic**	0.7	0.7	5 years	6 weeks & elderly age group	Bone and joint infections	227,423.4 (196,345.5-259,657)	7,543,275 (5,340,671-10,580,732)	1,168,048 (919,056.2-1,496,781)	8,936,796 (6,886,017-11,432,614)	1,176,810 (946,554.4-1,475,031)	9,120,933 (6,830,101-12,486,616)

**E. coli - non-diarrhogenic**	0.7	0.7	5 years	All age groups	Bone and joint infections	227,423.4 (196,345.5-259,657)	7,543,275 (5,340,671-10,580,732)	1,168,048 (919,056.2-1,496,781)	8,936,796 (6,886,017-11,432,614)	1,176,810 (946,554.4-1,475,031)	9,120,933 (6,830,101-12,486,616)
**ETEC**	0.6	0.7	5 years	6 months	Bone and joint infections	227,423.4 (196,345.5-259,657)	7,543,275 (5,340,671-10,580,732)	1,168,048 (919,056.2-1,496,781)	8,936,796 (6,886,017-11,432,614)	1,176,810 (946,554.4-1,475,031)	9,120,933 (6,830,101-12,486,616)
**ExPEC - BSI**	0.7	0.7	5 years	6 weeks & elderly age group	Bone and joint infections	227,423.4 (196,345.5-259,657)	7,543,275 (5,340,671-10,580,732)	1,168,048 (919,056.2-1,496,781)	8,936,796 (6,886,017-11,432,614)	1,176,810 (946,554.4-1,475,031)	9,120,933 (6,830,101-12,486,616)
**ExPEC - BSI**	0.7	0.7	5 years	All age groups	Bone and joint infections	227,423.4 (196,345.5-259,657)	7,543,275 (5,340,671-10,580,732)	1,168,048 (919,056.2-1,496,781)	8,936,796 (6,886,017-11,432,614)	1,176,810 (946,554.4-1,475,031)	9,120,933 (6,830,101-12,486,616)
**ExPEC - UTI**	0.7	0.7	5 years	6 weeks & elderly age group	Bone and joint infections	227,423.4 (196,345.5-259,657)	7,543,275 (5,340,671-10,580,732)	1,168,048 (919,056.2-1,496,781)	8,936,796 (6,886,017-11,432,614)	1,176,810 (946,554.4-1,475,031)	9,120,933 (6,830,101-12,486,616)
**ExPEC - UTI**	0.7	0.7	5 years	All age groups	Bone and joint infections	227,423.4 (196,345.5-259,657)	7,543,275 (5,340,671-10,580,732)	1,168,048 (919,056.2-1,496,781)	8,936,796 (6,886,017-11,432,614)	1,176,810 (946,554.4-1,475,031)	9,120,933 (6,830,101-12,486,616)
**E. coli - non-diarrhogenic**	0.7	0.7	5 years	6 weeks & elderly age group	CNS infections	11,646,177 (10,035,264-13,609,738)	14,692,175 (11,510,939-18,773,624)	13,398,308 (11,119,310-16,444,432)	16,711,920 (14,735,362-18,779,669)	13,435,859 (10,425,958-17,957,171)	9,232,600 (7,854,856-10,949,061)
**E. coli - non-diarrhogenic**	0.7	0.7	5 years	All age groups	CNS infections	11,646,177 (10,035,264-13,609,738)	14,692,175 (11,510,939-18,773,624)	13,398,308 (11,119,310-16,444,432)	16,711,920 (14,735,362-18,779,669)	13,435,859 (10,425,958-17,957,171)	9,232,600 (7,854,856-10,949,061)
**ETEC**	0.6	0.7	5 years	6 months	CNS infections	11,646,177 (10,035,264-13,609,738)	14,692,175 (11,510,939-18,773,624)	13,398,308 (11,119,310-16,444,432)	16,711,920 (14,735,362-18,779,669)	13,435,859 (10,425,958-17,957,171)	9,232,600 (7,854,856-10,949,061)
**ExPEC - BSI**	0.7	0.7	5 years	6 weeks & elderly age group	CNS infections	11,646,177 (10,035,264-13,609,738)	14,692,175 (11,510,939-18,773,624)	13,398,308 (11,119,310-16,444,432)	16,711,920 (14,735,362-18,779,669)	13,435,859 (10,425,958-17,957,171)	9,232,600 (7,854,856-10,949,061)
**ExPEC - BSI**	0.7	0.7	5 years	All age groups	CNS infections	11,646,177 (10,035,264-13,609,738)	14,692,175 (11,510,939-18,773,624)	13,398,308 (11,119,310-16,444,432)	16,711,920 (14,735,362-18,779,669)	13,435,859 (10,425,958-17,957,171)	9,232,600 (7,854,856-10,949,061)
**ExPEC - UTI**	0.7	0.7	5 years	6 weeks & elderly age group	CNS infections	11,646,177 (10,035,264-13,609,738)	14,692,175 (11,510,939-18,773,624)	13,398,308 (11,119,310-16,444,432)	16,711,920 (14,735,362-18,779,669)	13,435,859 (10,425,958-17,957,171)	9,232,600 (7,854,856-10,949,061)
**ExPEC - UTI**	0.7	0.7	5 years	All age groups	CNS infections	11,646,177 (10,035,264-13,609,738)	14,692,175 (11,510,939-18,773,624)	13,398,308 (11,119,310-16,444,432)	16,711,920 (14,735,362-18,779,669)	13,435,859 (10,425,958-17,957,171)	9,232,600 (7,854,856-10,949,061)
**E. coli - non-diarrhogenic**	0.7	0.7	5 years	6 weeks & elderly age group	Cardiac infections	2,227,060 (1,841,079-2,710,739)	187,473,554 (137,116,423-252,373,301)	11,964,118 (9,773,474-15,059,727)	395,775,126 (350,578,502-443,812,708)	4,648,757 (3,885,393-5,721,176)	70,509,899 (58,304,701-85,728,855)
**E. coli - non-diarrhogenic**	0.7	0.7	5 years	All age groups	Cardiac infections	2,227,060 (1,841,079-2,710,739)	187,473,554 (137,116,423-252,373,301)	11,964,118 (9,773,474-15,059,727)	395,775,126 (350,578,502-443,812,708)	4,648,757 (3,885,393-5,721,176)	70,509,899 (58,304,701-85,728,855)
**ETEC**	0.6	0.7	5 years	6 months	Cardiac infections	2,227,060 (1,841,079-2,710,739)	187,473,554 (137,116,423-252,373,301)	11,964,118 (9,773,474-15,059,727)	395,775,126 (350,578,502-443,812,708)	4,648,757 (3,885,393-5,721,176)	70,509,899 (58,304,701-85,728,855)

**ExPEC - BSI**	0.7	0.7	5 years	6 weeks & elderly age group	Cardiac infections	2,227,060 (1,841,079-2,710,739)	187,473,554 (137,116,423-252,373,301)	11,964,118 (9,773,474-15,059,727)	395,775,126 (350,578,502-443,812,708)	4,648,757 (3,885,393-5,721,176)	70,509,899 (58,304,701-85,728,855)
**ExPEC - BSI**	0.7	0.7	5 years	All age groups	Cardiac infections	2,227,060 (1,841,079-2,710,739)	187,473,554 (137,116,423-252,373,301)	11,964,118 (9,773,474-15,059,727)	395,775,126 (350,578,502-443,812,708)	4,648,757 (3,885,393-5,721,176)	70,509,899 (58,304,701-85,728,855)
**ExPEC - UTI**	0.7	0.7	5 years	6 weeks & elderly age group	Cardiac infections	2,227,060 (1,841,079-2,710,739)	187,473,554 (137,116,423-252,373,301)	11,964,118 (9,773,474-15,059,727)	395,775,126 (350,578,502-443,812,708)	4,648,757 (3,885,393-5,721,176)	70,509,899 (58,304,701-85,728,855)
**ExPEC - UTI**	0.7	0.7	5 years	All age groups	Cardiac infections	2,227,060 (1,841,079-2,710,739)	187,473,554 (137,116,423-252,373,301)	11,964,118 (9,773,474-15,059,727)	395,775,126 (350,578,502-443,812,708)	4,648,757 (3,885,393-5,721,176)	70,509,899 (58,304,701-85,728,855)
**E. coli - non-diarrhogenic**	0.7	0.7	5 years	6 weeks & elderly age group	Diarrhoea	1,549,173,205 (1,230,690,483-1,962,382,456)	6,807,990,436 (4,804,387,463-9,960,135,455)	13,671,067,305 (10,006,701,554-19,036,817,385)	16,886,814,164 (13,224,692,623-21,795,320,287)	8,221,642,223 (6,113,349,974-10,937,248,420)	2,527,970,843 (2,015,044,771-3,289,110,608)
**E. coli - non-diarrhogenic**	0.7	0.7	5 years	All age groups	Diarrhoea	1,549,173,205 (1,230,690,483-1,962,382,456)	6,807,990,436 (4,804,387,463-9,960,135,455)	13,671,067,305 (10,006,701,554-19,036,817,385)	16,886,814,164 (13,224,692,623-21,795,320,287)	8,221,642,223 (6,113,349,974-10,937,248,420)	2,527,970,843 (2,015,044,771-3,289,110,608)
**ETEC**	0.6	0.7	5 years	6 months	Diarrhoea	1,549,173,205 (1,230,690,483-1,962,382,456)	6,807,990,436 (4,804,387,463-9,960,135,455)	13,671,067,305 (10,006,701,554-19,036,817,385)	16,886,814,164 (13,224,692,623-21,795,320,287)	8,221,642,223 (6,113,349,974-10,937,248,420)	2,527,970,843 (2,015,044,771-3,289,110,608)
**ExPEC - BSI**	0.7	0.7	5 years	6 weeks & elderly age group	Diarrhoea	1,549,173,205 (1,230,690,483-1,962,382,456)	6,807,990,436 (4,804,387,463-9,960,135,455)	13,671,067,305 (10,006,701,554-19,036,817,385)	16,886,814,164 (13,224,692,623-21,795,320,287)	8,221,642,223 (6,113,349,974-10,937,248,420)	2,527,970,843 (2,015,044,771-3,289,110,608)
**ExPEC - BSI**	0.7	0.7	5 years	All age groups	Diarrhoea	1,549,173,205 (1,230,690,483-1,962,382,456)	6,807,990,436 (4,804,387,463-9,960,135,455)	13,671,067,305 (10,006,701,554-19,036,817,385)	16,886,814,164 (13,224,692,623-21,795,320,287)	8,221,642,223 (6,113,349,974-10,937,248,420)	2,527,970,843 (2,015,044,771-3,289,110,608)
**ExPEC - UTI**	0.7	0.7	5 years	6 weeks & elderly age group	Diarrhoea	1,549,173,205 (1,230,690,483-1,962,382,456)	6,807,990,436 (4,804,387,463-9,960,135,455)	13,671,067,305 (10,006,701,554-19,036,817,385)	16,886,814,164 (13,224,692,623-21,795,320,287)	8,221,642,223 (6,113,349,974-10,937,248,420)	2,527,970,843 (2,015,044,771-3,289,110,608)
**ExPEC - UTI**	0.7	0.7	5 years	All age groups	Diarrhoea	1,549,173,205 (1,230,690,483-1,962,382,456)	6,807,990,436 (4,804,387,463-9,960,135,455)	13,671,067,305 (10,006,701,554-19,036,817,385)	16,886,814,164 (13,224,692,623-21,795,320,287)	8,221,642,223 (6,113,349,974-10,937,248,420)	2,527,970,843 (2,015,044,771-3,289,110,608)

**E. coli - non-diarrhogenic**	0.7	0.7	5 years	6 weeks & elderly age group	Intra-abdominal infections	26,455,964 (23,514,454-30,091,262)	464,246,634 (364,809,913-582,011,744)	92,705,485 (77,248,351-111,007,094)	549,287,858 (473,193,558-631,980,647)	152,563,201 (124,010,709-192,067,815)	461,142,274 (386,720,849-562,068,304)
**E. coli - non-diarrhogenic**	0.7	0.7	5 years	All age groups	Intra-abdominal infections	26,455,964 (23,514,454-30,091,262)	464,246,634 (364,809,913-582,011,744)	92,705,485 (77,248,351-111,007,094)	549,287,858 (473,193,558-631,980,647)	152,563,201 (124,010,709-192,067,815)	461,142,274 (386,720,849-562,068,304)
**ETEC**	0.6	0.7	5 years	6 months	Intra-abdominal infections	26,455,964 (23,514,454-30,091,262)	464,246,634 (364,809,913-582,011,744)	92,705,485 (77,248,351-111,007,094)	549,287,858 (473,193,558-631,980,647)	152,563,201 (124,010,709-192,067,815)	461,142,274 (386,720,849-562,068,304)
**ExPEC - BSI**	0.7	0.7	5 years	6 weeks & elderly age group	Intra-abdominal infections	26,455,964 (23,514,454-30,091,262)	464,246,634 (364,809,913-582,011,744)	92,705,485 (77,248,351-111,007,094)	549,287,858 (473,193,558-631,980,647)	152,563,201 (124,010,709-192,067,815)	461,142,274 (386,720,849-562,068,304)
**ExPEC - BSI**	0.7	0.7	5 years	All age groups	Intra-abdominal infections	26,455,964 (23,514,454-30,091,262)	464,246,634 (364,809,913-582,011,744)	92,705,485 (77,248,351-111,007,094)	549,287,858 (473,193,558-631,980,647)	152,563,201 (124,010,709-192,067,815)	461,142,274 (386,720,849-562,068,304)
**ExPEC - UTI**	0.7	0.7	5 years	6 weeks & elderly age group	Intra-abdominal infections	26,455,964 (23,514,454-30,091,262)	464,246,634 (364,809,913-582,011,744)	92,705,485 (77,248,351-111,007,094)	549,287,858 (473,193,558-631,980,647)	152,563,201 (124,010,709-192,067,815)	461,142,274 (386,720,849-562,068,304)
**ExPEC - UTI**	0.7	0.7	5 years	All age groups	Intra-abdominal infections	26,455,964 (23,514,454-30,091,262)	464,246,634 (364,809,913-582,011,744)	92,705,485 (77,248,351-111,007,094)	549,287,858 (473,193,558-631,980,647)	152,563,201 (124,010,709-192,067,815)	461,142,274 (386,720,849-562,068,304)
**E. coli - non-diarrhogenic**	0.7	0.7	5 years	6 weeks & elderly age group	LRI and thorax infections	27,572,563 (21,519,912-34,740,153)	408,497,199 (298,502,611-556,371,366)	104,924,727 (85,148,801-135,601,867)	403,211,341 (326,266,982-484,617,468)	242,021,802 (174,362,180-330,841,084)	290,859,653 (225,436,876-371,319,452)
**E. coli - non-diarrhogenic**	0.7	0.7	5 years	All age groups	LRI and thorax infections	27,572,563 (21,519,912-34,740,153)	408,497,199 (298,502,611-556,371,366)	104,924,727 (85,148,801-135,601,867)	403,211,341 (326,266,982-484,617,468)	242,021,802 (174,362,180-330,841,084)	290,859,653 (225,436,876-371,319,452)
**ETEC**	0.6	0.7	5 years	6 months	LRI and thorax infections	27,572,563 (21,519,912-34,740,153)	408,497,199 (298,502,611-556,371,366)	104,924,727 (85,148,801-135,601,867)	403,211,341 (326,266,982-484,617,468)	242,021,802 (174,362,180-330,841,084)	290,859,653 (225,436,876-371,319,452)
**ExPEC - BSI**	0.7	0.7	5 years	6 weeks & elderly age group	LRI and thorax infections	27,572,563 (21,519,912-34,740,153)	408,497,199 (298,502,611-556,371,366)	104,924,727 (85,148,801-135,601,867)	403,211,341 (326,266,982-484,617,468)	242,021,802 (174,362,180-330,841,084)	290,859,653 (225,436,876-371,319,452)
**ExPEC - BSI**	0.7	0.7	5 years	All age groups	LRI and thorax infections	27,572,563 (21,519,912-34,740,153)	408,497,199 (298,502,611-556,371,366)	104,924,727 (85,148,801-135,601,867)	403,211,341 (326,266,982-484,617,468)	242,021,802 (174,362,180-330,841,084)	290,859,653 (225,436,876-371,319,452)
**ExPEC - UTI**	0.7	0.7	5 years	6 weeks & elderly age group	LRI and thorax infections	27,572,563 (21,519,912-34,740,153)	408,497,199 (298,502,611-556,371,366)	104,924,727 (85,148,801-135,601,867)	403,211,341 (326,266,982-484,617,468)	242,021,802 (174,362,180-330,841,084)	290,859,653 (225,436,876-371,319,452)
**ExPEC - UTI**	0.7	0.7	5 years	All age groups	LRI and thorax infections	27,572,563 (21,519,912-34,740,153)	408,497,199 (298,502,611-556,371,366)	104,924,727 (85,148,801-135,601,867)	403,211,341 (326,266,982-484,617,468)	242,021,802 (174,362,180-330,841,084)	290,859,653 (225,436,876-371,319,452)
**E. coli - non-diarrhogenic**	0.7	0.7	5 years	6 weeks & elderly age group	UTI	1,498,137,944 (1,233,003,714-1,813,548,379)	63,488,776,893 (45,659,330,389-85,700,779,853)	11,666,901,228 (9,293,056,021-15,030,833,995)	32,791,003,816 (26,153,844,513-41,119,881,843)	10,307,253,388 (7,514,088,574-13,703,873,524)	40,649,717,564 (31,449,150,789-49,921,511,235)

**E. coli - non-diarrhogenic**	0.7	0.7	5 years	All age groups	UTI	1,498,137,944 (1,233,003,714-1,813,548,379)	63,488,776,893 (45,659,330,389-85,700,779,853)	11,666,901,228 (9,293,056,021-15,030,833,995)	32,791,003,816 (26,153,844,513-41,119,881,843)	10,307,253,388 (7,514,088,574-13,703,873,524)	40,649,717,564 (31,449,150,789-49,921,511,235)
**ETEC**	0.6	0.7	5 years	6 months	UTI	1,498,137,944 (1,233,003,714-1,813,548,379)	63,488,776,893 (45,659,330,389-85,700,779,853)	11,666,901,228 (9,293,056,021-15,030,833,995)	32,791,003,816 (26,153,844,513-41,119,881,843)	10,307,253,388 (7,514,088,574-13,703,873,524)	40,649,717,564 (31,449,150,789-49,921,511,235)
**ExPEC - BSI**	0.7	0.7	5 years	6 weeks & elderly age group	UTI	1,498,137,944 (1,233,003,714-1,813,548,379)	63,488,776,893 (45,659,330,389-85,700,779,853)	11,666,901,228 (9,293,056,021-15,030,833,995)	32,791,003,816 (26,153,844,513-41,119,881,843)	10,307,253,388 (7,514,088,574-13,703,873,524)	40,649,717,564 (31,449,150,789-49,921,511,235)
**ExPEC - BSI**	0.7	0.7	5 years	All age groups	UTI	1,498,137,944 (1,233,003,714-1,813,548,379)	63,488,776,893 (45,659,330,389-85,700,779,853)	11,666,901,228 (9,293,056,021-15,030,833,995)	32,791,003,816 (26,153,844,513-41,119,881,843)	10,307,253,388 (7,514,088,574-13,703,873,524)	40,649,717,564 (31,449,150,789-49,921,511,235)
**ExPEC - UTI**	0.7	0.7	5 years	6 weeks & elderly age group	UTI	1,498,137,944 (1,233,003,714-1,813,548,379)	63,488,776,893 (45,659,330,389-85,700,779,853)	11,666,901,228 (9,293,056,021-15,030,833,995)	32,791,003,816 (26,153,844,513-41,119,881,843)	10,307,253,388 (7,514,088,574-13,703,873,524)	40,649,717,564 (31,449,150,789-49,921,511,235)
**ExPEC - UTI**	0.7	0.7	5 years	All age groups	UTI	1,498,137,944 (1,233,003,714-1,813,548,379)	63,488,776,893 (45,659,330,389-85,700,779,853)	11,666,901,228 (9,293,056,021-15,030,833,995)	32,791,003,816 (26,153,844,513-41,119,881,843)	10,307,253,388 (7,514,088,574-13,703,873,524)	40,649,717,564 (31,449,150,789-49,921,511,235)
**Group A streptococcus**	0.7	0.7	5 years	6 weeks	BSI	10,641,249 (8,384,221-13,393,369)	207,045,021 (103,491,970-432,659,702)	17,248,488 (13,606,353-22,077,793)	125,327,266 (103,026,278-154,646,627)	10,616,238 (6,795,500-16,048,409)	190,255,302 (100,716,150-327,881,404)
**Group A streptococcus**	0.7	0.7	5 years	6 weeks	Bacterial skin infections	788,712,157 (624,868,187-996,572,251)	16,066,993,609 (8,995,185,049-30,442,956,100)	2,349,661,342 (1,778,012,296-3,028,423,383)	23,832,145,937 (19,069,495,635-29,874,276,152)	1,282,339,486 (767,156,464-2,233,973,868)	41,545,049,686 (24,409,259,774-67,312,837,648)
**Group A streptococcus**	0.7	0.7	5 years	6 weeks	Bone and joint infections	108,401.4 (87,834.17-133,764.5)	12,512,032 (5,752,777-27,699,425)	497,764.5 (374,852.9-660,559)	4,579,945 (3,682,287-5,758,544)	375,547.9 (218,955.2-592,512.6)	12,352,560 (6,984,750-20,396,945)
**Group A streptococcus**	0.7	0.7	5 years	6 weeks	Cardiac infections	121,210.1 (94,006.89-153,376)	7,086,297 (3,380,466-15,377,613)	528,360.4 (397,925.1-698,745)	5,938,864 (4,781,684-7,473,168)	237,778.3 (140,044.7-364,260.8)	7,202,577 (4,039,923-11,616,116)
**Haemophilus influenzae type B**	0.93	0.9	5 years	6, 10, 14 weeks	CNS infections	363,394.3 (281,705-497,240.9)	138,493.4 (89,353.84-220,755.4)	406,494.4 (269,272.5-673,939.5)	258,765.3 (202,655.9-325,005.8)	155,872.9 (88,356.75-267,039.9)	138,010.6 (101,134-181,353.7)

**Haemophilus influenzae type B**	both	0.9	5 years	6, 10, 14 weeks	CNS infections	363,394.3 (281,705-497,240.9)	138,493.4 (89,353.84-220,755.4)	406,494.4 (269,272.5-673,939.5)	258,765.3 (202,655.9-325,005.8)	155,872.9 (88,356.75-267,039.9)	138,010.6 (101,134-181,353.7)
**Haemophilus influenzae type B**	current	current	5 years	6, 10, 14 weeks	CNS infections	363,394.3 (281,705-497,240.9)	138,493.4 (89,353.84-220,755.4)	406,494.4 (269,272.5-673,939.5)	258,765.3 (202,655.9-325,005.8)	155,872.9 (88,356.75-267,039.9)	138,010.6 (101,134-181,353.7)
**Haemophilus influenzae type B**	0.69	0.9	5 years	6, 10, 14 weeks	LRI and thorax infections	2,054,134 (1,607,149-2,639,576)	26,086,004 (14,622,697-48,277,001)	7,973,034 (6,143,146-10,894,871)	23,551,356 (18,734,768-29,729,996)	5,459,968 (3,316,624-9,370,808)	8,089,050 (5,857,720-11,428,245)
**Haemophilus influenzae type B**	both	0.9	5 years	6, 10, 14 weeks	LRI and thorax infections	2,054,134 (1,607,149-2,639,576)	26,086,004 (14,622,697-48,277,001)	7,973,034 (6,143,146-10,894,871)	23,551,356 (18,734,768-29,729,996)	5,459,968 (3,316,624-9,370,808)	8,089,050 (5,857,720-11,428,245)
**Haemophilus influenzae type B**	current	current	5 years	6, 10, 14 weeks	LRI and thorax infections	2,054,134 (1,607,149-2,639,576)	26,086,004 (14,622,697-48,277,001)	7,973,034 (6,143,146-10,894,871)	23,551,356 (18,734,768-29,729,996)	5,459,968 (3,316,624-9,370,808)	8,089,050 (5,857,720-11,428,245)
**Klebsiella pneumoniae - BSI**	0.7	0.7	6 months	0 weeks (maternal)	BSI	167,060,942 (149,266,473-187,732,042)	543,343,367 (473,306,612-647,717,528)	246,089,033 (214,890,676-287,760,902)	1,067,009,312 (928,429,170-1,248,902,902)	165,096,727 (137,614,285-204,545,183)	217,654,935 (175,696,051-269,529,099)
**Klebsiella pneumoniae - all**	0.7	0.7	5 years	6 weeks & elderly age group	BSI	167,060,942 (149,266,473-187,732,042)	543,343,367 (473,306,612-647,717,528)	246,089,033 (214,890,676-287,760,902)	1,067,009,312 (928,429,170-1,248,902,902)	165,096,727 (137,614,285-204,545,183)	217,654,935 (175,696,051-269,529,099)
**Klebsiella pneumoniae - all**	0.7	0.7	5 years	All age groups	BSI	167,060,942 (149,266,473-187,732,042)	543,343,367 (473,306,612-647,717,528)	246,089,033 (214,890,676-287,760,902)	1,067,009,312 (928,429,170-1,248,902,902)	165,096,727 (137,614,285-204,545,183)	217,654,935 (175,696,051-269,529,099)
**Klebsiella pneumoniae - BSI**	0.7	0.7	6 months	0 weeks (maternal)	Bacterial skin infections	134,787,081 (117,626,889-157,940,121)	583,673,538 (498,008,500-684,145,732)	370,775,908 (305,453,251-443,285,485)	1,980,328,210 (1,697,909,080-2,271,517,427)	378,163,078 (278,783,598-502,507,033)	553,544,977 (459,350,560-667,873,282)
**Klebsiella pneumoniae - all**	0.7	0.7	5 years	6 weeks & elderly age group	Bacterial skin infections	134,787,081 (117,626,889-157,940,121)	583,673,538 (498,008,500-684,145,732)	370,775,908 (305,453,251-443,285,485)	1,980,328,210 (1,697,909,080-2,271,517,427)	378,163,078 (278,783,598-502,507,033)	553,544,977 (459,350,560-667,873,282)
**Klebsiella pneumoniae - all**	0.7	0.7	5 years	All age groups	Bacterial skin infections	134,787,081 (117,626,889-157,940,121)	583,673,538 (498,008,500-684,145,732)	370,775,908 (305,453,251-443,285,485)	1,980,328,210 (1,697,909,080-2,271,517,427)	378,163,078 (278,783,598-502,507,033)	553,544,977 (459,350,560-667,873,282)
**Klebsiella pneumoniae - BSI**	0.7	0.7	6 months	0 weeks (maternal)	Bone and joint infections	318,679.4 (278,968.5-363,486.6)	4,024,799 (3,145,664-5,057,662)	1,076,031 (866,896.7-1,346,933)	8,365,948 (6,704,675-10,494,514)	1,037,547 (825,926.8-1,322,711)	3,326,600 (2,542,069-4,548,917)
**Klebsiella pneumoniae - all**	0.7	0.7	5 years	6 weeks & elderly age group	Bone and joint infections	318,679.4 (278,968.5-363,486.6)	4,024,799 (3,145,664-5,057,662)	1,076,031 (866,896.7-1,346,933)	8,365,948 (6,704,675-10,494,514)	1,037,547 (825,926.8-1,322,711)	3,326,600 (2,542,069-4,548,917)
**Klebsiella pneumoniae - all**	0.7	0.7	5 years	All age groups	Bone and joint infections	318,679.4 (278,968.5-363,486.6)	4,024,799 (3,145,664-5,057,662)	1,076,031 (866,896.7-1,346,933)	8,365,948 (6,704,675-10,494,514)	1,037,547 (825,926.8-1,322,711)	3,326,600 (2,542,069-4,548,917)
**Klebsiella pneumoniae - BSI**	0.7	0.7	6 months	0 weeks (maternal)	CNS infections	13,092,604 (11,389,073-15,122,002)	8,085,302 (6,754,075-9,723,571)	12,216,354 (10,090,414-14,991,474)	18,393,247 (16,186,618-21,111,331)	13,753,653 (10,635,201-18,626,083)	4,492,926 (3,820,188-5,411,883)
**Klebsiella pneumoniae - all**	0.7	0.7	5 years	6 weeks & elderly age group	CNS infections	13,092,604 (11,389,073-15,122,002)	8,085,302 (6,754,075-9,723,571)	12,216,354 (10,090,414-14,991,474)	18,393,247 (16,186,618-21,111,331)	13,753,653 (10,635,201-18,626,083)	4,492,926 (3,820,188-5,411,883)

**Klebsiella pneumoniae - all**	0.7	0.7	5 years	All age groups	CNS infections	13,092,604 (11,389,073-15,122,002)	8,085,302 (6,754,075-9,723,571)	12,216,354 (10,090,414-14,991,474)	18,393,247 (16,186,618-21,111,331)	13,753,653 (10,635,201-18,626,083)	4,492,926 (3,820,188-5,411,883)
**Klebsiella pneumoniae - BSI**	0.7	0.7	6 months	0 weeks (maternal)	Cardiac infections	3,811,594 (3,337,082-4,525,003)	67,049,670 (52,673,937-85,276,034)	12,781,314 (10,407,560-15,859,912)	126,036,724 (111,563,969-141,892,744)	6,871,079 (5,619,241-8,705,403)	20,337,084 (16,411,764-25,027,222)
**Klebsiella pneumoniae - all**	0.7	0.7	5 years	6 weeks & elderly age group	Cardiac infections	3,811,594 (3,337,082-4,525,003)	67,049,670 (52,673,937-85,276,034)	12,781,314 (10,407,560-15,859,912)	126,036,724 (111,563,969-141,892,744)	6,871,079 (5,619,241-8,705,403)	20,337,084 (16,411,764-25,027,222)
**Klebsiella pneumoniae - all**	0.7	0.7	5 years	All age groups	Cardiac infections	3,811,594 (3,337,082-4,525,003)	67,049,670 (52,673,937-85,276,034)	12,781,314 (10,407,560-15,859,912)	126,036,724 (111,563,969-141,892,744)	6,871,079 (5,619,241-8,705,403)	20,337,084 (16,411,764-25,027,222)
**Klebsiella pneumoniae - BSI**	0.7	0.7	6 months	0 weeks (maternal)	Intra-abdominal infections	17,291,503 (15,214,167-19,634,751)	194,877,731 (158,967,920-243,067,509)	42,836,426 (35,255,485-50,668,599)	367,089,471 (321,548,785-434,697,651)	78,500,514 (60,559,315-100,888,044)	115,001,552 (92,854,262-142,420,961)
**Klebsiella pneumoniae - all**	0.7	0.7	5 years	6 weeks & elderly age group	Intra-abdominal infections	17,291,503 (15,214,167-19,634,751)	194,877,731 (158,967,920-243,067,509)	42,836,426 (35,255,485-50,668,599)	367,089,471 (321,548,785-434,697,651)	78,500,514 (60,559,315-100,888,044)	115,001,552 (92,854,262-142,420,961)
**Klebsiella pneumoniae - all**	0.7	0.7	5 years	All age groups	Intra-abdominal infections	17,291,503 (15,214,167-19,634,751)	194,877,731 (158,967,920-243,067,509)	42,836,426 (35,255,485-50,668,599)	367,089,471 (321,548,785-434,697,651)	78,500,514 (60,559,315-100,888,044)	115,001,552 (92,854,262-142,420,961)
**Klebsiella pneumoniae - BSI**	0.7	0.7	6 months	0 weeks (maternal)	LRI and thorax infections	66,475,664 (53,308,265-82,246,220)	631,613,186 (503,309,974-796,013,394)	228,795,874 (186,560,930-286,040,127)	1,126,528,631 (933,360,014-1,362,890,540)	526,249,571 (366,680,694-767,737,938)	333,789,257 (258,783,759-437,698,528)
**Klebsiella pneumoniae - all**	0.7	0.7	5 years	6 weeks & elderly age group	LRI and thorax infections	66,475,664 (53,308,265-82,246,220)	631,613,186 (503,309,974-796,013,394)	228,795,874 (186,560,930-286,040,127)	1,126,528,631 (933,360,014-1,362,890,540)	526,249,571 (366,680,694-767,737,938)	333,789,257 (258,783,759-437,698,528)
**Klebsiella pneumoniae - all**	0.7	0.7	5 years	All age groups	LRI and thorax infections	66,475,664 (53,308,265-82,246,220)	631,613,186 (503,309,974-796,013,394)	228,795,874 (186,560,930-286,040,127)	1,126,528,631 (933,360,014-1,362,890,540)	526,249,571 (366,680,694-767,737,938)	333,789,257 (258,783,759-437,698,528)
**Klebsiella pneumoniae - BSI**	0.7	0.7	6 months	0 weeks (maternal)	UTI	350,879,226 (297,974,802-420,246,679)	9,171,548,537 (7,031,895,131-12,159,069,080)	2,217,658,088 (1,782,580,583-2,844,222,457)	9,916,908,036 (8,137,180,098-11,996,884,023)	2,210,441,087 (1,628,332,607-3,005,366,701)	3,867,958,380 (3,132,655,989-4,798,957,430)
**Klebsiella pneumoniae - all**	0.7	0.7	5 years	6 weeks & elderly age group	UTI	350,879,226 (297,974,802-420,246,679)	9,171,548,537 (7,031,895,131-12,159,069,080)	2,217,658,088 (1,782,580,583-2,844,222,457)	9,916,908,036 (8,137,180,098-11,996,884,023)	2,210,441,087 (1,628,332,607-3,005,366,701)	3,867,958,380 (3,132,655,989-4,798,957,430)
**Klebsiella pneumoniae - all**	0.7	0.7	5 years	All age groups	UTI	350,879,226 (297,974,802-420,246,679)	9,171,548,537 (7,031,895,131-12,159,069,080)	2,217,658,088 (1,782,580,583-2,844,222,457)	9,916,908,036 (8,137,180,098-11,996,884,023)	2,210,441,087 (1,628,332,607-3,005,366,701)	3,867,958,380 (3,132,655,989-4,798,957,430)
**Mycobacterium tuberculosis - Improved**	0.8	0.7	10 years	0 weeks + boost every 10 years	TB	29,904,711 (24,502,286-37,378,710)	61,883,067 (49,512,464-77,200,918)	46,599,485 (36,404,427-60,008,486)	1,303,415,255 (1,012,656,050-1,662,966,345)	201,219,000 (118,244,849-320,269,987)	17,660,243 (12,752,558-23,940,216)
**Mycobacterium tuberculosis - M72**	0.5	0.7	10 years	10 years + boost	TB	29,904,711 (24,502,286-37,378,710)	61,883,067 (49,512,464-77,200,918)	46,599,485 (36,404,427-60,008,486)	1,303,415,255 (1,012,656,050-1,662,966,345)	201,219,000 (118,244,849-320,269,987)	17,660,243 (12,752,558-23,940,216)

				every 10 years							
**Non-typhoidal Salmonella**	0.8	0.7	5 years	6 weeks & 9 months	BSI	408,568.2 (343,513.6-491,460.7)	937,752.5 (773,485.1-1,128,843)	1,306,230 (1,002,881-1,709,153)	5,749,140 (4,897,032-6,931,885)	22,846,348 (17,186,105-30,689,440)	18,562,357 (13,262,889-25,234,359)
**Non-typhoidal Salmonella**	0.8	0.7	5 years	6 weeks & 9 months	Cardiac infections	4,303.28 (3,652.363-5,115.258)	27,773.68 (23,735.99-32,717.93)	15,454.27 (13,248.13-18,184.2)	484,716.1 (413,944.1-569,112.5)	464,204.6 (357,342.5-581,746.9)	601,743.2 (428,223.1-816,986.9)
**Non-typhoidal Salmonella**	0.8	0.7	5 years	6 weeks & 9 months	Diarrhoea	70,834,310 (58,410,916-86,968,653)	299,134,989 (254,059,663-361,669,402)	206,403,652 (175,773,571-248,414,585)	4,326,075,753 (3,648,304,034-5,279,755,101)	1,603,999,931 (1,230,368,327-2,061,710,580)	8,186,271,007 (5,723,279,139-11,245,276,611)
**Non-typhoidal Salmonella**	0.8	0.7	5 years	6 weeks & 9 months	Typhoid, paratyphoid, and iNTS	167,272.4 (137,109.2-205,567.1)	90,871.4 (76,092.18-110,729.5)	179,107.3 (149,583-221,293.5)	1,645,136 (1,255,438-2,153,616)	2,112,066 (1,647,801-2,613,038)	1,247,932 (909,995.5-1,652,655)
**Pseudomonas aeruginosa**	0.7	0.7	5 years	6 weeks & elderly age group	BSI	87,743,652 (74,534,395-103,779,156)	817,458,828 (680,654,594-993,702,799)	172,454,741 (148,301,828-204,406,562)	476,576,884 (416,082,309-551,039,064)	92,400,950 (76,790,819-113,878,507)	190,985,556 (154,009,825-230,669,958)
**Pseudomonas aeruginosa**	0.7	0.7	5 years	All age groups	BSI	87,743,652 (74,534,395-103,779,156)	817,458,828 (680,654,594-993,702,799)	172,454,741 (148,301,828-204,406,562)	476,576,884 (416,082,309-551,039,064)	92,400,950 (76,790,819-113,878,507)	190,985,556 (154,009,825-230,669,958)
**Pseudomonas aeruginosa**	0.7	0.7	5 years	6 weeks & elderly age group	Bacterial skin infections	279,642,683 (239,581,068-325,363,033)	3,095,056,676 (2,572,873,515-3,769,371,930)	746,726,688 (616,326,979-872,703,827)	4,409,546,537 (3,782,959,484-5,051,615,545)	781,940,984 (579,699,150-1,014,898,962)	1,625,604,183 (1,354,665,669-1,966,404,892)
**Pseudomonas aeruginosa**	0.7	0.7	5 years	All age groups	Bacterial skin infections	279,642,683 (239,581,068-325,363,033)	3,095,056,676 (2,572,873,515-3,769,371,930)	746,726,688 (616,326,979-872,703,827)	4,409,546,537 (3,782,959,484-5,051,615,545)	781,940,984 (579,699,150-1,014,898,962)	1,625,604,183 (1,354,665,669-1,966,404,892)
**Pseudomonas aeruginosa**	0.7	0.7	5 years	6 weeks & elderly age group	Bone and joint infections	321,022.6 (268,517.7-370,835.6)	17,845,022 (13,469,791-23,276,269)	1,260,961 (1,030,752-1,583,732)	12,346,306 (10,327,052-14,889,054)	827,684.6 (655,971.7-1,046,736)	5,489,438 (4,399,751-6,995,631)
**Pseudomonas aeruginosa**	0.7	0.7	5 years	All age groups	Bone and joint infections	321,022.6 (268,517.7-370,835.6)	17,845,022 (13,469,791-23,276,269)	1,260,961 (1,030,752-1,583,732)	12,346,306 (10,327,052-14,889,054)	827,684.6 (655,971.7-1,046,736)	5,489,438 (4,399,751-6,995,631)
**Pseudomonas aeruginosa**	0.7	0.7	5 years	6 weeks & elderly age group	Cardiac infections	1,535,941 (1,282,932-1,854,051)	120,071,418 (89,376,827-158,571,998)	7,857,888 (6,482,331-9,814,241)	53,517,213 (47,379,714-60,923,250)	3,380,902 (2,759,586-4,200,803)	23,304,497 (19,039,927-28,621,602)
**Pseudomonas aeruginosa**	0.7	0.7	5 years	All age groups	Cardiac infections	1,535,941 (1,282,932-1,854,051)	120,071,418 (89,376,827-158,571,998)	7,857,888 (6,482,331-9,814,241)	53,517,213 (47,379,714-60,923,250)	3,380,902 (2,759,586-4,200,803)	23,304,497 (19,039,927-28,621,602)
**Pseudomonas aeruginosa**	0.7	0.7	5 years	6 weeks & elderly age group	Intra-abdominal infections	6,474,514 (5,436,287-7,530,155)	407,922,383 (310,375,172-534,997,795)	18,039,682 (14,969,343-21,302,399)	268,576,661 (230,446,036-317,924,499)	24,849,103 (19,595,072-31,791,391)	99,118,191 (80,153,666-123,237,074)
**Pseudomonas aeruginosa**	0.7	0.7	5 years	All age groups	Intra-abdominal infections	6,474,514 (5,436,287-7,530,155)	407,922,383 (310,375,172-534,997,795)	18,039,682 (14,969,343-21,302,399)	268,576,661 (230,446,036-317,924,499)	24,849,103 (19,595,072-31,791,391)	99,118,191 (80,153,666-123,237,074)
**Pseudomonas aeruginosa**	0.7	0.7	5 years	6 weeks & elderly age group	LRI and thorax infections	80,167,157 (64,266,289-97,157,566)	3,111,312,414 (2,249,730,631-4,325,526,635)	338,049,740 (282,514,304-421,161,124)	2,440,856,833 (2,101,298,231-2,854,361,000)	456,246,829 (312,422,498-683,624,777)	725,577,456 (559,318,884-950,668,410)

**Pseudomonas aeruginosa**	0.7	0.7	5 years	All age groups	LRI and thorax infections	80,167,157 (64,266,289-97,157,566)	3,111,312,414 (2,249,730,631-4,325,526,635)	338,049,740 (282,514,304-421,161,124)	2,440,856,833 (2,101,298,231-2,854,361,000)	456,246,829 (312,422,498-683,624,777)	725,577,456 (559,318,884-950,668,410)
**Pseudomonas aeruginosa**	0.7	0.7	5 years	6 weeks & elderly age group	UTI	155,560,044 (128,646,178-183,228,150)	10,870,748,374 (8,191,320,182-14,569,094,894)	798,040,277 (664,737,290-989,166,123)	3,354,245,881 (2,831,612,691-3,913,609,281)	781,250,179 (582,267,646-1,061,067,807)	2,347,591,547 (1,957,300,251-2,865,311,435)
**Pseudomonas aeruginosa**	0.7	0.7	5 years	All age groups	UTI	155,560,044 (128,646,178-183,228,150)	10,870,748,374 (8,191,320,182-14,569,094,894)	798,040,277 (664,737,290-989,166,123)	3,354,245,881 (2,831,612,691-3,913,609,281)	781,250,179 (582,267,646-1,061,067,807)	2,347,591,547 (1,957,300,251-2,865,311,435)
**Salmonella paratyphi**	0.7	0.7	5 years	9 months	Typhoid, paratyphoid, and iNTS	753,252 (625,994.9-905,565.7)	4,309,091 (3,381,449-5,544,976)	25,180,718 (19,343,003-33,429,982)	3,423,158 (3,002,087-3,860,410)	130,318,656 (100,416,350-174,670,243)	10,275,545 (8,297,872-12,869,393)
**Salmonella Typhi**	0.85	0.7	15 years	9 months	BSI	34,382,929 (30,980,208-38,253,194)	14,199,030 (12,152,401-16,392,852)	21,754,147 (18,394,609-26,072,892)	12,836,816 (11,499,035-14,356,826)	14,440,093 (11,397,949-18,517,095)	4,205,257 (3,686,622-4,787,787)
**Salmonella Typhi**	0.85	0.7	15 years	9 months	Cardiac infections	303,393.3 (268,818.9-346,199.8)	380,286.5 (312,770.2-459,561.2)	241,478.4 (216,771.4-270,168.1)	507,770.3 (459,833.4-560,488.4)	168,420.5 (133,100-221,322.3)	126,602.5 (109,270.8-148,989)
**Salmonella Typhi**	0.85	0.7	15 years	9 months	Typhoid, paratyphoid, and iNTS	40,342,322 (34,623,566-46,750,479)	7,436,792 (5,707,875-9,722,601)	70,259,203 (59,271,703-84,815,691)	2,630,381 (2,216,735-3,232,857)	207,104,513 (161,245,517-276,380,128)	18,192,687 (15,827,463-20,868,283)
**Shigella**	0.6	0.7	5 years	6 months	Diarrhoea	670,652,723 (456,459,997-1,012,293,659)	6,385,873,978 (4,158,293,242-9,974,188,925)	6,992,517,100 (4,489,218,202-10,960,045,777)	4,434,923,412 (3,260,547,462-6,035,172,304)	3,352,189,830 (2,116,763,751-5,214,575,584)	4,569,305,201 (2,850,014,673-7,122,154,530)
**Staphylococcus aureus**	0.6	0.7	5 years	6 weeks & elderly age group	BSI	165,062,792 (143,542,952-190,341,764)	3,950,118,931 (3,083,889,387-5,059,064,146)	399,326,874 (353,239,494-456,583,716)	1,900,953,387 (1,706,146,323-2,102,215,750)	204,647,523 (172,164,829-253,557,990)	1,612,851,775 (1,310,649,892-1,934,912,778)
**Staphylococcus aureus**	0.6	0.7	5 years	All age groups	BSI	165,062,792 (143,542,952-190,341,764)	3,950,118,931 (3,083,889,387-5,059,064,146)	399,326,874 (353,239,494-456,583,716)	1,900,953,387 (1,706,146,323-2,102,215,750)	204,647,523 (172,164,829-253,557,990)	1,612,851,775 (1,310,649,892-1,934,912,778)
**Staphylococcus aureus**	0.6	0.7	5 years	6 weeks & elderly age group	Bacterial skin infections	3,309,671,376 (2,804,262,338-3,963,359,679)	18,692,878,790 (15,421,260,999-23,358,854,285)	8,010,773,713 (6,784,721,668-9,513,259,454)	25,378,393,254 (21,316,926,190-30,249,286,268)	6,772,314,255 (4,873,634,447-9,286,013,302)	21,770,481,610 (17,597,062,765-26,375,190,984)
**Staphylococcus aureus**	0.6	0.7	5 years	All age groups	Bacterial skin infections	3,309,671,376 (2,804,262,338-3,963,359,679)	18,692,878,790 (15,421,260,999-23,358,854,285)	8,010,773,713 (6,784,721,668-9,513,259,454)	25,378,393,254 (21,316,926,190-30,249,286,268)	6,772,314,255 (4,873,634,447-9,286,013,302)	21,770,481,610 (17,597,062,765-26,375,190,984)
**Staphylococcus aureus**	0.6	0.7	5 years	6 weeks & elderly age group	Bone and joint infections	2,065,774 (1,667,635-2,596,569)	111,534,104 (77,665,066-157,394,988)	13,090,921 (9,823,239-17,780,292)	71,025,679 (54,937,642-91,823,923)	5,409,624 (4,383,193-6,787,247)	85,856,656 (56,166,267-121,048,851)

**Staphylococcus aureus**	0.6	0.7	5 years	All age groups	Bone and joint infections	2,065,774 (1,667,635-2,596,569)	111,534,104 (77,665,066-157,394,988)	13,090,921 (9,823,239-17,780,292)	71,025,679 (54,937,642-91,823,923)	5,409,624 (4,383,193-6,787,247)	85,856,656 (56,166,267-121,048,851)
**Staphylococcus aureus**	0.6	0.7	5 years	6 weeks & elderly age group	CNS infections	15,265,398 (13,238,697-17,710,112)	46,288,896 (34,970,344-60,615,480)	20,957,377 (17,616,829-25,651,646)	27,897,722 (24,830,495-31,036,068)	17,112,708 (12,914,733-23,021,750)	18,728,105 (16,083,922-22,429,495)
**Staphylococcus aureus**	0.6	0.7	5 years	All age groups	CNS infections	15,265,398 (13,238,697-17,710,112)	46,288,896 (34,970,344-60,615,480)	20,957,377 (17,616,829-25,651,646)	27,897,722 (24,830,495-31,036,068)	17,112,708 (12,914,733-23,021,750)	18,728,105 (16,083,922-22,429,495)
**Staphylococcus aureus**	0.6	0.7	5 years	6 weeks & elderly age group	Cardiac infections	5,476,983 (4,468,472-6,681,791)	717,467,494 (510,802,818-981,163,096)	33,022,301 (26,922,407-41,517,409)	282,703,886 (250,557,183-317,235,617)	9,816,013 (8,077,745-12,165,800)	193,554,903 (160,221,123-236,195,940)
**Staphylococcus aureus**	0.6	0.7	5 years	All age groups	Cardiac infections	5,476,983 (4,468,472-6,681,791)	717,467,494 (510,802,818-981,163,096)	33,022,301 (26,922,407-41,517,409)	282,703,886 (250,557,183-317,235,617)	9,816,013 (8,077,745-12,165,800)	193,554,903 (160,221,123-236,195,940)
**Staphylococcus aureus**	0.6	0.7	5 years	6 weeks & elderly age group	Intra-abdominal infections	8,829,904 (7,692,022-10,112,522)	453,382,740 (357,393,661-590,180,172)	27,735,101 (23,920,409-32,460,037)	245,511,538 (213,345,593-281,281,833)	26,082,604 (21,658,132-32,765,902)	207,638,986 (172,083,222-250,980,569)
**Staphylococcus aureus**	0.6	0.7	5 years	All age groups	Intra-abdominal infections	8,829,904 (7,692,022-10,112,522)	453,382,740 (357,393,661-590,180,172)	27,735,101 (23,920,409-32,460,037)	245,511,538 (213,345,593-281,281,833)	26,082,604 (21,658,132-32,765,902)	207,638,986 (172,083,222-250,980,569)
**Staphylococcus aureus**	0.6	0.7	5 years	6 weeks & elderly age group	LRI and thorax infections	85,290,795 (62,800,726-112,909,959)	5,144,376,371 (3,399,703,690-7,433,283,097)	353,862,930 (264,661,903-447,193,982)	4,473,031,721 (3,681,731,684-5,381,611,399)	393,731,055 (258,135,219-574,426,351)	1,833,887,731 (1,373,071,251-2,527,801,462)
**Staphylococcus aureus**	0.6	0.7	5 years	All age groups	LRI and thorax infections	85,290,795 (62,800,726-112,909,959)	5,144,376,371 (3,399,703,690-7,433,283,097)	353,862,930 (264,661,903-447,193,982)	4,473,031,721 (3,681,731,684-5,381,611,399)	393,731,055 (258,135,219-574,426,351)	1,833,887,731 (1,373,071,251-2,527,801,462)
**Staphylococcus aureus**	0.6	0.7	5 years	6 weeks & elderly age group	UTI	218,912,705 (179,482,381-272,664,794)	11,778,853,934 (8,229,482,923-16,194,707,634)	1,325,347,531 (1,050,954,040-1,663,108,231)	3,492,898,297 (2,750,647,305-4,490,006,536)	997,971,941 (707,417,797-1,423,212,502)	5,174,908,728 (4,002,468,695-6,359,996,511)
**Staphylococcus aureus**	0.6	0.7	5 years	All age groups	UTI	218,912,705 (179,482,381-272,664,794)	11,778,853,934 (8,229,482,923-16,194,707,634)	1,325,347,531 (1,050,954,040-1,663,108,231)	3,492,898,297 (2,750,647,305-4,490,006,536)	997,971,941 (707,417,797-1,423,212,502)	5,174,908,728 (4,002,468,695-6,359,996,511)
**Streptococcus pneumoniae - Improved**	both	0.9	5 years	6 weeks	BSI	80,534,439 (69,040,315-92,613,534)	816,973,532 (665,958,197-1,036,770,159)	155,111,564 (133,125,796-180,312,790)	457,014,668 (406,262,040-507,324,624)	60,198,263 (49,578,894-73,807,660)	421,817,222 (341,476,529-526,547,243)
**Streptococcus pneumoniae - Improved**	both	0.9	5 years	6 weeks & elderly age group	BSI	80,534,439 (69,040,315-92,613,534)	816,973,532 (665,958,197-1,036,770,159)	155,111,564 (133,125,796-180,312,790)	457,014,668 (406,262,040-507,324,624)	60,198,263 (49,578,894-73,807,660)	421,817,222 (341,476,529-526,547,243)
**Streptococcus pneumoniae**	both	0.9	5 years	6, 10, 14 weeks	BSI	80,534,439 (69,040,315-92,613,534)	816,973,532 (665,958,197-1,036,770,159)	155,111,564 (133,125,796-180,312,790)	457,014,668 (406,262,040-507,324,624)	60,198,263 (49,578,894-73,807,660)	421,817,222 (341,476,529-526,547,243)
**Streptococcus pneumoniae**	both	0.9	5 years	6, 10, 14 weeks &	BSI	80,534,439 (69,040,315-92,613,534)	816,973,532 (665,958,197-1,036,770,159)	155,111,564 (133,125,796-180,312,790)	457,014,668 (406,262,040-507,324,624)	60,198,263 (49,578,894-73,807,660)	421,817,222 (341,476,529-526,547,243)

				elderly age group							
**Streptococcus pneumoniae**	current	current	5 years	6, 10, 14 weeks	BSI	80,534,439 (69,040,315-92,613,534)	816,973,532 (665,958,197-1,036,770,159)	155,111,564 (133,125,796-180,312,790)	457,014,668 (406,262,040-507,324,624)	60,198,263 (49,578,894-73,807,660)	421,817,222 (341,476,529-526,547,243)
**Streptococcus pneumoniae - Improved**	both	0.9	5 years	6 weeks	CNS infections	22,285,827 (19,671,881-25,421,820)	55,519,260 (44,297,694-70,200,969)	43,793,029 (35,746,415-55,195,240)	31,060,064 (27,753,514-35,051,824)	19,307,119 (14,998,725-25,514,702)	43,565,903 (36,105,550-52,591,980)
**Streptococcus pneumoniae - Improved**	both	0.9	5 years	6 weeks & elderly age group	CNS infections	22,285,827 (19,671,881-25,421,820)	55,519,260 (44,297,694-70,200,969)	43,793,029 (35,746,415-55,195,240)	31,060,064 (27,753,514-35,051,824)	19,307,119 (14,998,725-25,514,702)	43,565,903 (36,105,550-52,591,980)
**Streptococcus pneumoniae**	both	0.9	5 years	6, 10, 14 weeks	CNS infections	22,285,827 (19,671,881-25,421,820)	55,519,260 (44,297,694-70,200,969)	43,793,029 (35,746,415-55,195,240)	31,060,064 (27,753,514-35,051,824)	19,307,119 (14,998,725-25,514,702)	43,565,903 (36,105,550-52,591,980)
**Streptococcus pneumoniae**	both	0.9	5 years	6, 10, 14 weeks & elderly age group	CNS infections	22,285,827 (19,671,881-25,421,820)	55,519,260 (44,297,694-70,200,969)	43,793,029 (35,746,415-55,195,240)	31,060,064 (27,753,514-35,051,824)	19,307,119 (14,998,725-25,514,702)	43,565,903 (36,105,550-52,591,980)
**Streptococcus pneumoniae**	current	current	5 years	6, 10, 14 weeks	CNS infections	22,285,827 (19,671,881-25,421,820)	55,519,260 (44,297,694-70,200,969)	43,793,029 (35,746,415-55,195,240)	31,060,064 (27,753,514-35,051,824)	19,307,119 (14,998,725-25,514,702)	43,565,903 (36,105,550-52,591,980)
**Streptococcus pneumoniae - Improved**	both	0.9	5 years	6 weeks	Cardiac infections	2,157,852 (1,767,752-2,599,039)	81,819,065 (62,154,015-107,132,686)	10,981,513 (8,920,794-13,895,976)	52,127,577 (46,373,493-59,715,671)	2,215,714 (1,838,297-2,716,331)	36,042,268 (29,589,027-44,464,998)
**Streptococcus pneumoniae - Improved**	both	0.9	5 years	6 weeks & elderly age group	Cardiac infections	2,157,852 (1,767,752-2,599,039)	81,819,065 (62,154,015-107,132,686)	10,981,513 (8,920,794-13,895,976)	52,127,577 (46,373,493-59,715,671)	2,215,714 (1,838,297-2,716,331)	36,042,268 (29,589,027-44,464,998)
**Streptococcus pneumoniae**	both	0.9	5 years	6, 10, 14 weeks	Cardiac infections	2,157,852 (1,767,752-2,599,039)	81,819,065 (62,154,015-107,132,686)	10,981,513 (8,920,794-13,895,976)	52,127,577 (46,373,493-59,715,671)	2,215,714 (1,838,297-2,716,331)	36,042,268 (29,589,027-44,464,998)
**Streptococcus pneumoniae**	both	0.9	5 years	6, 10, 14 weeks & elderly age group	Cardiac infections	2,157,852 (1,767,752-2,599,039)	81,819,065 (62,154,015-107,132,686)	10,981,513 (8,920,794-13,895,976)	52,127,577 (46,373,493-59,715,671)	2,215,714 (1,838,297-2,716,331)	36,042,268 (29,589,027-44,464,998)
**Streptococcus pneumoniae**	current	current	5 years	6, 10, 14 weeks	Cardiac infections	2,157,852 (1,767,752-2,599,039)	81,819,065 (62,154,015-107,132,686)	10,981,513 (8,920,794-13,895,976)	52,127,577 (46,373,493-59,715,671)	2,215,714 (1,838,297-2,716,331)	36,042,268 (29,589,027-44,464,998)
**Streptococcus pneumoniae - Improved**	both	0.9	5 years	6 weeks	LRI and thorax infections	422,038,155 (340,817,089-521,588,316)	11,825,598,870 (9,032,368,811-15,727,123,531)	2,293,381,842 (1,702,899,374-2,994,012,210)	5,454,475,728 (4,649,777,126-6,357,896,941)	1,876,198,866 (1,366,869,481-2,547,059,655)	7,303,563,481 (5,525,253,733-9,856,586,251)
**Streptococcus pneumoniae - Improved**	both	0.9	5 years	6 weeks & elderly age group	LRI and thorax infections	422,038,155 (340,817,089-521,588,316)	11,825,598,870 (9,032,368,811-15,727,123,531)	2,293,381,842 (1,702,899,374-2,994,012,210)	5,454,475,728 (4,649,777,126-6,357,896,941)	1,876,198,866 (1,366,869,481-2,547,059,655)	7,303,563,481 (5,525,253,733-9,856,586,251)

**Streptococcus pneumoniae**	both	0.9	5 years	6, 10, 14 weeks	LRI and thorax infections	422,038,155 (340,817,089-521,588,316)	11,825,598,870 (9,032,368,811-15,727,123,531)	2,293,381,842 (1,702,899,374-2,994,012,210)	5,454,475,728 (4,649,777,126-6,357,896,941)	1,876,198,866 (1,366,869,481-2,547,059,655)	7,303,563,481 (5,525,253,733-9,856,586,251)
**Streptococcus pneumoniae**	both	0.9	5 years	6, 10, 14 weeks & elderly age group	LRI and thorax infections	422,038,155 (340,817,089-521,588,316)	11,825,598,870 (9,032,368,811-15,727,123,531)	2,293,381,842 (1,702,899,374-2,994,012,210)	5,454,475,728 (4,649,777,126-6,357,896,941)	1,876,198,866 (1,366,869,481-2,547,059,655)	7,303,563,481 (5,525,253,733-9,856,586,251)
**Streptococcus pneumoniae**	current	current	5 years	6, 10, 14 weeks	LRI and thorax infections	422,038,155 (340,817,089-521,588,316)	11,825,598,870 (9,032,368,811-15,727,123,531)	2,293,381,842 (1,702,899,374-2,994,012,210)	5,454,475,728 (4,649,777,126-6,357,896,941)	1,876,198,866 (1,366,869,481-2,547,059,655)	7,303,563,481 (5,525,253,733-9,856,586,251)

Table 15. Regional values for averted hospital cost in 2019

Vaccine scenarios are aligned with those presented in Kim C, Holm M, Frost I, Hasso-Agopsowicz M, Abbas K. Global and regional burden of attributable and associated bacterial antimicrobial resistance avertable by vaccination: modelling study. *BMJ Glob Health* 2023; **8**(7).

**Vaccine - Target disease**	**efficacy**	**coverage**	**duration**	**Target population**	**Infectious syndrome**	**AFRO**	**AMRO**	**EMRO**	**EURO**	**SEARO**	**WPRO**
**Acinetobacter baumannii - BSI**	0.7	0.7	5 years	6 weeks & elderly age group	BSI	2,271,900 (1,903,706-2,766,804)	43,498,687 (35,219,611-53,612,042)	9,690,629 (7,605,391-12,458,921)	14,072,238 (12,811,433-15,745,068)	5,582,793 (4,616,012-6,653,869)	28,675,276 (23,025,069-35,340,993)
**Acinetobacter baumannii - BSI**	0.7	0.7	5 years	All age groups	BSI	95,588,430 (79,812,714-116,220,515)	1,044,719,447 (875,338,412-1,265,267,459)	392,489,394 (310,623,671-501,131,574)	341,245,520 (305,927,097-379,785,428)	182,886,830 (151,061,470-221,379,761)	648,431,603 (506,273,147-827,022,949)
**Acinetobacter baumannii - all**	0.7	0.7	5 years	6 weeks & elderly age group	BSI	2,271,900 (1,903,706-2,766,804)	43,498,687 (35,219,611-53,612,042)	9,690,629 (7,605,391-12,458,921)	14,072,238 (12,811,433-15,745,068)	5,582,793 (4,616,012-6,653,869)	28,675,276 (23,025,069-35,340,993)
**Acinetobacter baumannii - all**	0.7	0.7	5 years	All age groups	BSI	95,588,430 (79,812,714-116,220,515)	1,044,719,447 (875,338,412-1,265,267,459)	392,489,394 (310,623,671-501,131,574)	341,245,520 (305,927,097-379,785,428)	182,886,830 (151,061,470-221,379,761)	648,431,603 (506,273,147-827,022,949)
**Acinetobacter baumannii - BSI**	0.7	0.7	5 years	6 weeks & elderly age group	Bacterial skin infections	0 (0-0)	0 (0-0)	0 (0-0)	0 (0-0)	0 (0-0)	0 (0-0)
**Acinetobacter baumannii - BSI**	0.7	0.7	5 years	All age groups	Bacterial skin infections	0 (0-0)	0 (0-0)	0 (0-0)	0 (0-0)	0 (0-0)	0 (0-0)
**Acinetobacter baumannii - all**	0.7	0.7	5 years	6 weeks & elderly age group	Bacterial skin infections	19,986,881 (17,327,052-23,377,418)	170,712,256 (140,004,230-208,610,777)	39,600,945 (32,781,320-47,382,942)	149,502,000 (132,176,837-169,242,637)	27,840,678 (21,211,871-36,014,934)	149,597,159 (123,986,116-180,091,899)
**Acinetobacter baumannii - all**	0.7	0.7	5 years	All age groups	Bacterial skin infections	137,038,405 (117,435,470-162,805,744)	1,485,356,035 (1,222,478,850-1,808,609,027)	380,374,820 (310,270,931-452,299,904)	1,672,787,858 (1,478,488,521-1,913,175,941)	255,841,109 (193,449,315-332,986,616)	1,188,109,790 (994,519,642-1,436,770,676)

**Acinetobacter baumannii - BSI**	0.7	0.7	5 years	6 weeks & elderly age group	Cardiac infections	0 (0-0)	0 (0-0)	0 (0-0)	0 (0-0)	0 (0-0)	0 (0-0)
**Acinetobacter baumannii - BSI**	0.7	0.7	5 years	All age groups	Cardiac infections	0 (0-0)	0 (0-0)	0 (0-0)	0 (0-0)	0 (0-0)	0 (0-0)
**Acinetobacter baumannii - all**	0.7	0.7	5 years	6 weeks & elderly age group	Cardiac infections	257,667.9 (210,576.8-313,574.9)	16,688,887 (12,310,274-22,037,301)	2,407,361 (1,911,597-3,229,664)	3,507,499 (3,143,084-3,969,953)	855,177.8 (713,129-1,033,815)	9,439,068 (7,375,963-12,342,295)
**Acinetobacter baumannii - all**	0.7	0.7	5 years	All age groups	Cardiac infections	1,791,950 (1,454,535-2,160,870)	128,613,310 (95,061,655-169,937,422)	18,092,657 (14,179,831-23,888,925)	31,165,326 (28,129,988-35,101,565)	7,447,109 (6,201,020-9,099,544)	66,052,018 (51,815,910-84,931,814)
**Acinetobacter baumannii - BSI**	0.7	0.7	5 years	6 weeks & elderly age group	LRI and thorax infections	0 (0-0)	0 (0-0)	0 (0-0)	0 (0-0)	0 (0-0)	0 (0-0)
**Acinetobacter baumannii - BSI**	0.7	0.7	5 years	All age groups	LRI and thorax infections	0 (0-0)	0 (0-0)	0 (0-0)	0 (0-0)	0 (0-0)	0 (0-0)
**Acinetobacter baumannii - all**	0.7	0.7	5 years	6 weeks & elderly age group	LRI and thorax infections	1,816,912 (1,516,655-2,191,280)	27,535,054 (21,609,137-35,994,255)	5,927,380 (4,918,870-7,513,698)	12,449,955 (10,939,467-14,237,851)	12,804,568 (9,093,666-18,683,643)	9,885,433 (7,628,612-13,499,332)
**Acinetobacter baumannii - all**	0.7	0.7	5 years	All age groups	LRI and thorax infections	16,235,224 (13,320,342-19,582,353)	266,473,160 (207,327,199-353,926,477)	63,944,926 (52,466,471-81,777,473)	114,671,169 (100,532,599-131,128,963)	148,509,109 (104,285,952-218,434,238)	94,408,174 (72,950,706-127,879,550)
**Acinetobacter baumannii - BSI**	0.7	0.7	5 years	6 weeks & elderly age group	UTI	0 (0-0)	0 (0-0)	0 (0-0)	0 (0-0)	0 (0-0)	0 (0-0)
**Acinetobacter baumannii - BSI**	0.7	0.7	5 years	All age groups	UTI	0 (0-0)	0 (0-0)	0 (0-0)	0 (0-0)	0 (0-0)	0 (0-0)
**Acinetobacter baumannii - all**	0.7	0.7	5 years	6 weeks & elderly age group	UTI	1,040,773 (862,427.9-1,265,620)	51,594,186 (39,370,516-67,377,798)	4,811,028 (3,950,146-6,017,252)	14,357,183 (12,289,246-16,849,119)	2,275,617 (1,699,619-3,027,773)	17,215,119 (13,972,903-20,998,069)
**Acinetobacter baumannii - all**	0.7	0.7	5 years	All age groups	UTI	23,642,983 (19,394,087-27,992,475)	919,751,139 (701,654,652-1,210,733,493)	107,126,553 (87,591,595-133,704,378)	294,645,651 (256,000,734-339,105,677)	88,612,718 (66,135,239-117,620,741)	374,563,668 (300,627,702-461,375,813)
**Enterococcus faecium**	0.7	0.7	5 years	6 weeks & elderly age group	BSI	455,877.9 (396,132.5-532,138.4)	26,343,342 (20,312,993-34,981,343)	1,007,806 (879,850.1-1,161,484)	18,332,350 (16,224,825-20,617,265)	485,301.1 (408,446-581,201)	7,512,761 (6,370,534-8,796,949)
**Enterococcus faecium**	0.7	0.7	5 years	All age groups	BSI	15,055,642 (13,136,145-17,655,970)	342,920,861 (266,678,600-447,792,409)	31,113,950 (27,474,193-35,840,819)	238,191,542 (210,505,710-267,670,004)	11,550,272 (9,727,547-13,850,852)	92,383,253 (78,669,531-109,562,843)
**Enterococcus faecium**	0.7	0.7	5 years	6 weeks & elderly age group	Bone and joint infections	5,080.81 (4,282.315-6,086.569)	508,805.3 (337,515.3-706,124.2)	21,429.13 (16,078.81-28,893.38)	256,631.6 (190,201.2-333,445.7)	15,191.73 (12,181.35-19,227.11)	168,605.6 (113,029.2-238,004.8)
**Enterococcus faecium**	0.7	0.7	5 years	All age groups	Bone and joint infections	51,045.9 (42,643.13-61,966.99)	4,518,780 (3,036,852-6,242,118)	269,115 (198,099.6-360,564.1)	2,530,061 (1,837,190-3,288,817)	131,027.3 (106,108.1-164,066.4)	1,620,365 (1,087,156-2,264,946)

**Enterococcus faecium**	0.7	0.7	5 years	6 weeks & elderly age group	Cardiac infections	73,930.46 (60,467.47-92,191.68)	8,074,670 (5,756,559-11,314,254)	322,695.5 (255,521.7-417,695.9)	5,585,237 (4,939,307-6,310,615)	61,666.79 (50,990.3-74,811.09)	1,404,587 (1,187,829-1,687,923)
**Enterococcus faecium**	0.7	0.7	5 years	All age groups	Cardiac infections	442,429.8 (356,631.6-548,273.6)	61,163,656 (43,438,070-85,501,795)	2,611,308 (2,049,178-3,353,457)	41,280,046 (36,278,245-46,691,531)	498,396.5 (412,661.1-605,104.7)	11,058,632 (9,306,219-13,485,968)
**Enterococcus faecium**	0.7	0.7	5 years	6 weeks & elderly age group	Intra-abdominal infections	113,701 (88,642.14-140,167.3)	6,959,776 (4,977,813-10,002,699)	288,125.5 (221,701.8-362,024.1)	4,104,916 (3,103,363-5,168,836)	232,998.2 (174,339.3-311,109.1)	1,769,382 (1,308,877-2,287,940)
**Enterococcus faecium**	0.7	0.7	5 years	All age groups	Intra-abdominal infections	863,346.6 (671,014.7-1,064,629)	57,456,279 (41,120,256-82,565,262)	2,430,186 (1,873,418-3,091,700)	41,478,391 (30,555,504-53,222,459)	1,916,080 (1,451,583-2,539,227)	18,375,389 (13,200,116-23,348,619)
**Enterococcus faecium**	0.7	0.7	5 years	6 weeks & elderly age group	UTI	3,244,585 (2,612,241-4,071,907)	533,020,488 (364,019,692-782,075,347)	22,341,962 (17,674,941-29,067,991)	132,772,025 (105,469,666-159,980,920)	9,837,094 (7,060,215-13,379,267)	126,980,920 (94,820,783-161,796,126)
**Enterococcus faecium**	0.7	0.7	5 years	All age groups	UTI	54,099,502 (43,897,459-67,317,334)	4,642,824,741 (3,237,049,136-6,757,164,797)	417,190,016 (328,493,762-537,171,792)	1,584,051,621 (1,229,655,894-1,985,212,294)	158,733,464 (113,027,000-216,700,200)	1,411,175,532 (1,060,109,723-1,785,080,215)
**E. coli - non-diarrhogenic**	0.7	0.7	5 years	6 weeks & elderly age group	BSI	1,389,124 (1,213,582-1,598,303)	28,341,039 (22,634,504-36,177,519)	3,030,378 (2,567,565-3,631,487)	77,175,770 (68,327,424-86,933,884)	1,931,671 (1,639,382-2,355,285)	19,614,696 (16,293,221-23,426,548)
**E. coli - non-diarrhogenic**	0.7	0.7	5 years	All age groups	BSI	36,154,476 (31,885,739-41,662,923)	468,660,842 (380,749,265-586,558,519)	89,727,658 (77,843,819-104,710,688)	1,127,402,189 (1,004,206,169-1,274,850,856)	46,939,082 (39,593,545-56,932,301)	253,176,415 (209,788,397-300,676,715)
**ETEC**	0.6	0.7	5 years	6 months	BSI	0 (0-0)	0 (0-0)	0 (0-0)	0 (0-0)	0 (0-0)	0 (0-0)
**ExPEC - BSI**	0.7	0.7	5 years	6 weeks & elderly age group	BSI	1,389,124 (1,213,582-1,598,303)	28,341,039 (22,634,504-36,177,519)	3,030,378 (2,567,565-3,631,487)	77,175,770 (68,327,424-86,933,884)	1,931,671 (1,639,382-2,355,285)	19,614,696 (16,293,221-23,426,548)
**ExPEC - BSI**	0.7	0.7	5 years	All age groups	BSI	36,154,476 (31,885,739-41,662,923)	468,660,842 (380,749,265-586,558,519)	89,727,658 (77,843,819-104,710,688)	1,127,402,189 (1,004,206,169-1,274,850,856)	46,939,082 (39,593,545-56,932,301)	253,176,415 (209,788,397-300,676,715)
**ExPEC - UTI**	0.7	0.7	5 years	6 weeks & elderly age group	BSI	0 (0-0)	0 (0-0)	0 (0-0)	0 (0-0)	0 (0-0)	0 (0-0)
**ExPEC - UTI**	0.7	0.7	5 years	All age groups	BSI	0 (0-0)	0 (0-0)	0 (0-0)	0 (0-0)	0 (0-0)	0 (0-0)
**E. coli - non-diarrhogenic**	0.7	0.7	5 years	6 weeks & elderly age group	Bacterial skin infections	20,356,868 (17,402,985-23,549,481)	101,150,992 (81,892,301-122,669,584)	52,039,330 (42,393,755-62,443,294)	168,774,299 (141,753,597-199,378,762)	39,185,118 (29,620,956-51,413,298)	195,959,378 (159,219,747-236,285,329)
**E. coli - non-diarrhogenic**	0.7	0.7	5 years	All age groups	Bacterial skin infections	119,957,749 (102,516,484-140,117,287)	993,156,341 (808,383,201-1,211,684,532)	479,814,663 (392,513,898-580,678,122)	2,327,019,273 (1,932,091,486-2,769,321,013)	404,417,371 (303,326,990-535,088,016)	1,627,849,958 (1,320,600,660-1,946,120,569)
**ETEC**	0.6	0.7	5 years	6 months	Bacterial skin infections	0 (0-0)	0 (0-0)	0 (0-0)	0 (0-0)	0 (0-0)	0 (0-0)
**ExPEC - BSI**	0.7	0.7	5 years	6 weeks & elderly age group	Bacterial skin infections	0 (0-0)	0 (0-0)	0 (0-0)	0 (0-0)	0 (0-0)	0 (0-0)

**ExPEC - BSI**	0.7	0.7	5 years	All age groups	Bacterial skin infections	0 (0-0)	0 (0-0)	0 (0-0)	0 (0-0)	0 (0-0)	0 (0-0)
**ExPEC - UTI**	0.7	0.7	5 years	6 weeks & elderly age group	Bacterial skin infections	0 (0-0)	0 (0-0)	0 (0-0)	0 (0-0)	0 (0-0)	0 (0-0)
**ExPEC - UTI**	0.7	0.7	5 years	All age groups	Bacterial skin infections	0 (0-0)	0 (0-0)	0 (0-0)	0 (0-0)	0 (0-0)	0 (0-0)
**E. coli - non-diarrhogenic**	0.7	0.7	5 years	6 weeks & elderly age group	Bone and joint infections	14,579.45 (12,788.43-16,657.88)	357,922.5 (259,387.6-508,727.9)	45,449.68 (36,365.97-57,611.86)	427,215.4 (327,749.7-543,854.7)	74,468.18 (58,800.3-93,538.92)	506,224.2 (376,547.6-687,939.2)
**E. coli - non-diarrhogenic**	0.7	0.7	5 years	All age groups	Bone and joint infections	111,437.5 (96,209.29-127,231.9)	3,696,205 (2,616,929-5,184,559)	572,343.3 (450,337.5-733,422.6)	4,379,030 (3,374,148-5,601,981)	576,637.1 (463,811.7-722,765.2)	4,469,257 (3,346,750-6,118,442)
**ETEC**	0.6	0.7	5 years	6 months	Bone and joint infections	0 (0-0)	0 (0-0)	0 (0-0)	0 (0-0)	0 (0-0)	0 (0-0)
**ExPEC - BSI**	0.7	0.7	5 years	6 weeks & elderly age group	Bone and joint infections	0 (0-0)	0 (0-0)	0 (0-0)	0 (0-0)	0 (0-0)	0 (0-0)
**ExPEC - BSI**	0.7	0.7	5 years	All age groups	Bone and joint infections	0 (0-0)	0 (0-0)	0 (0-0)	0 (0-0)	0 (0-0)	0 (0-0)
**ExPEC - UTI**	0.7	0.7	5 years	6 weeks & elderly age group	Bone and joint infections	0 (0-0)	0 (0-0)	0 (0-0)	0 (0-0)	0 (0-0)	0 (0-0)
**ExPEC - UTI**	0.7	0.7	5 years	All age groups	Bone and joint infections	0 (0-0)	0 (0-0)	0 (0-0)	0 (0-0)	0 (0-0)	0 (0-0)
**E. coli - non-diarrhogenic**	0.7	0.7	5 years	6 weeks & elderly age group	CNS infections	3,793,441 (3,225,316-4,463,807)	1,909,565 (1,587,225-2,335,953)	3,077,229 (2,550,849-3,686,065)	2,663,937 (2,358,843-3,015,283)	3,220,039 (2,503,305-4,294,860)	1,278,435 (1,105,929-1,510,943)
**E. coli - non-diarrhogenic**	0.7	0.7	5 years	All age groups	CNS infections	5,706,627 (4,917,280-6,668,772)	7,199,166 (5,640,360-9,199,076)	6,565,171 (5,448,462-8,057,772)	8,188,841 (7,220,327-9,202,038)	6,583,571 (5,108,719-8,799,014)	4,523,974 (3,848,880-5,365,040)
**ETEC**	0.6	0.7	5 years	6 months	CNS infections	0 (0-0)	0 (0-0)	0 (0-0)	0 (0-0)	0 (0-0)	0 (0-0)
**ExPEC - BSI**	0.7	0.7	5 years	6 weeks & elderly age group	CNS infections	0 (0-0)	0 (0-0)	0 (0-0)	0 (0-0)	0 (0-0)	0 (0-0)
**ExPEC - BSI**	0.7	0.7	5 years	All age groups	CNS infections	0 (0-0)	0 (0-0)	0 (0-0)	0 (0-0)	0 (0-0)	0 (0-0)
**ExPEC - UTI**	0.7	0.7	5 years	6 weeks & elderly age group	CNS infections	0 (0-0)	0 (0-0)	0 (0-0)	0 (0-0)	0 (0-0)	0 (0-0)
**ExPEC - UTI**	0.7	0.7	5 years	All age groups	CNS infections	0 (0-0)	0 (0-0)	0 (0-0)	0 (0-0)	0 (0-0)	0 (0-0)

**E. coli - non-diarrhogenic**	0.7	0.7	5 years	6 weeks & elderly age group	Cardiac infections	182,729.7 (152,371.8-225,701.3)	12,947,573 (9,448,550-17,443,571)	673,367 (544,797.7-851,314.2)	25,370,589 (22,315,365-28,502,181)	264,253.3 (221,096.9-322,204.8)	5,126,694 (4,242,306-6,277,608)
**E. coli - non-diarrhogenic**	0.7	0.7	5 years	All age groups	Cardiac infections	1,091,259 (902,128.5-1,328,262)	91,862,041 (67,187,048-123,662,917)	5,862,418 (4,789,002-7,379,266)	193,929,812 (171,783,466-217,468,227)	2,277,891 (1,903,842-2,803,376)	34,549,851 (28,569,304-42,007,139)
**ETEC**	0.6	0.7	5 years	6 months	Cardiac infections	0 (0-0)	0 (0-0)	0 (0-0)	0 (0-0)	0 (0-0)	0 (0-0)
**ExPEC - BSI**	0.7	0.7	5 years	6 weeks & elderly age group	Cardiac infections	0 (0-0)	0 (0-0)	0 (0-0)	0 (0-0)	0 (0-0)	0 (0-0)
**ExPEC - BSI**	0.7	0.7	5 years	All age groups	Cardiac infections	0 (0-0)	0 (0-0)	0 (0-0)	0 (0-0)	0 (0-0)	0 (0-0)
**ExPEC - UTI**	0.7	0.7	5 years	6 weeks & elderly age group	Cardiac infections	0 (0-0)	0 (0-0)	0 (0-0)	0 (0-0)	0 (0-0)	0 (0-0)
**ExPEC - UTI**	0.7	0.7	5 years	All age groups	Cardiac infections	0 (0-0)	0 (0-0)	0 (0-0)	0 (0-0)	0 (0-0)	0 (0-0)
**E. coli - non-diarrhogenic**	0.7	0.7	5 years	6 weeks & elderly age group	Diarrhoea	0 (0-0)	0 (0-0)	0 (0-0)	0 (0-0)	0 (0-0)	0 (0-0)
**E. coli - non-diarrhogenic**	0.7	0.7	5 years	All age groups	Diarrhoea	0 (0-0)	0 (0-0)	0 (0-0)	0 (0-0)	0 (0-0)	0 (0-0)
**ETEC**	0.6	0.7	5 years	6 months	Diarrhoea	93,520,700 (74,560,586-118,771,162)	231,363,633 (166,845,931-335,554,363)	456,189,508 (340,322,304-602,927,225)	433,209,199 (334,929,599-556,818,372)	187,425,647 (143,587,906-249,985,920)	47,980,261 (37,887,318-63,043,068)
**ExPEC - BSI**	0.7	0.7	5 years	6 weeks & elderly age group	Diarrhoea	0 (0-0)	0 (0-0)	0 (0-0)	0 (0-0)	0 (0-0)	0 (0-0)
**ExPEC - BSI**	0.7	0.7	5 years	All age groups	Diarrhoea	0 (0-0)	0 (0-0)	0 (0-0)	0 (0-0)	0 (0-0)	0 (0-0)
**ExPEC - UTI**	0.7	0.7	5 years	6 weeks & elderly age group	Diarrhoea	0 (0-0)	0 (0-0)	0 (0-0)	0 (0-0)	0 (0-0)	0 (0-0)
**ExPEC - UTI**	0.7	0.7	5 years	All age groups	Diarrhoea	0 (0-0)	0 (0-0)	0 (0-0)	0 (0-0)	0 (0-0)	0 (0-0)
**E. coli - non-diarrhogenic**	0.7	0.7	5 years	6 weeks & elderly age group	Intra-abdominal infections	1,239,984 (1,083,264-1,416,158)	21,597,623 (17,091,464-27,282,194)	4,301,488 (3,541,648-5,211,819)	24,814,304 (21,678,162-28,814,494)	6,138,208 (5,070,962-7,720,954)	24,092,136 (20,194,606-29,416,645)
**E. coli - non-diarrhogenic**	0.7	0.7	5 years	All age groups	Intra-abdominal infections	12,963,422 (11,522,082-14,744,718)	227,480,851 (178,756,857-285,185,755)	45,425,688 (37,851,692-54,393,476)	269,151,051 (231,864,844-309,670,517)	74,755,969 (60,765,247-94,113,229)	225,959,714 (189,493,216-275,413,469)
**ETEC**	0.6	0.7	5 years	6 months	Intra-abdominal infections	0 (0-0)	0 (0-0)	0 (0-0)	0 (0-0)	0 (0-0)	0 (0-0)
**ExPEC - BSI**	0.7	0.7	5 years	6 weeks & elderly age group	Intra-abdominal infections	0 (0-0)	0 (0-0)	0 (0-0)	0 (0-0)	0 (0-0)	0 (0-0)

**ExPEC - BSI**	0.7	0.7	5 years	All age groups	Intra-abdominal infections	0 (0-0)	0 (0-0)	0 (0-0)	0 (0-0)	0 (0-0)	0 (0-0)
**ExPEC - UTI**	0.7	0.7	5 years	6 weeks & elderly age group	Intra-abdominal infections	0 (0-0)	0 (0-0)	0 (0-0)	0 (0-0)	0 (0-0)	0 (0-0)
**ExPEC - UTI**	0.7	0.7	5 years	All age groups	Intra-abdominal infections	0 (0-0)	0 (0-0)	0 (0-0)	0 (0-0)	0 (0-0)	0 (0-0)
**E. coli - non-diarrhogenic**	0.7	0.7	5 years	6 weeks & elderly age group	LRI and thorax infections	2,480,424 (1,954,001-3,131,320)	26,223,559 (19,387,566-34,642,694)	7,766,794 (6,298,676-9,648,661)	26,566,232 (21,419,672-31,770,184)	12,876,161 (9,447,014-17,400,768)	18,619,036 (14,605,104-23,895,474)
**E. coli - non-diarrhogenic**	0.7	0.7	5 years	All age groups	LRI and thorax infections	13,510,556 (10,544,757-17,022,675)	200,163,628 (146,266,279-272,621,969)	51,413,116 (41,722,912-66,444,915)	197,573,557 (159,870,821-237,462,559)	118,590,683 (85,437,468-162,112,131)	142,521,230 (110,464,069-181,946,531)
**ETEC**	0.6	0.7	5 years	6 months	LRI and thorax infections	0 (0-0)	0 (0-0)	0 (0-0)	0 (0-0)	0 (0-0)	0 (0-0)
**ExPEC - BSI**	0.7	0.7	5 years	6 weeks & elderly age group	LRI and thorax infections	0 (0-0)	0 (0-0)	0 (0-0)	0 (0-0)	0 (0-0)	0 (0-0)
**ExPEC - BSI**	0.7	0.7	5 years	All age groups	LRI and thorax infections	0 (0-0)	0 (0-0)	0 (0-0)	0 (0-0)	0 (0-0)	0 (0-0)
**ExPEC - UTI**	0.7	0.7	5 years	6 weeks & elderly age group	LRI and thorax infections	0 (0-0)	0 (0-0)	0 (0-0)	0 (0-0)	0 (0-0)	0 (0-0)
**ExPEC - UTI**	0.7	0.7	5 years	All age groups	LRI and thorax infections	0 (0-0)	0 (0-0)	0 (0-0)	0 (0-0)	0 (0-0)	0 (0-0)
**E. coli - non-diarrhogenic**	0.7	0.7	5 years	6 weeks & elderly age group	UTI	40,239,951 (32,873,634-49,221,486)	2,829,024,067 (2,031,893,217-3,858,530,442)	302,788,813 (241,925,787-392,291,579)	887,183,941 (719,014,819-1,087,825,140)	149,701,904 (109,147,674-200,611,261)	1,487,188,914 (1,128,194,943-1,925,219,372)
**E. coli - non-diarrhogenic**	0.7	0.7	5 years	All age groups	UTI	734,087,592 (604,171,820-888,638,706)	31,109,500,677 (22,373,071,891-41,993,382,128)	5,716,781,602 (4,553,597,450-7,365,108,658)	16,067,591,870 (12,815,383,811-20,148,742,103)	5,050,554,160 (3,681,903,401-6,714,898,027)	19,918,361,606 (15,410,083,887-24,461,540,505)
**ETEC**	0.6	0.7	5 years	6 months	UTI	0 (0-0)	0 (0-0)	0 (0-0)	0 (0-0)	0 (0-0)	0 (0-0)
**ExPEC - BSI**	0.7	0.7	5 years	6 weeks & elderly age group	UTI	0 (0-0)	0 (0-0)	0 (0-0)	0 (0-0)	0 (0-0)	0 (0-0)
**ExPEC - BSI**	0.7	0.7	5 years	All age groups	UTI	0 (0-0)	0 (0-0)	0 (0-0)	0 (0-0)	0 (0-0)	0 (0-0)
**ExPEC - UTI**	0.7	0.7	5 years	6 weeks & elderly age group	UTI	40,239,951 (32,873,634-49,221,486)	2,829,024,067 (2,031,893,217-3,858,530,442)	302,788,813 (241,925,787-392,291,579)	887,183,941 (719,014,819-1,087,825,140)	149,701,904 (109,147,674-200,611,261)	1,487,188,914 (1,128,194,943-1,925,219,372)

**ExPEC - UTI**	0.7	0.7	5 years	All age groups	UTI	734,087,592 (604,171,820-888,638,706)	31,109,500,677 (22,373,071,891-41,993,382,128)	5,716,781,602 (4,553,597,450-7,365,108,658)	16,067,591,870 (12,815,383,811-20,148,742,103)	5,050,554,160 (3,681,903,401-6,714,898,027)	19,918,361,606 (15,410,083,887-24,461,540,505)
**Group A streptococcus**	0.7	0.7	5 years	6 weeks	BSI	376,807.7 (291,207.4-487,183.6)	2,581,864 (1,428,826-4,895,643)	391,673.2 (304,157.5-506,152.1)	1,032,034 (844,228.2-1,274,963)	136,634 (88,908.12-203,091.8)	2,395,723 (1,240,196-4,266,748)
**Group A streptococcus**	0.7	0.7	5 years	6 weeks	Bacterial skin infections	68,016,846 (53,289,551-86,239,413)	402,665,015 (249,036,806-710,989,187)	106,107,492 (81,284,810-136,275,514)	450,120,428 (364,283,023-561,843,106)	47,266,612 (29,402,852-80,213,534)	1,791,575,229 (1,031,910,556-2,904,691,116)
**Group A streptococcus**	0.7	0.7	5 years	6 weeks	Bone and joint infections	1,904.289 (1,455.696-2,505.436)	4,946.599 (2,611.113-9,698.391)	1,841.779 (1,381.044-2,746.812)	982.468 (816.3336-1,224.961)	789.7462 (501.0167-1,248.643)	7,777.804 (3,896.689-15,629.94)
**Group A streptococcus**	0.7	0.7	5 years	6 weeks	Cardiac infections	22,969.67 (17,385.61-30,342.77)	328,039.2 (159,810.3-698,490.5)	58,202.24 (44,601.44-75,898.14)	155,913.9 (123,675.9-194,737.5)	11,251.91 (7,026.066-16,877.54)	319,031.7 (164,037.6-566,432.9)
**Haemophilus influenzae type B**	0.93	0.9	5 years	6, 10, 14 weeks	CNS infections	202,987.8 (154,209.3-280,421.5)	34,052.2 (24,628.83-48,005.16)	182,958.4 (114,773.5-312,719.1)	78,163.37 (61,428.83-99,429.06)	62,716.77 (35,677.48-110,920.9)	42,786.16 (31,857.24-57,339.85)
**Haemophilus influenzae type B**	both	0.9	5 years	6, 10, 14 weeks	CNS infections	202,987.8 (154,209.3-280,421.5)	34,052.2 (24,628.83-48,005.16)	182,958.4 (114,773.5-312,719.1)	78,163.37 (61,428.83-99,429.06)	62,716.77 (35,677.48-110,920.9)	42,786.16 (31,857.24-57,339.85)
**Haemophilus influenzae type B**	current	current	5 years	6, 10, 14 weeks	CNS infections	164,949.1 (125,311.1-227,871.2)	32,441.66 (23,452.31-45,736.25)	172,821.2 (108,443.8-296,021.3)	80,973.41 (63,637.3-103,003.5)	62,998.37 (35,836.99-111,416.8)	40,406.96 (30,076.41-54,125.71)
**Haemophilus influenzae type B**	0.69	0.9	5 years	6, 10, 14 weeks	LRI and thorax infections	353,340 (275,713.3-447,410.4)	1,959,835 (1,186,986-3,438,239)	1,111,112 (819,834-1,523,594)	1,425,413 (1,162,509-1,756,113)	368,819.6 (235,029.9-620,085)	609,276.1 (446,143.8-817,332)
**Haemophilus influenzae type B**	both	0.9	5 years	6, 10, 14 weeks	LRI and thorax infections	353,340 (275,713.3-447,410.4)	1,959,835 (1,186,986-3,438,239)	1,111,112 (819,834-1,523,594)	1,425,413 (1,162,509-1,756,113)	368,819.6 (235,029.9-620,085)	609,276.1 (446,143.8-817,332)
**Haemophilus influenzae type B**	current	current	5 years	6, 10, 14 weeks	LRI and thorax infections	287,388.6 (224,246.6-363,913)	1,878,565 (1,137,836-3,297,620)	1,040,025 (767,173.7-1,425,285)	1,481,722 (1,208,449-1,825,789)	370,186 (235,883.1-622,374.2)	571,899.5 (418,763.6-767,118.4)
**Klebsiella pneumoniae - BSI**	0.7	0.7	6 months	0 weeks (maternal)	BSI	33,714,617 (30,281,904-37,676,549)	47,511,700 (41,890,350-54,688,573)	46,067,002 (40,073,996-54,143,451)	73,247,969 (62,367,278-88,018,946)	26,830,530 (22,374,582-33,135,137)	38,639,966 (29,875,007-50,171,250)
**Klebsiella pneumoniae - all**	0.7	0.7	5 years	6 weeks & elderly age group	BSI	4,926,917 (4,372,384-5,523,801)	15,637,411 (13,474,109-18,753,962)	5,362,210 (4,688,919-6,335,321)	28,007,401 (24,593,998-31,792,872)	3,483,786 (2,897,412-4,288,267)	6,466,833 (5,304,048-7,875,942)
**Klebsiella pneumoniae - all**	0.7	0.7	5 years	All age groups	BSI	81,859,862 (73,140,572-91,988,701)	266,238,250 (231,920,240-317,381,589)	120,583,626 (105,296,431-141,002,842)	522,834,563 (454,930,293-611,962,422)	80,897,396 (67,430,999-100,227,140)	106,650,918 (86,091,065-132,069,258)
**Klebsiella pneumoniae - BSI**	0.7	0.7	6 months	0 weeks (maternal)	Bacterial skin infections	0 (0-0)	0 (0-0)	0 (0-0)	0 (0-0)	0 (0-0)	0 (0-0)

**Klebsiella pneumoniae - all**	0.7	0.7	5 years	6 weeks & elderly age group	Bacterial skin infections	10,560,074 (9,233,987-12,241,301)	30,306,840 (25,832,143-35,469,959)	19,437,182 (16,184,620-23,350,918)	81,011,437 (70,160,320-93,100,789)	19,024,031 (14,175,150-25,166,299)	34,062,800 (28,201,143-41,175,810)
**Klebsiella pneumoniae - all**	0.7	0.7	5 years	All age groups	Bacterial skin infections	66,045,670 (57,637,175-77,390,659)	286,000,033 (244,024,165-335,231,408)	181,680,195 (149,672,093-217,209,888)	970,360,823 (831,975,449-1,113,043,539)	185,299,908 (136,603,963-246,228,446)	271,237,039 (225,081,774-327,257,908)
**Klebsiella pneumoniae - BSI**	0.7	0.7	6 months	0 weeks (maternal)	Bone and joint infections	0 (0-0)	0 (0-0)	0 (0-0)	0 (0-0)	0 (0-0)	0 (0-0)
**Klebsiella pneumoniae - all**	0.7	0.7	5 years	6 weeks & elderly age group	Bone and joint infections	9,569.647 (8,412.247-10,799.31)	121,109.5 (94,089.05-154,322.6)	21,904.5 (18,097.36-27,227.57)	226,011.9 (183,967.4-282,236.2)	42,815.6 (33,220.56-55,483.32)	103,017.2 (78,608.04-139,679.6)
**Klebsiella pneumoniae - all**	0.7	0.7	5 years	All age groups	Bone and joint infections	156,152.9 (136,694.6-178,108.4)	1,972,151 (1,541,375-2,478,254)	527,255 (424,779.4-659,997)	4,099,315 (3,285,291-5,142,312)	508,397.9 (404,704.1-648,128.4)	1,630,034 (1,245,614-2,228,969)
**Klebsiella pneumoniae - BSI**	0.7	0.7	6 months	0 weeks (maternal)	CNS infections	0 (0-0)	0 (0-0)	0 (0-0)	0 (0-0)	0 (0-0)	0 (0-0)
**Klebsiella pneumoniae - all**	0.7	0.7	5 years	6 weeks & elderly age group	CNS infections	3,801,163 (3,275,208-4,494,228)	980,160.8 (831,282.2-1,143,622)	2,427,404 (2,002,990-2,880,733)	2,427,948 (2,123,023-2,787,860)	2,792,732 (2,154,849-3,784,088)	623,105.9 (529,752.1-749,443.4)
**Klebsiella pneumoniae - all**	0.7	0.7	5 years	All age groups	CNS infections	6,415,376 (5,580,646-7,409,781)	3,961,798 (3,309,497-4,764,550)	5,986,013 (4,944,303-7,345,822)	9,012,691 (7,931,443-10,344,552)	6,739,290 (5,211,248-9,126,781)	2,201,534 (1,871,892-2,651,823)
**Klebsiella pneumoniae - BSI**	0.7	0.7	6 months	0 weeks (maternal)	Cardiac infections	0 (0-0)	0 (0-0)	0 (0-0)	0 (0-0)	0 (0-0)	0 (0-0)
**Klebsiella pneumoniae - all**	0.7	0.7	5 years	6 weeks & elderly age group	Cardiac infections	557,146.1 (479,533.7-662,892.9)	4,764,378 (3,777,291-6,008,981)	1,309,245 (1,065,486-1,621,467)	7,147,306 (6,355,380-8,000,960)	474,702.7 (386,172-601,065)	1,542,862 (1,219,179-1,931,113)
**Klebsiella pneumoniae - all**	0.7	0.7	5 years	All age groups	Cardiac infections	1,867,681 (1,635,170-2,217,251)	32,854,338 (25,810,229-41,785,257)	6,262,844 (5,099,704-7,771,357)	61,757,995 (54,666,345-69,527,445)	3,366,829 (2,753,428-4,265,647)	9,965,171 (8,041,765-12,263,339)
**Klebsiella pneumoniae - BSI**	0.7	0.7	6 months	0 weeks (maternal)	Intra-abdominal infections	0 (0-0)	0 (0-0)	0 (0-0)	0 (0-0)	0 (0-0)	0 (0-0)
**Klebsiella pneumoniae - all**	0.7	0.7	5 years	6 weeks & elderly age group	Intra-abdominal infections	839,035.7 (738,939.2-951,145.9)	10,037,994 (8,212,487-12,583,793)	2,230,042 (1,842,962-2,662,538)	15,881,933 (13,950,857-18,420,633)	3,837,207 (2,968,292-4,939,601)	5,766,767 (4,593,591-7,180,028)
**Klebsiella pneumoniae - all**	0.7	0.7	5 years	All age groups	Intra-abdominal infections	8,472,836 (7,454,942-9,621,028)	95,490,088 (77,894,281-119,103,079)	20,989,849 (17,275,188-24,827,614)	179,873,841 (157,558,905-213,001,849)	38,465,252 (29,674,064-49,435,141)	56,350,761 (45,498,588-69,786,271)
**Klebsiella pneumoniae - BSI**	0.7	0.7	6 months	0 weeks (maternal)	LRI and thorax infections	0 (0-0)	0 (0-0)	0 (0-0)	0 (0-0)	0 (0-0)	0 (0-0)
**Klebsiella pneumoniae - all**	0.7	0.7	5 years	6 weeks & elderly age group	LRI and thorax infections	6,504,466 (5,231,042-8,123,867)	45,958,552 (36,762,828-57,043,621)	18,207,081 (14,931,349-22,625,361)	75,295,709 (63,605,906-89,903,732)	31,467,616 (22,072,863-45,574,587)	23,553,287 (18,446,007-30,658,692)

**Klebsiella pneumoniae - all**	0.7	0.7	5 years	All age groups	LRI and thorax infections	32,573,076 (26,121,050-40,300,648)	309,490,461 (246,621,887-390,046,563)	112,109,978 (91,414,856-140,159,662)	551,999,029 (457,346,407-667,816,365)	257,862,290 (179,673,540-376,191,589)	163,556,736 (126,804,042-214,472,279)
**Klebsiella pneumoniae - BSI**	0.7	0.7	6 months	0 weeks (maternal)	UTI	0 (0-0)	0 (0-0)	0 (0-0)	0 (0-0)	0 (0-0)	0 (0-0)
**Klebsiella pneumoniae - all**	0.7	0.7	5 years	6 weeks & elderly age group	UTI	9,925,869 (8,370,223-11,930,112)	435,524,246 (327,606,659-590,961,269)	60,697,624 (49,293,151-78,068,405)	383,679,459 (319,338,950-464,844,299)	46,040,209 (33,724,559-62,416,832)	130,709,313 (106,165,877-158,924,207)
**Klebsiella pneumoniae - all**	0.7	0.7	5 years	All age groups	UTI	171,930,821 (146,007,653-205,920,873)	4,494,058,783 (3,445,628,614-5,957,943,849)	1,086,652,463 (873,464,486-1,393,669,004)	4,859,284,937 (3,987,218,248-5,878,473,171)	1,083,116,133 (797,882,978-1,472,629,683)	1,895,299,606 (1,535,001,434-2,351,489,141)
**Mycobacterium tuberculosis - Improved**	0.8	0.7	10 years	0 weeks + boost every 10 years	TB	16,746,638 (13,721,280-20,932,078)	34,654,518 (27,726,980-43,232,514)	26,095,711 (20,386,479-33,604,752)	729,912,543 (567,087,388-931,261,153)	112,682,640 (66,217,116-179,351,193)	9,889,736 (7,141,432-13,406,521)
**Mycobacterium tuberculosis - M72**	0.5	0.7	10 years	10 years + boost every 10 years	TB	9,070,063 (7,420,383-11,334,984)	20,880,975 (16,692,281-25,988,986)	15,028,460 (11,769,442-19,184,029)	447,382,550 (347,463,967-570,983,492)	68,923,378 (40,459,144-109,755,396)	6,092,872 (4,363,260-8,271,210)
**Non-typhoidal Salmonella**	0.8	0.7	5 years	6 weeks & 9 months	BSI	12,741.33 (10,692.84-15,214.08)	21,705.08 (17,922.2-26,097.98)	24,571.48 (19,082.58-31,645.23)	47,673.21 (40,023.62-58,386.23)	295,631.4 (218,957.4-400,765.8)	223,442.1 (160,915.2-302,644.7)
**Non-typhoidal Salmonella**	0.8	0.7	5 years	6 weeks & 9 months	Cardiac infections	767.7051 (644.728-900.7309)	1,277.799 (1,042.537-1,542.441)	1,702.618 (1,471.741-1,963.662)	6,007.515 (5,250.219-7,094.347)	21,675.61 (16,565.36-28,744.1)	28,886.09 (20,466.46-39,395.85)
**Non-typhoidal Salmonella**	0.8	0.7	5 years	6 weeks & 9 months	Diarrhoea	11,187,397 (9,099,725-14,073,651)	32,873,271 (28,045,979-39,356,574)	28,628,424 (24,623,162-34,075,399)	457,095,586 (381,154,487-570,441,536)	126,713,614 (98,662,956-156,831,155)	769,380,566 (541,733,194-1,053,142,056)
**Non-typhoidal Salmonella**	0.8	0.7	5 years	6 weeks & 9 months	Typhoid, paratyphoid, and iNTS	50,170.09 (40,597.24-63,426.09)	10,391.67 (8,562.091-12,890.36)	26,263.95 (21,523.54-32,883.74)	243,316.3 (178,570.9-333,698.5)	253,326 (194,799.4-319,724.7)	93,126.8 (69,846.6-119,959)
**Pseudomonas aeruginosa**	0.7	0.7	5 years	6 weeks & elderly age group	BSI	1,106,692 (935,858.2-1,306,810)	15,554,403 (12,612,987-19,109,098)	1,915,436 (1,608,770-2,316,026)	9,236,459 (8,213,140-10,294,645)	1,105,675 (919,375.1-1,359,645)	4,019,280 (3,371,174-4,802,521)
**Pseudomonas aeruginosa**	0.7	0.7	5 years	All age groups	BSI	42,994,389 (36,521,853-50,851,787)	400,554,826 (333,520,751-486,914,371)	84,502,823 (72,667,896-100,159,215)	233,522,673 (203,880,331-270,009,142)	45,276,466 (37,627,501-55,800,468)	93,582,922 (75,464,814-113,028,279)
**Pseudomonas aeruginosa**	0.7	0.7	5 years	6 weeks & elderly age group	Bacterial skin infections	0 (0-0)	0 (0-0)	0 (0-0)	0 (0-0)	0 (0-0)	0 (0-0)
**Pseudomonas aeruginosa**	0.7	0.7	5 years	All age groups	Bacterial skin infections	0 (0-0)	0 (0-0)	0 (0-0)	0 (0-0)	0 (0-0)	0 (0-0)
**Pseudomonas aeruginosa**	0.7	0.7	5 years	6 weeks & elderly age group	Bone and joint infections	0 (0-0)	0 (0-0)	0 (0-0)	0 (0-0)	0 (0-0)	0 (0-0)
**Pseudomonas aeruginosa**	0.7	0.7	5 years	All age groups	Bone and joint infections	0 (0-0)	0 (0-0)	0 (0-0)	0 (0-0)	0 (0-0)	0 (0-0)

**Pseudomonas aeruginosa**	0.7	0.7	5 years	6 weeks & elderly age group	Cardiac infections	0 (0-0)	0 (0-0)	0 (0-0)	0 (0-0)	0 (0-0)	0 (0-0)
**Pseudomonas aeruginosa**	0.7	0.7	5 years	All age groups	Cardiac infections	0 (0-0)	0 (0-0)	0 (0-0)	0 (0-0)	0 (0-0)	0 (0-0)
**Pseudomonas aeruginosa**	0.7	0.7	5 years	6 weeks & elderly age group	Intra-abdominal infections	0 (0-0)	0 (0-0)	0 (0-0)	0 (0-0)	0 (0-0)	0 (0-0)
**Pseudomonas aeruginosa**	0.7	0.7	5 years	All age groups	Intra-abdominal infections	0 (0-0)	0 (0-0)	0 (0-0)	0 (0-0)	0 (0-0)	0 (0-0)
**Pseudomonas aeruginosa**	0.7	0.7	5 years	6 weeks & elderly age group	LRI and thorax infections	6,373,770 (5,238,172-7,708,288)	176,957,750 (131,992,864-239,642,205)	20,759,233 (17,613,532-25,244,049)	151,542,439 (131,365,759-174,975,123)	21,875,756 (15,428,480-32,371,539)	43,651,318 (33,753,536-56,454,336)
**Pseudomonas aeruginosa**	0.7	0.7	5 years	All age groups	LRI and thorax infections	39,281,907 (31,490,482-47,607,207)	1,524,543,083 (1,102,368,009-2,119,508,051)	165,644,373 (138,432,009-206,368,951)	1,196,019,848 (1,029,636,133-1,398,636,890)	223,560,946 (153,087,024-334,976,141)	355,532,954 (274,066,253-465,827,521)
**Pseudomonas aeruginosa**	0.7	0.7	5 years	6 weeks & elderly age group	UTI	0 (0-0)	0 (0-0)	0 (0-0)	0 (0-0)	0 (0-0)	0 (0-0)
**Pseudomonas aeruginosa**	0.7	0.7	5 years	All age groups	UTI	0 (0-0)	0 (0-0)	0 (0-0)	0 (0-0)	0 (0-0)	0 (0-0)
**Salmonella paratyphi**	0.7	0.7	5 years	9 months	Typhoid, paratyphoid, and iNTS	44,802.31 (37,140.27-54,069.6)	79,924.25 (61,475.65-103,610.9)	773,192.3 (591,742.3-1,027,796)	129,108.3 (110,394.6-151,512.2)	4,894,523 (3,779,395-6,554,079)	427,368.4 (335,272.8-545,480.6)
**Salmonella Typhi**	0.85	0.7	15 years	9 months	BSI	1,182,433 (1,044,269-1,337,097)	275,320.7 (239,783.2-312,338.7)	478,973.5 (408,115.9-567,361.5)	116,273.3 (106,327.7-128,547.2)	203,055.1 (163,909.2-258,461.8)	51,937.1 (46,213.41-58,463.57)
**Salmonella Typhi**	0.85	0.7	15 years	9 months	Cardiac infections	98,562.03 (85,441.75-113,915.1)	26,461.69 (22,569.54-30,545.57)	45,470.52 (41,004.44-51,150.68)	21,022.03 (18,857.18-23,169.42)	22,554.99 (17,738.31-29,624.03)	7,275.486 (6,376.796-8,363.507)
**Salmonella Typhi**	0.85	0.7	15 years	9 months	Typhoid, paratyphoid, and iNTS	13,918,573 (11,926,672-16,107,333)	332,540.5 (266,579.9-422,296.9)	19,769,279 (16,481,400-24,365,535)	454,319.3 (372,714.4-572,551.5)	67,076,417 (52,409,247-89,814,268)	4,230,263 (3,711,324-4,851,819)
**Shigella**	0.6	0.7	5 years	6 months	Diarrhoea	71,997,021 (48,229,193-109,799,367)	255,670,942 (181,891,634-379,719,692)	424,213,367 (276,031,634-634,603,388)	222,164,705 (164,741,085-302,412,183)	124,701,567 (79,962,659-192,754,130)	275,450,129 (167,123,091-429,694,132)
**Staphylococcus aureus**	0.6	0.7	5 years	6 weeks & elderly age group	BSI	2,369,609 (2,073,376-2,739,327)	70,736,419 (54,850,513-91,734,133)	4,534,135 (3,960,999-5,188,518)	37,777,823 (33,580,498-42,186,826)	2,478,674 (2,092,952-3,050,518)	37,440,176 (31,023,532-45,192,647)
**Staphylococcus aureus**	0.6	0.7	5 years	All age groups	BSI	69,326,373 (60,288,040-79,943,541)	1,659,049,951 (1,295,233,543-2,124,806,941)	167,717,287 (148,360,587-191,765,161)	798,400,423 (716,581,456-882,930,615)	85,951,960 (72,309,228-106,494,356)	677,397,745 (550,472,955-812,663,367)
**Staphylococcus aureus**	0.6	0.7	5 years	6 weeks & elderly age group	Bacterial skin infections	426,559,814 (361,443,104-503,189,533)	1,348,500,626 (1,114,387,153-1,669,519,252)	734,748,894 (619,721,111-873,913,438)	1,279,235,776 (1,083,948,748-1,502,543,335)	520,866,787 (376,856,794-708,207,802)	2,020,584,167 (1,643,420,623-2,485,410,570)
**Staphylococcus aureus**	0.6	0.7	5 years	All age groups	Bacterial skin infections	1,390,061,978 (1,177,790,182-	7,851,009,092 (6,476,929,620-9,810,718,800)	3,364,524,959 (2,849,583,101-	10,658,925,167 (8,953,109,000-	2,844,371,987 (2,046,926,468-	9,143,602,276 (7,390,766,361-

						1,664,611,065)		3,995,568,971)	12,704,700,233)	3,900,125,587)	11,077,580,213)
**Staphylococcus aureus**	0.6	0.7	5 years	6 weeks & elderly age group	Bone and joint infections	55,798.65 (46,071.97-67,681.8)	3,183,584 (2,166,016-4,537,123)	263,935.8 (194,538.4-355,725)	2,087,284 (1,614,426-2,790,732)	192,671.9 (152,720-248,091.9)	2,831,785 (1,907,169-4,048,355)
**Staphylococcus aureus**	0.6	0.7	5 years	All age groups	Bone and joint infections	867,625.1 (700,406.6-1,090,559)	46,844,324 (32,619,328-66,105,895)	5,498,187 (4,125,760-7,467,723)	29,830,785 (23,073,809-38,566,048)	2,272,042 (1,840,941-2,850,644)	36,059,795 (23,589,832-50,840,518)
**Staphylococcus aureus**	0.6	0.7	5 years	6 weeks & elderly age group	CNS infections	3,019,909 (2,565,533-3,568,545)	2,846,027 (2,234,598-3,650,863)	2,755,357 (2,292,171-3,306,541)	2,315,764 (2,071,581-2,593,211)	2,248,375 (1,688,187-3,030,031)	1,239,202 (1,053,617-1,454,153)
**Staphylococcus aureus**	0.6	0.7	5 years	All age groups	CNS infections	6,411,467 (5,560,253-7,438,247)	19,441,336 (14,687,544-25,458,502)	8,802,098 (7,399,068-10,773,691)	11,717,043 (10,428,808-13,035,149)	7,187,338 (5,424,188-9,669,135)	7,865,804 (6,755,247-9,420,388)
**Staphylococcus aureus**	0.6	0.7	5 years	6 weeks & elderly age group	Cardiac infections	364,132.8 (294,879.6-450,979.4)	34,621,881 (24,587,760-47,333,741)	1,596,299 (1,285,492-2,002,241)	13,180,950 (11,585,051-14,881,842)	379,879.4 (312,874.3-469,262.2)	9,967,766 (8,216,549-12,245,681)
**Staphylococcus aureus**	0.6	0.7	5 years	All age groups	Cardiac infections	2,300,333 (1,876,758-2,806,352)	301,336,347 (214,537,184-412,088,500)	13,869,367 (11,307,411-17,437,312)	118,735,632 (105,234,017-133,238,959)	4,122,726 (3,392,653-5,109,636)	81,293,059 (67,292,871-99,202,295)
**Staphylococcus aureus**	0.6	0.7	5 years	6 weeks & elderly age group	Intra-abdominal infections	339,586.5 (292,816.2-395,066.5)	14,841,340 (11,731,490-19,338,163)	906,215.3 (771,947.4-1,071,689)	8,828,039 (7,570,749-10,113,932)	737,904.7 (616,074.2-916,413.3)	8,219,803 (6,795,060-10,140,706)
**Staphylococcus aureus**	0.6	0.7	5 years	All age groups	Intra-abdominal infections	3,708,560 (3,230,649-4,247,259)	190,420,751 (150,105,337-247,875,672)	11,648,742 (10,046,572-13,633,216)	103,114,846 (89,605,149-118,138,370)	10,954,694 (9,096,416-13,761,679)	87,208,374 (72,274,953-105,411,839)
**Staphylococcus aureus**	0.6	0.7	5 years	6 weeks & elderly age group	LRI and thorax infections	5,430,727 (3,988,008-7,149,643)	239,975,552 (161,487,633-342,982,776)	17,463,312 (13,396,757-22,038,563)	257,609,097 (211,553,511-308,963,480)	16,238,107 (10,841,111-23,878,521)	98,133,492 (73,360,352-135,202,234)
**Staphylococcus aureus**	0.6	0.7	5 years	All age groups	LRI and thorax infections	35,822,134 (26,376,305-47,422,183)	2,160,638,076 (1,427,875,550-3,121,978,901)	148,622,431 (111,157,999-187,821,472)	1,878,673,323 (1,546,327,307-2,260,276,788)	165,367,043 (108,416,792-241,259,067)	770,232,847 (576,689,925-1,061,676,614)
**Staphylococcus aureus**	0.6	0.7	5 years	6 weeks & elderly age group	UTI	5,112,569 (4,188,692-6,401,307)	401,955,226 (280,729,322-555,071,632)	30,515,910 (24,143,854-38,349,983)	69,935,685 (57,029,353-85,550,880)	11,851,687 (8,341,509-17,020,099)	157,960,301 (119,193,423-205,772,327)
**Staphylococcus aureus**	0.6	0.7	5 years	All age groups	UTI	91,943,336 (75,382,600-114,519,213)	4,947,118,652 (3,456,382,828-6,801,777,206)	556,645,963 (441,400,697-698,505,457)	1,467,017,285 (1,155,271,868-1,885,802,745)	419,148,215 (297,115,475-597,749,251)	2,173,461,666 (1,681,036,852-2,671,198,535)
**Streptococcus pneumoniae - Improved**	both	0.9	5 years	6 weeks	BSI	3,269,375 (2,812,709-3,727,831)	11,861,603 (9,963,388-14,293,435)	4,225,265 (3,658,171-4,866,260)	4,172,009 (3,701,093-4,716,297)	951,665.8 (774,638.9-1,162,347)	5,831,329 (4,549,836-7,317,142)
**Streptococcus pneumoniae - Improved**	both	0.9	5 years	6 weeks & elderly age group	BSI	3,572,634 (3,059,014-4,084,805)	24,635,106 (20,266,504-31,160,845)	5,010,404 (4,322,962-5,836,086)	12,891,803 (11,567,133-14,361,923)	1,500,977 (1,237,014-1,832,169)	14,881,038 (12,155,009-18,279,396)
**Streptococcus pneumoniae**	both	0.9	5 years	6, 10, 14 weeks	BSI	2,630,422 (2,264,505-2,999,192)	9,584,372 (8,051,356-11,553,161)	3,398,370 (2,942,337-3,912,977)	3,320,900 (2,945,220-3,754,993)	765,555.4 (623,096.4-935,139.5)	4,666,847 (3,640,378-5,856,195)

**Streptococcus pneumoniae**	both	0.9	5 years	6, 10, 14 weeks & elderly age group	BSI	2,883,914 (2,468,320-3,297,953)	20,174,812 (16,588,227-25,529,913)	4,049,317 (3,493,724-4,717,901)	10,546,358 (9,457,337-11,751,283)	1,220,772 (1,006,141-1,490,432)	12,151,530 (9,930,126-14,926,267)
**Streptococcus pneumoniae**	current	current	5 years	6, 10, 14 weeks	BSI	2,125,029 (1,829,194-2,423,061)	8,817,145 (7,407,412-10,630,380)	2,937,548 (2,543,352-3,382,373)	3,325,614 (2,949,534-3,759,790)	186,318.4 (151,707.8-227,523.2)	3,639,312 (2,839,318-4,566,010)
**Streptococcus pneumoniae - Improved**	both	0.9	5 years	6 weeks	CNS infections	6,837,180 (5,961,802-7,910,909)	4,457,168 (3,783,178-5,312,229)	8,160,879 (6,737,681-9,993,567)	3,854,821 (3,436,678-4,367,363)	3,979,236 (3,072,571-5,266,414)	4,022,302 (3,406,116-4,864,752)
**Streptococcus pneumoniae - Improved**	both	0.9	5 years	6 weeks & elderly age group	CNS infections	7,028,808 (6,136,303-8,132,927)	6,111,055 (5,104,112-7,458,224)	8,704,797 (7,160,735-10,712,155)	4,502,667 (4,042,360-5,109,158)	4,249,848 (3,281,879-5,617,471)	4,881,524 (4,149,741-5,934,525)
**Streptococcus pneumoniae**	both	0.9	5 years	6, 10, 14 weeks	CNS infections	5,531,680 (4,823,451-6,399,923)	3,590,949 (3,047,955-4,279,830)	6,560,469 (5,415,700-8,037,701)	3,108,713 (2,771,480-3,522,413)	3,204,751 (2,474,543-4,241,406)	3,233,963 (2,737,336-3,911,969)
**Streptococcus pneumoniae**	both	0.9	5 years	6, 10, 14 weeks & elderly age group	CNS infections	5,690,649 (4,967,420-6,584,324)	4,957,277 (4,141,867-6,058,602)	7,016,182 (5,767,981-8,629,937)	3,646,141 (3,272,879-4,137,407)	3,428,900 (2,647,983-4,532,366)	3,945,640 (3,355,723-4,797,264)
**Streptococcus pneumoniae**	current	current	5 years	6, 10, 14 weeks	CNS infections	4,459,324 (3,888,412-5,159,246)	3,338,288 (2,833,470-3,978,595)	5,703,591 (4,708,795-6,985,930)	3,084,954 (2,750,315-3,495,569)	721,602.3 (557,377.1-955,244.9)	2,479,056 (2,098,469-3,000,943)
**Streptococcus pneumoniae - Improved**	both	0.9	5 years	6 weeks	Cardiac infections	359,949.5 (296,338.3-439,893.8)	2,192,086 (1,731,624-2,796,461)	1,358,623 (1,101,570-1,735,908)	828,728.2 (729,755.4-953,654.8)	105,050.9 (84,688.45-131,288.2)	900,885.5 (695,070.7-1,154,960)
**Streptococcus pneumoniae - Improved**	both	0.9	5 years	6 weeks & elderly age group	Cardiac infections	390,829.2 (322,412.8-476,986.6)	6,502,298 (4,980,586-8,463,240)	1,502,309 (1,215,515-1,925,489)	3,656,962 (3,232,672-4,213,164)	173,292.2 (143,509.5-212,852.5)	2,966,638 (2,422,572-3,695,920)
**Streptococcus pneumoniae**	both	0.9	5 years	6, 10, 14 weeks	Cardiac infections	291,910.7 (240,304.7-356,825.8)	1,780,778 (1,406,854-2,271,606)	1,102,160 (893,047.5-1,407,930)	671,447.7 (591,141.3-772,407.9)	84,674.56 (68,309.28-105,801.1)	731,670.3 (564,651-938,724.1)
**Streptococcus pneumoniae**	both	0.9	5 years	6, 10, 14 weeks & elderly age group	Cardiac infections	317,514.7 (261,884.7-387,578.4)	5,352,936 (4,100,130-6,969,409)	1,220,818 (987,608.8-1,565,158)	3,015,263 (2,664,898-3,474,091)	141,200.7 (116,938.5-173,300.2)	2,445,706 (1,994,550-3,046,601)
**Streptococcus pneumoniae**	current	current	5 years	6, 10, 14 weeks	Cardiac infections	236,191.4 (194,446.2-288,738.8)	1,656,859 (1,308,953-2,113,519)	956,451.7 (775,121.7-1,221,824)	667,283.2 (587,479-767,648.9)	17,615.9 (14,212.75-22,024.26)	555,546.7 (428,767.3-713,016.9)
**Streptococcus pneumoniae - Improved**	both	0.9	5 years	6 weeks	LRI and thorax infections	34,188,015 (27,341,644-41,998,766)	498,264,617 (381,043,257-642,308,770)	145,191,375 (113,080,374-182,127,635)	199,791,729 (170,940,232-232,813,961)	64,482,947 (47,286,488-86,806,415)	256,408,196 (192,033,899-339,407,488)
**Streptococcus pneumoniae - Improved**	both	0.9	5 years	6 weeks & elderly age group	LRI and thorax infections	38,804,266 (31,219,329-47,564,195)	713,907,300 (543,490,065-915,410,703)	167,034,351 (129,791,755-211,678,377)	374,611,094 (318,264,948-440,438,168)	90,256,082 (66,471,949-121,922,350)	429,534,800 (325,439,020-573,224,065)
**Streptococcus pneumoniae**	both	0.9	5 years	6, 10, 14 weeks	LRI and thorax infections	18,185,776 (14,541,014-22,328,870)	263,603,793 (201,664,568-339,958,104)	76,853,283 (59,944,828-96,488,595)	106,451,135 (90,977,178-123,935,536)	34,013,348 (24,957,067-45,759,348)	135,575,683 (101,556,257-179,485,794)

**Streptococcus pneumoniae**	both	0.9	5 years	6, 10, 14 weeks & elderly age group	LRI and thorax infections	20,676,184 (16,627,875-25,330,748)	379,985,875 (289,371,068-487,158,036)	88,702,693 (68,936,437-112,416,905)	200,842,354 (170,547,331-236,232,102)	47,917,463 (35,309,915-64,700,773)	229,071,968 (173,542,931-305,635,078)
**Streptococcus pneumoniae**	current	current	5 years	6, 10, 14 weeks	LRI and thorax infections	14,697,037 (11,751,762-18,045,157)	245,472,669 (187,793,056-316,574,733)	66,552,591 (51,922,294-83,565,346)	105,665,828 (90,304,352-123,016,170)	6,933,182 (5,095,612-9,334,068)	102,500,608 (76,779,887-135,692,762)

Table 16. Regional values for productivity loss from deaths in 2019

Vaccine scenarios are aligned with those presented in Kim C, Holm M, Frost I, Hasso-Agopsowicz M, Abbas K. Global and regional burden of attributable and associated bacterial antimicrobial resistance avertable by vaccination: modelling study. *BMJ Glob Health* 2023; **8**(7).

**Vaccine - Target disease**	**efficacy**	**coverage**	**duration**	**Target population**	**Infectious syndrome**	**AFRO**	**AMRO**	**EMRO**	**EURO**	**SEARO**	**WPRO**
**Acinetobacter baumannii - all**	0.7	0.7	5 years	6 weeks & elderly age group	Bacterial skin infections	14554407.3	20443099.9	84756413.7	132761174	65538448.2	96522984.1
**Acinetobacter baumannii - all**	0.7	0.7	5 years	All age groups	Bacterial skin infections	14554407.3	20443099.9	84756413.7	132761174	65538448.2	96522984.1
**Acinetobacter baumannii - BSI**	0.7	0.7	5 years	6 weeks & elderly age group	Bacterial skin infections	14554407.3	20443099.9	84756413.7	132761174	65538448.2	96522984.1
**Acinetobacter baumannii - BSI**	0.7	0.7	5 years	All age groups	Bacterial skin infections	14554407.3	20443099.9	84756413.7	132761174	65538448.2	96522984.1
**Acinetobacter baumannii - all**	0.7	0.7	5 years	6 weeks & elderly age group	BSI	526403417	1086955743	1324243670	3095585829	2587102349	6345291484
**Acinetobacter baumannii - all**	0.7	0.7	5 years	All age groups	BSI	526403417	1086955743	1324243670	3095585829	2587102349	6345291484
**Acinetobacter baumannii - BSI**	0.7	0.7	5 years	6 weeks & elderly age group	BSI	526403417	1086955743	1324243670	3095585829	2587102349	6345291484
**Acinetobacter baumannii - BSI**	0.7	0.7	5 years	All age groups	BSI	526403417	1086955743	1324243670	3095585829	2587102349	6345291484
**Acinetobacter baumannii - all**	0.7	0.7	5 years	6 weeks & elderly age group	Cardiac infections	8761456.42	45092012.3	93735958.5	180346630	108377420	264961888
**Acinetobacter baumannii - all**	0.7	0.7	5 years	All age groups	Cardiac infections	8761456.42	45092012.3	93735958.5	180346630	108377420	264961888
**Acinetobacter baumannii - BSI**	0.7	0.7	5 years	6 weeks & elderly age group	Cardiac infections	8761456.42	45092012.3	93735958.5	180346630	108377420	264961888

**Acinetobacter baumannii - BSI**	0.7	0.7	5 years	All age groups	Cardiac infections	8761456.42	45092012.3	93735958.5	180346630	108377420	264961888
**Acinetobacter baumannii - all**	0.7	0.7	5 years	6 weeks & elderly age group	LRI and thorax infections	543790819	672271424	608189679	1628993960	1426087603	809265471
**Acinetobacter baumannii - all**	0.7	0.7	5 years	All age groups	LRI and thorax infections	543790819	672271424	608189679	1628993960	1426087603	809265471
**Acinetobacter baumannii - BSI**	0.7	0.7	5 years	6 weeks & elderly age group	LRI and thorax infections	543790819	672271424	608189679	1628993960	1426087603	809265471
**Acinetobacter baumannii - BSI**	0.7	0.7	5 years	All age groups	LRI and thorax infections	543790819	672271424	608189679	1628993960	1426087603	809265471
**Acinetobacter baumannii - all**	0.7	0.7	5 years	6 weeks & elderly age group	Total	1102143654	1893400430	2337618836	5324558764	4313305375	7772664953
**Acinetobacter baumannii - all**	0.7	0.7	5 years	All age groups	Total	1102143654	1893400430	2337618836	5324558764	4313305375	7772664953
**Acinetobacter baumannii - BSI**	0.7	0.7	5 years	6 weeks & elderly age group	Total	1102143654	1893400430	2337618836	5324558764	4313305375	7772664953
**Acinetobacter baumannii - BSI**	0.7	0.7	5 years	All age groups	Total	1102143654	1893400430	2337618836	5324558764	4313305375	7772664953
**Acinetobacter baumannii - all**	0.7	0.7	5 years	6 weeks & elderly age group	UTI	8633554.94	68638151.2	226693114	286871172	126199555	256623127
**Acinetobacter baumannii - all**	0.7	0.7	5 years	All age groups	UTI	8633554.94	68638151.2	226693114	286871172	126199555	256623127
**Acinetobacter baumannii - BSI**	0.7	0.7	5 years	6 weeks & elderly age group	UTI	8633554.94	68638151.2	226693114	286871172	126199555	256623127
**Acinetobacter baumannii - BSI**	0.7	0.7	5 years	All age groups	UTI	8633554.94	68638151.2	226693114	286871172	126199555	256623127
**Enterococcus faecium**	0.7	0.7	5 years	6 weeks & elderly age group	Bone and joint infections	995631.683	1799367.97	1907130.56	3531372.6	1631905.01	2565701.13
**Enterococcus faecium**	0.7	0.7	5 years	All age groups	Bone and joint infections	995631.683	1799367.97	1907130.56	3531372.6	1631905.01	2565701.13
**Enterococcus faecium**	0.7	0.7	5 years	6 weeks & elderly age group	BSI	182828605	214715899	693797998	1008415144	243070016	619508637
**Enterococcus faecium**	0.7	0.7	5 years	All age groups	BSI	182828605	214715899	693797998	1008415144	243070016	619508637
**Enterococcus faecium**	0.7	0.7	5 years	6 weeks & elderly age group	Cardiac infections	2840282.9	6866830.95	54255803.4	69032582.1	9263307.26	25332960.7

**Enterococcus faecium**	0.7	0.7	5 years	All age groups	Cardiac infections	2840282.9	6866830.95	54255803.4	69032582.1	9263307.26	25332960.7
**Enterococcus faecium**	0.7	0.7	5 years	6 weeks & elderly age group	Intra-abdominal infections	148271768	197443623	1060849472	1351841549	511460004	728616376
**Enterococcus faecium**	0.7	0.7	5 years	All age groups	Intra-abdominal infections	148271768	197443623	1060849472	1351841549	511460004	728616376
**Enterococcus faecium**	0.7	0.7	5 years	6 weeks & elderly age group	Total	340102568	442340902	1893890984	2556834214	815164478	1457710350
**Enterococcus faecium**	0.7	0.7	5 years	All age groups	Total	340102568	442340902	1893890984	2556834214	815164478	1457710350
**Enterococcus faecium**	0.7	0.7	5 years	6 weeks & elderly age group	UTI	5166279.58	21515180.3	83080579.5	124013566	49739246	81686675.9
**Enterococcus faecium**	0.7	0.7	5 years	All age groups	UTI	5166279.58	21515180.3	83080579.5	124013566	49739246	81686675.9
**E. coli - non-diarrhogenic**	0.7	0.7	5 years	6 weeks & elderly age group	Bacterial skin infections	17072394	22114743	66175340.8	83447680.2	84929936.3	108220321
**E. coli - non-diarrhogenic**	0.7	0.7	5 years	All age groups	Bacterial skin infections	17072394	22114743	66175340.8	83447680.2	84929936.3	108220321
**ETEC**	0.6	0.7	5 years	6 months	Bacterial skin infections	17072394	22114743	66175340.8	83447680.2	84929936.3	108220321
**ExPEC - BSI**	0.7	0.7	5 years	6 weeks & elderly age group	Bacterial skin infections	17072394	22114743	66175340.8	83447680.2	84929936.3	108220321
**ExPEC - BSI**	0.7	0.7	5 years	All age groups	Bacterial skin infections	17072394	22114743	66175340.8	83447680.2	84929936.3	108220321
**ExPEC - UTI**	0.7	0.7	5 years	6 weeks & elderly age group	Bacterial skin infections	17072394	22114743	66175340.8	83447680.2	84929936.3	108220321
**ExPEC - UTI**	0.7	0.7	5 years	All age groups	Bacterial skin infections	17072394	22114743	66175340.8	83447680.2	84929936.3	108220321
**E. coli - non-diarrhogenic**	0.7	0.7	5 years	6 weeks & elderly age group	Bone and joint infections	4542282.28	10461392.8	8584116.13	10871524.8	12125466.5	16948237.9
**E. coli - non-diarrhogenic**	0.7	0.7	5 years	All age groups	Bone and joint infections	4542282.28	10461392.8	8584116.13	10871524.8	12125466.5	16948237.9
**ETEC**	0.6	0.7	5 years	6 months	Bone and joint infections	4542282.28	10461392.8	8584116.13	10871524.8	12125466.5	16948237.9
**ExPEC - BSI**	0.7	0.7	5 years	6 weeks & elderly age group	Bone and joint infections	4542282.28	10461392.8	8584116.13	10871524.8	12125466.5	16948237.9
**ExPEC - BSI**	0.7	0.7	5 years	All age groups	Bone and joint infections	4542282.28	10461392.8	8584116.13	10871524.8	12125466.5	16948237.9
**ExPEC - UTI**	0.7	0.7	5 years	6 weeks & elderly age group	Bone and joint infections	4542282.28	10461392.8	8584116.13	10871524.8	12125466.5	16948237.9

**ExPEC - UTI**	0.7	0.7	5 years	All age groups	Bone and joint infections	4542282.28	10461392.8	8584116.13	10871524.8	12125466.5	16948237.9
**E. coli - non-diarrhogenic**	0.7	0.7	5 years	6 weeks & elderly age group	BSI	411860464	589403788	1710474095	1010615257	714748921	969428672
**E. coli - non-diarrhogenic**	0.7	0.7	5 years	All age groups	BSI	411860464	589403788	1710474095	1010615257	714748921	969428672
**ETEC**	0.6	0.7	5 years	6 months	BSI	411860464	589403788	1710474095	1010615257	714748921	969428672
**ExPEC - BSI**	0.7	0.7	5 years	6 weeks & elderly age group	BSI	411860464	589403788	1710474095	1010615257	714748921	969428672
**ExPEC - BSI**	0.7	0.7	5 years	All age groups	BSI	411860464	589403788	1710474095	1010615257	714748921	969428672
**ExPEC - UTI**	0.7	0.7	5 years	6 weeks & elderly age group	BSI	411860464	589403788	1710474095	1010615257	714748921	969428672
**ExPEC - UTI**	0.7	0.7	5 years	All age groups	BSI	411860464	589403788	1710474095	1010615257	714748921	969428672
**E. coli - non-diarrhogenic**	0.7	0.7	5 years	6 weeks & elderly age group	Cardiac infections	6088967.39	14863324	136974105	50522802.7	25288700.3	38452041.7
**E. coli - non-diarrhogenic**	0.7	0.7	5 years	All age groups	Cardiac infections	6088967.39	14863324	136974105	50522802.7	25288700.3	38452041.7
**ETEC**	0.6	0.7	5 years	6 months	Cardiac infections	6088967.39	14863324	136974105	50522802.7	25288700.3	38452041.7
**ExPEC - BSI**	0.7	0.7	5 years	6 weeks & elderly age group	Cardiac infections	6088967.39	14863324	136974105	50522802.7	25288700.3	38452041.7
**ExPEC - BSI**	0.7	0.7	5 years	All age groups	Cardiac infections	6088967.39	14863324	136974105	50522802.7	25288700.3	38452041.7
**ExPEC - UTI**	0.7	0.7	5 years	6 weeks & elderly age group	Cardiac infections	6088967.39	14863324	136974105	50522802.7	25288700.3	38452041.7
**ExPEC - UTI**	0.7	0.7	5 years	All age groups	Cardiac infections	6088967.39	14863324	136974105	50522802.7	25288700.3	38452041.7
**E. coli - non-diarrhogenic**	0.7	0.7	5 years	6 weeks & elderly age group	CNS infections	410340711	276277305	60174519.1	116008862	307074923	124720131
**E. coli - non-diarrhogenic**	0.7	0.7	5 years	All age groups	CNS infections	410340711	276277305	60174519.1	116008862	307074923	124720131
**ETEC**	0.6	0.7	5 years	6 months	CNS infections	410340711	276277305	60174519.1	116008862	307074923	124720131
**ExPEC - BSI**	0.7	0.7	5 years	6 weeks & elderly age group	CNS infections	410340711	276277305	60174519.1	116008862	307074923	124720131
**ExPEC - BSI**	0.7	0.7	5 years	All age groups	CNS infections	410340711	276277305	60174519.1	116008862	307074923	124720131

**ExPEC - UTI**	0.7	0.7	5 years	6 weeks & elderly age group	CNS infections	410340711	276277305	60174519.1	116008862	307074923	124720131
**ExPEC - UTI**	0.7	0.7	5 years	All age groups	CNS infections	410340711	276277305	60174519.1	116008862	307074923	124720131
**E. coli - non-diarrhogenic**	0.7	0.7	5 years	6 weeks & elderly age group	Diarrhoea	525715235	381699910	22039950.8	36286069.1	770068103	20963744.2
**E. coli - non-diarrhogenic**	0.7	0.7	5 years	All age groups	Diarrhoea	525715235	381699910	22039950.8	36286069.1	770068103	20963744.2
**ETEC**	0.6	0.7	5 years	6 months	Diarrhoea	525715235	381699910	22039950.8	36286069.1	770068103	20963744.2
**ExPEC - BSI**	0.7	0.7	5 years	6 weeks & elderly age group	Diarrhoea	525715235	381699910	22039950.8	36286069.1	770068103	20963744.2
**ExPEC - BSI**	0.7	0.7	5 years	All age groups	Diarrhoea	525715235	381699910	22039950.8	36286069.1	770068103	20963744.2
**ExPEC - UTI**	0.7	0.7	5 years	6 weeks & elderly age group	Diarrhoea	525715235	381699910	22039950.8	36286069.1	770068103	20963744.2
**ExPEC - UTI**	0.7	0.7	5 years	All age groups	Diarrhoea	525715235	381699910	22039950.8	36286069.1	770068103	20963744.2
**E. coli - non-diarrhogenic**	0.7	0.7	5 years	6 weeks & elderly age group	Intra-abdominal infections	421215175	674357544	2064698350	2013831665	2266277047	2137022829
**E. coli - non-diarrhogenic**	0.7	0.7	5 years	All age groups	Intra-abdominal infections	421215175	674357544	2064698350	2013831665	2266277047	2137022829
**ETEC**	0.6	0.7	5 years	6 months	Intra-abdominal infections	421215175	674357544	2064698350	2013831665	2266277047	2137022829
**ExPEC - BSI**	0.7	0.7	5 years	6 weeks & elderly age group	Intra-abdominal infections	421215175	674357544	2064698350	2013831665	2266277047	2137022829
**ExPEC - BSI**	0.7	0.7	5 years	All age groups	Intra-abdominal infections	421215175	674357544	2064698350	2013831665	2266277047	2137022829
**ExPEC - UTI**	0.7	0.7	5 years	6 weeks & elderly age group	Intra-abdominal infections	421215175	674357544	2064698350	2013831665	2266277047	2137022829
**ExPEC - UTI**	0.7	0.7	5 years	All age groups	Intra-abdominal infections	421215175	674357544	2064698350	2013831665	2266277047	2137022829
**E. coli - non-diarrhogenic**	0.7	0.7	5 years	6 weeks & elderly age group	LRI and thorax infections	1595999454	1537501611	485678606	791489861	2443099102	762685254
**E. coli - non-diarrhogenic**	0.7	0.7	5 years	All age groups	LRI and thorax infections	1595999454	1537501611	485678606	791489861	2443099102	762685254
**ETEC**	0.6	0.7	5 years	6 months	LRI and thorax infections	1595999454	1537501611	485678606	791489861	2443099102	762685254
**ExPEC - BSI**	0.7	0.7	5 years	6 weeks & elderly age group	LRI and thorax infections	1595999454	1537501611	485678606	791489861	2443099102	762685254

**ExPEC - BSI**	0.7	0.7	5 years	All age groups	LRI and thorax infections	1595999454	1537501611	485678606	791489861	2443099102	762685254
**ExPEC - UTI**	0.7	0.7	5 years	6 weeks & elderly age group	LRI and thorax infections	1595999454	1537501611	485678606	791489861	2443099102	762685254
**ExPEC - UTI**	0.7	0.7	5 years	All age groups	LRI and thorax infections	1595999454	1537501611	485678606	791489861	2443099102	762685254
**E. coli - non-diarrhogenic**	0.7	0.7	5 years	6 weeks & elderly age group	Total	3450855760	3717143408	5035433593	4617037258	7413187520	4605599680
**E. coli - non-diarrhogenic**	0.7	0.7	5 years	All age groups	Total	3450855760	3717143408	5035433593	4617037258	7413187520	4605599680
**ETEC**	0.6	0.7	5 years	6 months	Total	3450855760	3717143408	5035433593	4617037258	7413187520	4605599680
**ExPEC - BSI**	0.7	0.7	5 years	6 weeks & elderly age group	Total	3450855760	3717143408	5035433593	4617037258	7413187520	4605599680
**ExPEC - BSI**	0.7	0.7	5 years	All age groups	Total	3450855760	3717143408	5035433593	4617037258	7413187520	4605599680
**ExPEC - UTI**	0.7	0.7	5 years	6 weeks & elderly age group	Total	3450855760	3717143408	5035433593	4617037258	7413187520	4605599680
**ExPEC - UTI**	0.7	0.7	5 years	All age groups	Total	3450855760	3717143408	5035433593	4617037258	7413187520	4605599680
**E. coli - non-diarrhogenic**	0.7	0.7	5 years	6 weeks & elderly age group	UTI	58021077.1	210463790	480634510	503963536	789575320	427158450
**E. coli - non-diarrhogenic**	0.7	0.7	5 years	All age groups	UTI	58021077.1	210463790	480634510	503963536	789575320	427158450
**ETEC**	0.6	0.7	5 years	6 months	UTI	58021077.1	210463790	480634510	503963536	789575320	427158450
**ExPEC - BSI**	0.7	0.7	5 years	6 weeks & elderly age group	UTI	58021077.1	210463790	480634510	503963536	789575320	427158450
**ExPEC - BSI**	0.7	0.7	5 years	All age groups	UTI	58021077.1	210463790	480634510	503963536	789575320	427158450
**ExPEC - UTI**	0.7	0.7	5 years	6 weeks & elderly age group	UTI	58021077.1	210463790	480634510	503963536	789575320	427158450
**ExPEC - UTI**	0.7	0.7	5 years	All age groups	UTI	58021077.1	210463790	480634510	503963536	789575320	427158450
**Group A streptococcus**	0.7	0.7	5 years	6 weeks	Bacterial skin infections	14388352.4	6387413.59	21576762.2	71879542.5	24700692.4	294728836
**Group A streptococcus**	0.7	0.7	5 years	6 weeks	Bone and joint infections	1449415.39	1121827.08	1216088.51	3796498.28	1317921.09	20257580.8
**Group A streptococcus**	0.7	0.7	5 years	6 weeks	BSI	57822885.4	27177666.4	58399360	112756671	31630119.6	433479026
**Group A streptococcus**	0.7	0.7	5 years	6 weeks	Cardiac infections	782634.561	623514.575	4583764.96	7085434.86	1505678.94	17830036.2

**Group A streptococcus**	0.7	0.7	5 years	6 weeks	Total	74443287.7	35310421.7	85775975.7	195518146	59154412.1	766295479
**Haemophilus influenzae type B**	0.93	0.9	5 years	6, 10, 14 weeks	CNS infections	17796180	10535474.1	1078034.39	4219054.59	4866113.23	5005339.12
**Haemophilus influenzae type B**	current	current	5 years	6, 10, 14 weeks	CNS infections	17796180	10535474.1	1078034.39	4219054.59	4866113.23	5005339.12
**Haemophilus influenzae type B**	0.69	0.9	5 years	6, 10, 14 weeks	LRI and thorax infections	87015484.9	50781870.3	23197493.7	28227869.6	38445523.5	40857085.2
**Haemophilus influenzae type B**	current	current	5 years	6, 10, 14 weeks	LRI and thorax infections	87015484.9	50781870.3	23197493.7	28227869.6	38445523.5	40857085.2
**Haemophilus influenzae type B**	0.69	0.9	5 years	6, 10, 14 weeks	Total	87015484.9	50781870.3	23197493.7	28227869.6	38445523.5	40857085.2
**Haemophilus influenzae type B**	0.93	0.9	5 years	6, 10, 14 weeks	Total	17796180	10535474.1	1078034.39	4219054.59	4866113.23	5005339.12
**Haemophilus influenzae type B**	current	current	5 years	6, 10, 14 weeks	Total	104811665	61317344.4	24275528.1	32446924.2	43311636.8	45862424.3
**Klebsiella pneumoniae - all**	0.7	0.7	5 years	6 weeks & elderly age group	Bacterial skin infections	9068135.71	9066316.42	36527457	34748301.4	34217563	31396672.8
**Klebsiella pneumoniae - all**	0.7	0.7	5 years	All age groups	Bacterial skin infections	9068135.71	9066316.42	36527457	34748301.4	34217563	31396672.8
**Klebsiella pneumoniae - BSI**	0.7	0.7	6 months	0 weeks (maternal)	Bacterial skin infections	9068135.71	9066316.42	36527457	34748301.4	34217563	31396672.8
**Klebsiella pneumoniae - all**	0.7	0.7	5 years	6 weeks & elderly age group	Bone and joint infections	4864721.75	6826849.22	8285013.51	8286420.38	9918415.71	8464267.37
**Klebsiella pneumoniae - all**	0.7	0.7	5 years	All age groups	Bone and joint infections	4864721.75	6826849.22	8285013.51	8286420.38	9918415.71	8464267.37
**Klebsiella pneumoniae - BSI**	0.7	0.7	6 months	0 weeks (maternal)	Bone and joint infections	4864721.75	6826849.22	8285013.51	8286420.38	9918415.71	8464267.37
**Klebsiella pneumoniae - all**	0.7	0.7	5 years	6 weeks & elderly age group	BSI	1863487972	1813545657	1802247397	2136943102	2188609243	1450036449
**Klebsiella pneumoniae - all**	0.7	0.7	5 years	All age groups	BSI	1863487972	1813545657	1802247397	2136943102	2188609243	1450036449

**Klebsiella pneumoniae - BSI**	0.7	0.7	6 months	0 weeks (maternal)	BSI	1863487972	1813545657	1802247397	2136943102	2188609243	1450036449
**Klebsiella pneumoniae - all**	0.7	0.7	5 years	6 weeks & elderly age group	Cardiac infections	19808491.7	32377074.5	127231937	89914096.8	56570675.5	54565660.8
**Klebsiella pneumoniae - all**	0.7	0.7	5 years	All age groups	Cardiac infections	19808491.7	32377074.5	127231937	89914096.8	56570675.5	54565660.8
**Klebsiella pneumoniae - BSI**	0.7	0.7	6 months	0 weeks (maternal)	Cardiac infections	19808491.7	32377074.5	127231937	89914096.8	56570675.5	54565660.8
**Klebsiella pneumoniae - all**	0.7	0.7	5 years	6 weeks & elderly age group	CNS infections	821167188	408863219	140307275	214002149	552709157	183442886
**Klebsiella pneumoniae - all**	0.7	0.7	5 years	All age groups	CNS infections	821167188	408863219	140307275	214002149	552709157	183442886
**Klebsiella pneumoniae - BSI**	0.7	0.7	6 months	0 weeks (maternal)	CNS infections	821167188	408863219	140307275	214002149	552709157	183442886
**Klebsiella pneumoniae - all**	0.7	0.7	5 years	6 weeks & elderly age group	Intra-abdominal infections	298428465	377191916	1421129223	1020315128	1204541467	784859409
**Klebsiella pneumoniae - all**	0.7	0.7	5 years	All age groups	Intra-abdominal infections	298428465	377191916	1421129223	1020315128	1204541467	784859409
**Klebsiella pneumoniae - BSI**	0.7	0.7	6 months	0 weeks (maternal)	Intra-abdominal infections	298428465	377191916	1421129223	1020315128	1204541467	784859409
**Klebsiella pneumoniae - all**	0.7	0.7	5 years	6 weeks & elderly age group	LRI and thorax infections	3055013587	2234724899	1255084348	1658848131	3382291720	1146844562
**Klebsiella pneumoniae - all**	0.7	0.7	5 years	All age groups	LRI and thorax infections	3055013587	2234724899	1255084348	1658848131	3382291720	1146844562
**Klebsiella pneumoniae - BSI**	0.7	0.7	6 months	0 weeks (maternal)	LRI and thorax infections	3055013587	2234724899	1255084348	1658848131	3382291720	1146844562
**Klebsiella pneumoniae - all**	0.7	0.7	5 years	6 weeks & elderly age group	Total	6088432472	4939043900	5002658644	5334484673	7615586667	3786714372
**Klebsiella pneumoniae - all**	0.7	0.7	5 years	All age groups	Total	6088432472	4939043900	5002658644	5334484673	7615586667	3786714372
**Klebsiella pneumoniae - BSI**	0.7	0.7	6 months	0 weeks (maternal)	Total	6088432472	4939043900	5002658644	5334484673	7615586667	3786714372

**Klebsiella pneumoniae - all**	0.7	0.7	5 years	6 weeks & elderly age group	UTI	16593911.2	56447969.5	211845993	171427344	186728426	127104465
**Klebsiella pneumoniae - all**	0.7	0.7	5 years	All age groups	UTI	16593911.2	56447969.5	211845993	171427344	186728426	127104465
**Klebsiella pneumoniae - BSI**	0.7	0.7	6 months	0 weeks (maternal)	UTI	16593911.2	56447969.5	211845993	171427344	186728426	127104465
**Mycobacterium tuberculosis - Improved**	0.8	0.7	10 years	0 weeks + boost every 10 years	TB	451310214	459082599	823513582	136309604	1392862639	305781070
**Mycobacterium tuberculosis - M72**	0.5	0.7	10 years	10 years + boost every 10 years	TB	451310214	459082599	823513582	136309604	1392862639	305781070
**Mycobacterium tuberculosis - Improved**	0.8	0.7	10 years	0 weeks + boost every 10 years	Total	451310214	459082599	823513582	136309604	1392862639	305781070
**Mycobacterium tuberculosis - M72**	0.5	0.7	10 years	10 years + boost every 10 years	Total	451310214	459082599	823513582	136309604	1392862639	305781070
**Non-typhoidal Salmonella**	0.8	0.7	5 years	6 weeks & 9 months	BSI	11132362.6	37395754.1	7470723.91	12250659.1	333375120	128272468
**Non-typhoidal Salmonella**	0.8	0.7	5 years	6 weeks & 9 months	Cardiac infections	103238.24	576727.517	591974.956	512218.004	9716606.13	5197231.13
**Non-typhoidal Salmonella**	0.8	0.7	5 years	6 weeks & 9 months	Diarrhoea	7377785.36	5333521.39	531963.77	2250046.57	77844675.9	19332328.4
**Non-typhoidal Salmonella**	0.8	0.7	5 years	6 weeks & 9 months	Total	34205649.1	62308111.6	9169773.81	16999239.2	500190506	186250468
**Non-typhoidal Salmonella**	0.8	0.7	5 years	6 weeks & 9 months	Typhoid, paratyphoid, and iNTS	15592262.9	19002108.6	575111.176	1986315.51	79254104.2	33448441.2
**Pseudomonas aeruginosa**	0.7	0.7	5 years	6 weeks & elderly age group	Bacterial skin infections	13945517.9	16642121.9	60756003.2	78851114.1	58311814.3	43492845.5
**Pseudomonas aeruginosa**	0.7	0.7	5 years	All age groups	Bacterial skin infections	13945517.9	16642121.9	60756003.2	78851114.1	58311814.3	43492845.5
**Pseudomonas aeruginosa**	0.7	0.7	5 years	6 weeks & elderly age group	Bone and joint infections	3109551.04	7220362.88	5684809.23	7018911.69	5223383.51	4959593.52
**Pseudomonas aeruginosa**	0.7	0.7	5 years	All age groups	Bone and joint infections	3109551.04	7220362.88	5684809.23	7018911.69	5223383.51	4959593.52
**Pseudomonas aeruginosa**	0.7	0.7	5 years	6 weeks & elderly age group	BSI	506667882	707622690	754147548	1359492289	1049877877	801921705

**Pseudomonas aeruginosa**	0.7	0.7	5 years	All age groups	BSI	506667882	707622690	754147548	1359492289	1049877877	801921705
**Pseudomonas aeruginosa**	0.7	0.7	5 years	6 weeks & elderly age group	Cardiac infections	6362096.17	16699069.1	54046051.6	68688791.7	31262254.5	31623510.3
**Pseudomonas aeruginosa**	0.7	0.7	5 years	All age groups	Cardiac infections	6362096.17	16699069.1	54046051.6	68688791.7	31262254.5	31623510.3
**Pseudomonas aeruginosa**	0.7	0.7	5 years	6 weeks & elderly age group	Intra-abdominal infections	104133268	190415441	1141515346	939316820	544015799	487331106
**Pseudomonas aeruginosa**	0.7	0.7	5 years	All age groups	Intra-abdominal infections	104133268	190415441	1141515346	939316820	544015799	487331106
**Pseudomonas aeruginosa**	0.7	0.7	5 years	6 weeks & elderly age group	LRI and thorax infections	995855575	1030112954	1137415206	1560788179	1648346563	596227955
**Pseudomonas aeruginosa**	0.7	0.7	5 years	All age groups	LRI and thorax infections	995855575	1030112954	1137415206	1560788179	1648346563	596227955
**Pseudomonas aeruginosa**	0.7	0.7	5 years	6 weeks & elderly age group	Total	1636041207	2000118811	3266358485	4128998762	3416177592	2030991482
**Pseudomonas aeruginosa**	0.7	0.7	5 years	All age groups	Total	1636041207	2000118811	3266358485	4128998762	3416177592	2030991482
**Pseudomonas aeruginosa**	0.7	0.7	5 years	6 weeks & elderly age group	UTI	5967316.49	31406171.6	112793521	114842657	79139900.5	65434767.4
**Pseudomonas aeruginosa**	0.7	0.7	5 years	All age groups	UTI	5967316.49	31406171.6	112793521	114842657	79139900.5	65434767.4
**Salmonella paratyphi**	0.7	0.7	5 years	9 months	Total	3318463.42	452595311	785836.067	652477.388	877291226	43710332.6
**Salmonella paratyphi**	0.7	0.7	5 years	9 months	Typhoid, paratyphoid, and iNTS	3318463.42	452595311	785836.067	652477.388	877291226	43710332.6
**Salmonella Typhi**	0.85	0.7	15 years	9 months	BSI	908207943	621335469	67701418.2	143871497	318292317	77623251.3
**Salmonella Typhi**	0.85	0.7	15 years	9 months	Cardiac infections	11197871.3	10535873.7	5111502.65	5867968.99	9429379.86	3463064.29
**Salmonella Typhi**	0.85	0.7	15 years	9 months	Total	1497433793	2300852742	82195320.1	157414422	3406784791	419424908
**Salmonella Typhi**	0.85	0.7	15 years	9 months	Typhoid, paratyphoid, and iNTS	578027978	1668981399	9382399.31	7674955.69	3079063095	338338592
**Shigella**	0.6	0.7	5 years	6 months	Diarrhoea	267077221	117026041	6656288.03	19765415.3	289902374	22815461.9
**Shigella**	0.6	0.7	5 years	6 months	Total	267077221	117026041	6656288.03	19765415.3	289902374	22815461.9
**Staphylococcus aureus**	0.6	0.7	5 years	6 weeks & elderly age group	Bacterial skin infections	97713889.2	98102114.3	110559816	318913458	314613795	260196963
**Staphylococcus aureus**	0.6	0.7	5 years	All age groups	Bacterial skin infections	97713889.2	98102114.3	110559816	318913458	314613795	260196963

**Staphylococcus aureus**	0.6	0.7	5 years	6 weeks & elderly age group	Bone and joint infections	12832858.5	31608213.2	32656161.4	69483104.2	37705845.3	84130199.2
**Staphylococcus aureus**	0.6	0.7	5 years	All age groups	Bone and joint infections	12832858.5	31608213.2	32656161.4	69483104.2	37705845.3	84130199.2
**Staphylococcus aureus**	0.6	0.7	5 years	6 weeks & elderly age group	BSI	414236458	854055367	1739851721	3248204795	921082532	3864305235
**Staphylococcus aureus**	0.6	0.7	5 years	All age groups	BSI	414236458	854055367	1739851721	3248204795	921082532	3864305235
**Staphylococcus aureus**	0.6	0.7	5 years	6 weeks & elderly age group	Cardiac infections	5420278.37	24557706.8	134193668	225999293	32339223.3	155443445
**Staphylococcus aureus**	0.6	0.7	5 years	All age groups	Cardiac infections	5420278.37	24557706.8	134193668	225999293	32339223.3	155443445
**Staphylococcus aureus**	0.6	0.7	5 years	6 weeks & elderly age group	CNS infections	266567847	232045906	44568011.2	119462237	213753523	99624838.8
**Staphylococcus aureus**	0.6	0.7	5 years	All age groups	CNS infections	266567847	232045906	44568011.2	119462237	213753523	99624838.8
**Staphylococcus aureus**	0.6	0.7	5 years	6 weeks & elderly age group	Intra-abdominal infections	294862026	551148725	1227069046	2092773953	1381484233	1694468948
**Staphylococcus aureus**	0.6	0.7	5 years	All age groups	Intra-abdominal infections	294862026	551148725	1227069046	2092773953	1381484233	1694468948
**Staphylococcus aureus**	0.6	0.7	5 years	6 weeks & elderly age group	LRI and thorax infections	2084004324	2557723285	1787610316	3716695454	2645098965	1786639916
**Staphylococcus aureus**	0.6	0.7	5 years	All age groups	LRI and thorax infections	2084004324	2557723285	1787610316	3716695454	2645098965	1786639916
**Staphylococcus aureus**	0.6	0.7	5 years	6 weeks & elderly age group	Total	3187963395	4409729516	5159296985	9949649730	5695745101	8057425043
**Staphylococcus aureus**	0.6	0.7	5 years	All age groups	Total	3187963395	4409729516	5159296985	9949649730	5695745101	8057425043
**Staphylococcus aureus**	0.6	0.7	5 years	6 weeks & elderly age group	UTI	12325713.1	60488197.9	82788245.8	158117436	149666983	112615498
**Staphylococcus aureus**	0.6	0.7	5 years	All age groups	UTI	12325713.1	60488197.9	82788245.8	158117436	149666983	112615498
**Streptococcus pneumoniae - Improved**	0.7	0.9	5 years	6 weeks	BSI	462136858	605455313	746251639	1370507207	568431471	1561810722
**Streptococcus pneumoniae - Improved**	0.7	0.9	5 years	6 weeks & elderly age group	BSI	462136858	605455313	746251639	1370507207	568431471	1561810722
**Streptococcus pneumoniae**	0.58	0.9	5 years	6, 10, 14 weeks	BSI	462136858	605455313	746251639	1370507207	568431471	1561810722

**Streptococcus pneumoniae**	0.58	0.9	5 years	6, 10, 14 weeks & elderly age group	BSI	462136858	605455313	746251639	1370507207	568431471	1561810722
**Streptococcus pneumoniae**	current	current	5 years	6, 10, 14 weeks	BSI	462136858	605455313	746251639	1370507207	568431471	1561810722
**Streptococcus pneumoniae - Improved**	0.7	0.9	5 years	6 weeks	Cardiac infections	7687711.58	18526588.9	53849787.8	74509697.7	22066908.6	63814679.8
**Streptococcus pneumoniae - Improved**	0.7	0.9	5 years	6 weeks & elderly age group	Cardiac infections	7687711.58	18526588.9	53849787.8	74509697.7	22066908.6	63814679.8
**Streptococcus pneumoniae**	0.58	0.9	5 years	6, 10, 14 weeks	Cardiac infections	7687711.58	18526588.9	53849787.8	74509697.7	22066908.6	63814679.8
**Streptococcus pneumoniae**	0.58	0.9	5 years	6, 10, 14 weeks & elderly age group	Cardiac infections	7687711.58	18526588.9	53849787.8	74509697.7	22066908.6	63814679.8
**Streptococcus pneumoniae**	current	current	5 years	6, 10, 14 weeks	Cardiac infections	7687711.58	18526588.9	53849787.8	74509697.7	22066908.6	63814679.8
**Streptococcus pneumoniae - Improved**	0.7	0.9	5 years	6 weeks	CNS infections	556728928	405862494	84162203.1	261743871	369490924	273703636
**Streptococcus pneumoniae - Improved**	0.7	0.9	5 years	6 weeks & elderly age group	CNS infections	556728928	405862494	84162203.1	261743871	369490924	273703636
**Streptococcus pneumoniae**	0.58	0.9	5 years	6, 10, 14 weeks	CNS infections	556728928	405862494	84162203.1	261743871	369490924	273703636
**Streptococcus pneumoniae**	0.58	0.9	5 years	6, 10, 14 weeks & elderly age group	CNS infections	556728928	405862494	84162203.1	261743871	369490924	273703636
**Streptococcus pneumoniae**	current	current	5 years	6, 10, 14 weeks	CNS infections	556728928	405862494	84162203.1	261743871	369490924	273703636
**Streptococcus pneumoniae - Improved**	0.5	0.9	5 years	6 weeks	LRI and thorax infections	4268497966	3975857569	2401726539	5016934057	4726523626	4937358224
**Streptococcus pneumoniae - Improved**	0.5	0.9	5 years	6 weeks & elderly age group	LRI and thorax infections	4268497966	3975857569	2401726539	5016934057	4726523626	4937358224
**Streptococcus pneumoniae**	0.27	0.9	5 years	6, 10, 14 weeks	LRI and thorax infections	4268497966	3975857569	2401726539	5016934057	4726523626	4937358224
**Streptococcus pneumoniae**	0.27	0.9	5 years	6, 10, 14 weeks & elderly age group	LRI and thorax infections	4268497966	3975857569	2401726539	5016934057	4726523626	4937358224
**Streptococcus pneumoniae**	current	current	5 years	6, 10, 14 weeks	LRI and thorax infections	4268497966	3975857569	2401726539	5016934057	4726523626	4937358224

**Streptococcus pneumoniae - Improved**	0.5	0.9	5 years	6 weeks	Total	4268497966	3975857569	2401726539	5016934057	4726523626	4937358224
**Streptococcus pneumoniae - Improved**	0.5	0.9	5 years	6 weeks & elderly age group	Total	4268497966	3975857569	2401726539	5016934057	4726523626	4937358224
**Streptococcus pneumoniae - Improved**	0.7	0.9	5 years	6 weeks	Total	1026553498	1029844396	884263629	1706760776	959989304	1899329037
**Streptococcus pneumoniae - Improved**	0.7	0.9	5 years	6 weeks & elderly age group	Total	1026553498	1029844396	884263629	1706760776	959989304	1899329037
**Streptococcus pneumoniae**	0.27	0.9	5 years	6, 10, 14 weeks	Total	4268497966	3975857569	2401726539	5016934057	4726523626	4937358224
**Streptococcus pneumoniae**	0.27	0.9	5 years	6, 10, 14 weeks & elderly age group	Total	4268497966	3975857569	2401726539	5016934057	4726523626	4937358224
**Streptococcus pneumoniae**	0.58	0.9	5 years	6, 10, 14 weeks	Total	1026553498	1029844396	884263629	1706760776	959989304	1899329037
**Streptococcus pneumoniae**	0.58	0.9	5 years	6, 10, 14 weeks & elderly age group	Total	1026553498	1029844396	884263629	1706760776	959989304	1899329037
**Streptococcus pneumoniae**	current	current	5 years	6, 10, 14 weeks	Total	5295051464	5005701965	3285990169	6723694832	5686512931	6836687261

Table 17. Regional values for averted productivity loss from deaths in 2019

Vaccine scenarios are aligned with those presented in Kim C, Holm M, Frost I, Hasso-Agopsowicz M, Abbas K. Global and regional burden of attributable and associated bacterial antimicrobial resistance avertable by vaccination: modelling study. *BMJ Glob Health* 2023; **8**(7).

Vaccine - Target disease	efficacy	coverage	duration	Target population	Infectious syndrome	AFRO	AMRO	EMRO	EURO	SEARO	WPRO
**Acinetobacter baumannii - all**	0.7	0.7	5 years	6 weeks & elderly age group	Bacterial skin infections	1020209.68	486950.061	340227.311	2076654.1	2417414.71	835206.757
**Acinetobacter baumannii - all**	0.7	0.7	5 years	All age groups	Bacterial skin infections	7131659.57	10017119	41530642.7	65052975.2	32113839.6	47296262.2
**Acinetobacter baumannii - BSI**	0.7	0.7	5 years	6 weeks & elderly age group	Bacterial skin infections	0	0	0	0	0	0
**Acinetobacter baumannii - BSI**	0.7	0.7	5 years	All age groups	Bacterial skin infections	0	0	0	0	0	0

**Acinetobacter baumannii - all**	0.7	0.7	5 years	6 weeks & elderly age group	BSI	32907614.1	49511968.6	29740962.8	120810934	64507895.7	132391834
**Acinetobacter baumannii - all**	0.7	0.7	5 years	All age groups	BSI	257937674	532608314	648879398	1516837056	1267680151	3109192827
**Acinetobacter baumannii - BSI**	0.7	0.7	5 years	6 weeks & elderly age group	BSI	32907614.1	49511968.6	29740962.8	120810934	64507895.7	132391834
**Acinetobacter baumannii - BSI**	0.7	0.7	5 years	All age groups	BSI	257937674	532608314	648879398	1516837056	1267680151	3109192827
**Acinetobacter baumannii - all**	0.7	0.7	5 years	6 weeks & elderly age group	Cardiac infections	456194.43	1727760.53	490505.186	2662800.73	1047054.73	4710692.08
**Acinetobacter baumannii - all**	0.7	0.7	5 years	All age groups	Cardiac infections	4293113.65	22095086	45930619.7	88369848.5	53104935.7	129831325
**Acinetobacter baumannii - BSI**	0.7	0.7	5 years	6 weeks & elderly age group	Cardiac infections	0	0	0	0	0	0
**Acinetobacter baumannii - BSI**	0.7	0.7	5 years	All age groups	Cardiac infections	0	0	0	0	0	0
**Acinetobacter baumannii - all**	0.7	0.7	5 years	6 weeks & elderly age group	LRI and thorax infections	70339844.9	58558835	18931192.5	57684620.4	80454242.7	25824271.5
**Acinetobacter baumannii - all**	0.7	0.7	5 years	All age groups	LRI and thorax infections	266457501	329412998	298012943	798207040	698782925	396540081
**Acinetobacter baumannii - BSI**	0.7	0.7	5 years	6 weeks & elderly age group	LRI and thorax infections	0	0	0	0	0	0
**Acinetobacter baumannii - BSI**	0.7	0.7	5 years	All age groups	LRI and thorax infections	0	0	0	0	0	0
**Acinetobacter baumannii - all**	0.7	0.7	5 years	6 weeks & elderly age group	Total	105121376	112358348	50919195.8	186940131	150025350	165371393
**Acinetobacter baumannii - all**	0.7	0.7	5 years	All age groups	Total	540050391	927766211	1145433229	2609033794	2113519634	3808605827
**Acinetobacter baumannii - BSI**	0.7	0.7	5 years	6 weeks & elderly age group	Total	32907614.1	49511968.6	29740962.8	120810934	64507895.7	132391834
**Acinetobacter baumannii - BSI**	0.7	0.7	5 years	All age groups	Total	257937674	532608314	648879398	1516837056	1267680151	3109192827
**Acinetobacter baumannii - all**	0.7	0.7	5 years	6 weeks & elderly age group	UTI	397512.733	2072833.82	1416307.98	3705122.32	1598742.04	1609388.19

**Acinetobacter baumannii - all**	0.7	0.7	5 years	All age groups	UTI	4230441.92	33632694.1	111079626	140566874	61837782.1	125745332
**Acinetobacter baumannii - BSI**	0.7	0.7	5 years	6 weeks & elderly age group	UTI	0	0	0	0	0	0
**Acinetobacter baumannii - BSI**	0.7	0.7	5 years	All age groups	UTI	0	0	0	0	0	0
**Enterococcus faecium**	0.7	0.7	5 years	6 weeks & elderly age group	Bone and joint infections	63959.2431	67642.94	5925.68714	26087.294	32196.2987	20662.942
**Enterococcus faecium**	0.7	0.7	5 years	All age groups	Bone and joint infections	487859.525	881690.305	934493.973	1730372.58	799633.457	1257193.55
**Enterococcus faecium**	0.7	0.7	5 years	6 weeks & elderly age group	BSI	10277894.6	8683307.45	8691060.07	30692078.9	5232536.68	11021582.9
**Enterococcus faecium**	0.7	0.7	5 years	All age groups	BSI	89586016.7	105210791	339961019	494123421	119104308	303559232
**Enterococcus faecium**	0.7	0.7	5 years	6 weeks & elderly age group	Cardiac infections	145558.746	205281.995	182550.846	709388.907	85686.9532	340056.515
**Enterococcus faecium**	0.7	0.7	5 years	All age groups	Cardiac infections	1391738.62	3364747.16	26585343.7	33825965.3	4539020.56	12413150.7
**Enterococcus faecium**	0.7	0.7	5 years	6 weeks & elderly age group	Intra-abdominal infections	9378754.77	5334672.26	5433585.67	21743874.5	7189604.38	10947451.5
**Enterococcus faecium**	0.7	0.7	5 years	All age groups	Intra-abdominal infections	72653166.4	96747375.4	519816241	662402359	250615402	357022024
**Enterococcus faecium**	0.7	0.7	5 years	6 weeks & elderly age group	Total	20084240.6	14823144.9	14645476.1	54509591.5	13106579.9	22758273.6
**Enterococcus faecium**	0.7	0.7	5 years	All age groups	Total	166650258	216747042	928006582	1252848765	399430594	714278072
**Enterococcus faecium**	0.7	0.7	5 years	6 weeks & elderly age group	UTI	218073.289	532240.281	332353.86	1338161.93	566555.562	428519.732
**Enterococcus faecium**	0.7	0.7	5 years	All age groups	UTI	2531476.99	10542438.4	40709483.9	60766647.4	24372230.5	40026471.2
**E. coli - non-diarrhogenic**	0.7	0.7	5 years	6 weeks & elderly age group	Bacterial skin infections	1412809.53	621555.985	296075.194	1433536.66	3549846.41	1091490.27
**E. coli - non-diarrhogenic**	0.7	0.7	5 years	All age groups	Bacterial skin infections	8365473.06	10836224.1	32425917	40889363.3	41615668.8	53027957.3
**ETEC**	0.6	0.7	5 years	6 months	Bacterial skin infections	0	0	0	0	0	0

**ExPEC - BSI**	0.7	0.7	5 years	6 weeks & elderly age group	Bacterial skin infections	0	0	0	0	0	0
**ExPEC - BSI**	0.7	0.7	5 years	All age groups	Bacterial skin infections	0	0	0	0	0	0
**ExPEC - UTI**	0.7	0.7	5 years	6 weeks & elderly age group	Bacterial skin infections	0	0	0	0	0	0
**ExPEC - UTI**	0.7	0.7	5 years	All age groups	Bacterial skin infections	0	0	0	0	0	0
**E. coli - non-diarrhogenic**	0.7	0.7	5 years	6 weeks & elderly age group	Bone and joint infections	518043.427	593683.185	55210.7893	177284.973	469395.948	236818.808
**E. coli - non-diarrhogenic**	0.7	0.7	5 years	All age groups	Bone and joint infections	2225718.32	5126082.48	4206216.91	5327047.15	5941478.6	8304636.55
**ETEC**	0.6	0.7	5 years	6 months	Bone and joint infections	0	0	0	0	0	0
**ExPEC - BSI**	0.7	0.7	5 years	6 weeks & elderly age group	Bone and joint infections	0	0	0	0	0	0
**ExPEC - BSI**	0.7	0.7	5 years	All age groups	Bone and joint infections	0	0	0	0	0	0
**ExPEC - UTI**	0.7	0.7	5 years	6 weeks & elderly age group	Bone and joint infections	0	0	0	0	0	0
**ExPEC - UTI**	0.7	0.7	5 years	All age groups	Bone and joint infections	0	0	0	0	0	0
**E. coli - non-diarrhogenic**	0.7	0.7	5 years	6 weeks & elderly age group	BSI	26384468.2	25827181.4	19324314.9	35066444.2	16881203.9	20142818.3
**E. coli - non-diarrhogenic**	0.7	0.7	5 years	All age groups	BSI	201811627	288807856	838132307	495201476	350226971	475020049
**ETEC**	0.6	0.7	5 years	6 months	BSI	0	0	0	0	0	0
**ExPEC - BSI**	0.7	0.7	5 years	6 weeks & elderly age group	BSI	26384468.2	25827181.4	19324314.9	35066444.2	16881203.9	20142818.3
**ExPEC - BSI**	0.7	0.7	5 years	All age groups	BSI	201811627	288807856	838132307	495201476	350226971	475020049
**ExPEC - UTI**	0.7	0.7	5 years	6 weeks & elderly age group	BSI	0	0	0	0	0	0
**ExPEC - UTI**	0.7	0.7	5 years	All age groups	BSI	0	0	0	0	0	0

**E. coli - non-diarrhogenic**	0.7	0.7	5 years	6 weeks & elderly age group	Cardiac infections	377278.109	497348.453	420124.71	768484.005	274023.537	587176.839
**E. coli - non-diarrhogenic**	0.7	0.7	5 years	All age groups	Cardiac infections	2983594.02	7283028.78	67117311.4	24756173.3	12391463.1	18841500.5
**ETEC**	0.6	0.7	5 years	6 months	Cardiac infections	0	0	0	0	0	0
**ExPEC - BSI**	0.7	0.7	5 years	6 weeks & elderly age group	Cardiac infections	0	0	0	0	0	0
**ExPEC - BSI**	0.7	0.7	5 years	All age groups	Cardiac infections	0	0	0	0	0	0
**ExPEC - UTI**	0.7	0.7	5 years	6 weeks & elderly age group	Cardiac infections	0	0	0	0	0	0
**ExPEC - UTI**	0.7	0.7	5 years	All age groups	Cardiac infections	0	0	0	0	0	0
**E. coli - non-diarrhogenic**	0.7	0.7	5 years	6 weeks & elderly age group	CNS infections	126329520	76357752.3	7423459.83	24601080.2	56588025.5	24525519.2
**E. coli - non-diarrhogenic**	0.7	0.7	5 years	All age groups	CNS infections	201066948	135375879	29485514.4	56844342.2	150466712	61112864
**ETEC**	0.6	0.7	5 years	6 months	CNS infections	0	0	0	0	0	0
**ExPEC - BSI**	0.7	0.7	5 years	6 weeks & elderly age group	CNS infections	0	0	0	0	0	0
**ExPEC - BSI**	0.7	0.7	5 years	All age groups	CNS infections	0	0	0	0	0	0
**ExPEC - UTI**	0.7	0.7	5 years	6 weeks & elderly age group	CNS infections	0	0	0	0	0	0
**ExPEC - UTI**	0.7	0.7	5 years	All age groups	CNS infections	0	0	0	0	0	0
**E. coli - non-diarrhogenic**	0.7	0.7	5 years	6 weeks & elderly age group	Diarrhoea	0	0	0	0	0	0
**E. coli - non-diarrhogenic**	0.7	0.7	5 years	All age groups	Diarrhoea	0	0	0	0	0	0
**ETEC**	0.6	0.7	5 years	6 months	Diarrhoea	62945558.4	43575911.2	2347938.21	3879984.86	61386018	2289599.1
**ExPEC - BSI**	0.7	0.7	5 years	6 weeks & elderly age group	Diarrhoea	0	0	0	0	0	0
**ExPEC - BSI**	0.7	0.7	5 years	All age groups	Diarrhoea	0	0	0	0	0	0
**ExPEC - UTI**	0.7	0.7	5 years	6 weeks & elderly age group	Diarrhoea	0	0	0	0	0	0

**ExPEC - UTI**	0.7	0.7	5 years	All age groups	Diarrhoea	0	0	0	0	0	0
**E. coli - non-diarrhogenic**	0.7	0.7	5 years	6 weeks & elderly age group	Intra-abdominal infections	28204107.9	17396663.2	11003112.6	40234850.8	32188720.2	34321374.5
**E. coli - non-diarrhogenic**	0.7	0.7	5 years	All age groups	Intra-abdominal infections	206395436	330435197	1011702191	986777516	1110475753	1047141186
**ETEC**	0.6	0.7	5 years	6 months	Intra-abdominal infections	0	0	0	0	0	0
**ExPEC - BSI**	0.7	0.7	5 years	6 weeks & elderly age group	Intra-abdominal infections	0	0	0	0	0	0
**ExPEC - BSI**	0.7	0.7	5 years	All age groups	Intra-abdominal infections	0	0	0	0	0	0
**ExPEC - UTI**	0.7	0.7	5 years	6 weeks & elderly age group	Intra-abdominal infections	0	0	0	0	0	0
**ExPEC - UTI**	0.7	0.7	5 years	All age groups	Intra-abdominal infections	0	0	0	0	0	0
**E. coli - non-diarrhogenic**	0.7	0.7	5 years	6 weeks & elderly age group	LRI and thorax infections	212319823	149996878	41678418	71695979.5	167005970	82028421.6
**E. coli - non-diarrhogenic**	0.7	0.7	5 years	All age groups	LRI and thorax infections	782039733	753375789	237982517	387830032	1197118560	373715774
**ETEC**	0.6	0.7	5 years	6 months	LRI and thorax infections	0	0	0	0	0	0
**ExPEC - BSI**	0.7	0.7	5 years	6 weeks & elderly age group	LRI and thorax infections	0	0	0	0	0	0
**ExPEC - BSI**	0.7	0.7	5 years	All age groups	LRI and thorax infections	0	0	0	0	0	0
**ExPEC - UTI**	0.7	0.7	5 years	6 weeks & elderly age group	LRI and thorax infections	0	0	0	0	0	0
**ExPEC - UTI**	0.7	0.7	5 years	All age groups	LRI and thorax infections	0	0	0	0	0	0
**E. coli - non-diarrhogenic**	0.7	0.7	5 years	6 weeks & elderly age group	Total	399909916	281946316	83369265.8	187231324	295177226	167828555
**E. coli - non-diarrhogenic**	0.7	0.7	5 years	All age groups	Total	1433318857	1634367314	2456562885	2244568082	3255128514	2246471609

**ETEC**	0.6	0.7	5 years	6 months	Total	62945558.4	43575911.2	2347938.21	3879984.86	61386018	2289599.1
**ExPEC - BSI**	0.7	0.7	5 years	6 weeks & elderly age group	Total	26384468.2	25827181.4	19324314.9	35066444.2	16881203.9	20142818.3
**ExPEC - BSI**	0.7	0.7	5 years	All age groups	Total	201811627	288807856	838132307	495201476	350226971	475020049
**ExPEC - UTI**	0.7	0.7	5 years	6 weeks & elderly age group	Total	4363864.77	10655253.7	3168549.83	13253663.4	18220041.4	4894935.5
**ExPEC - UTI**	0.7	0.7	5 years	All age groups	Total	28430327.8	103127257	235510910	246942132	386891907	209307641
**E. coli - non-diarrhogenic**	0.7	0.7	5 years	6 weeks & elderly age group	UTI	4363864.77	10655253.7	3168549.83	13253663.4	18220041.4	4894935.5
**E. coli - non-diarrhogenic**	0.7	0.7	5 years	All age groups	UTI	28430327.8	103127257	235510910	246942132	386891907	209307641
**ETEC**	0.6	0.7	5 years	6 months	UTI	0	0	0	0	0	0
**ExPEC - BSI**	0.7	0.7	5 years	6 weeks & elderly age group	UTI	0	0	0	0	0	0
**ExPEC - BSI**	0.7	0.7	5 years	All age groups	UTI	0	0	0	0	0	0
**ExPEC - UTI**	0.7	0.7	5 years	6 weeks & elderly age group	UTI	4363864.77	10655253.7	3168549.83	13253663.4	18220041.4	4894935.5
**ExPEC - UTI**	0.7	0.7	5 years	All age groups	UTI	28430327.8	103127257	235510910	246942132	386891907	209307641
**Group A streptococcus**	0.7	0.7	5 years	6 weeks	Bacterial skin infections	1099296.45	237397.998	88244.0759	1092353.66	685277.406	1983587.64
**Group A streptococcus**	0.7	0.7	5 years	6 weeks	Bone and joint infections	72991.4757	40971.697	3204.37546	22818.5702	19568.954	89720.9682
**Group A streptococcus**	0.7	0.7	5 years	6 weeks	BSI	8343790.62	3212060.66	2729351.9	12643409.6	2256435.2	29360279.5
**Group A streptococcus**	0.7	0.7	5 years	6 weeks	Cardiac infections	104067.809	54826.9683	61034.6022	286874.205	42954.3747	1105151.34
**Group A streptococcus**	0.7	0.7	5 years	6 weeks	Total	9620146.35	3545257.32	2881834.95	14045456	3004235.94	32538739.5
**Haemophilus influenzae type B**	0.93	0.9	5 years	6, 10, 14 weeks	CNS infections	9591863.04	4885170.15	268250.618	1998200.81	1651813.12	1978255.77
**Haemophilus influenzae type B**	current	current	5 years	6, 10, 14 weeks	CNS infections	7793990.58	4614190.97	277873.428	1904450.79	1660230.96	1874009.44
**Haemophilus influenzae type B**	0.69	0.9	5 years	6, 10, 14 weeks	LRI and thorax infections	30785191.2	15739415.6	5515333.36	6646137.33	8856295.6	11177069.1

**Haemophilus influenzae type B**	current	current	5 years	6, 10, 14 weeks	LRI and thorax infections	25070450.9	14828551.7	5766573.23	6249201.82	8925963.32	10634057.3
**Haemophilus influenzae type B**	0.69	0.9	5 years	6, 10, 14 weeks	Total	30785191.2	15739415.6	5515333.36	6646137.33	8856295.6	11177069.1
**Haemophilus influenzae type B**	0.93	0.9	5 years	6, 10, 14 weeks	Total	9591863.04	4885170.15	268250.618	1998200.81	1651813.12	1978255.77
**Haemophilus influenzae type B**	current	current	5 years	6, 10, 14 weeks	Total	32864441.4	19442742.7	6044446.66	8153652.62	10586194.3	12508066.7
**Klebsiella pneumoniae - all**	0.7	0.7	5 years	6 weeks & elderly age group	Bacterial skin infections	698689.272	237706.512	141171.271	671909.854	1315768.99	317859.797
**Klebsiella pneumoniae - all**	0.7	0.7	5 years	All age groups	Bacterial skin infections	4443386.5	4442495.04	17898454	17026667.7	16766605.9	15384369.7
**Klebsiella pneumoniae - BSI**	0.7	0.7	6 months	0 weeks (maternal)	Bacterial skin infections	0	0	0	0	0	0
**Klebsiella pneumoniae - all**	0.7	0.7	5 years	6 weeks & elderly age group	Bone and joint infections	230610.126	192967.079	19277.9739	61414.1998	146435.508	54412.4629
**Klebsiella pneumoniae - all**	0.7	0.7	5 years	All age groups	Bone and joint infections	2383713.66	3345156.12	4059656.62	4060345.99	4860023.7	4147491.01
**Klebsiella pneumoniae - BSI**	0.7	0.7	6 months	0 weeks (maternal)	Bone and joint infections	0	0	0	0	0	0
**Klebsiella pneumoniae - all**	0.7	0.7	5 years	6 weeks & elderly age group	BSI	177662226	125576521	60458082.3	173880770	91305412	75402618.8
**Klebsiella pneumoniae - all**	0.7	0.7	5 years	All age groups	BSI	913109106	888637372	883101225	1047102120	1072418529	710517860
**Klebsiella pneumoniae - BSI**	0.7	0.7	6 months	0 weeks (maternal)	BSI	607317701	576773762	133384096	413487599	600207734	177081197
**Klebsiella pneumoniae - all**	0.7	0.7	5 years	6 weeks & elderly age group	Cardiac infections	2465749.32	2465608	1146064.16	3678438.45	1438934.91	1967271.71
**Klebsiella pneumoniae - all**	0.7	0.7	5 years	All age groups	Cardiac infections	9706160.93	15864766.5	62343649.1	44057907.4	27719631	26737173.8
**Klebsiella pneumoniae - BSI**	0.7	0.7	6 months	0 weeks (maternal)	Cardiac infections	0	0	0	0	0	0

**Klebsiella pneumoniae - all**	0.7	0.7	5 years	6 weeks & elderly age group	CNS infections	224120275	96630408.3	12620607	39187504	81566892	29613987
**Klebsiella pneumoniae - all**	0.7	0.7	5 years	All age groups	CNS infections	402371922	200342977	68750564.6	104861053	270827487	89887014.3
**Klebsiella pneumoniae - BSI**	0.7	0.7	6 months	0 weeks (maternal)	CNS infections	0	0	0	0	0	0
**Klebsiella pneumoniae - all**	0.7	0.7	5 years	6 weeks & elderly age group	Intra-abdominal infections	17628429.1	8096330.59	5950332.28	18479890.2	14577148.9	10825876.4
**Klebsiella pneumoniae - all**	0.7	0.7	5 years	All age groups	Intra-abdominal infections	146229948	184824039	696353320	499954413	590225319	384581110
**Klebsiella pneumoniae - BSI**	0.7	0.7	6 months	0 weeks (maternal)	Intra-abdominal infections	0	0	0	0	0	0
**Klebsiella pneumoniae - all**	0.7	0.7	5 years	6 weeks & elderly age group	LRI and thorax infections	611861065	352931245	112610151	193926592	386422021	170395434
**Klebsiella pneumoniae - all**	0.7	0.7	5 years	All age groups	LRI and thorax infections	1496956658	1095015201	614991330	812835584	1657322943	561953835
**Klebsiella pneumoniae - BSI**	0.7	0.7	6 months	0 weeks (maternal)	LRI and thorax infections	0	0	0	0	0	0
**Klebsiella pneumoniae - all**	0.7	0.7	5 years	6 weeks & elderly age group	Total	1035753772	588547962	194058874	433878133	580457616	289760994
**Klebsiella pneumoniae - all**	0.7	0.7	5 years	All age groups	Total	2983331911	2420131511	2451302736	2613897490	3731637467	1855490042
**Klebsiella pneumoniae - BSI**	0.7	0.7	6 months	0 weeks (maternal)	Total	607317701	576773762	133384096	413487599	600207734	177081197
**Klebsiella pneumoniae - all**	0.7	0.7	5 years	6 weeks & elderly age group	UTI	1086728.03	2417174.84	1113187.75	3991613.88	3685002.27	1183534.33
**Klebsiella pneumoniae - all**	0.7	0.7	5 years	All age groups	UTI	8131016.5	27659505.1	103804537	83999398.3	91496928.8	62281187.8
**Klebsiella pneumoniae - BSI**	0.7	0.7	6 months	0 weeks (maternal)	UTI	0	0	0	0	0	0
**Mycobacterium tuberculosis - Improved**	0.8	0.7	10 years	0 weeks + boost every 10 years	TB	252733720	257086255	461167606	76333378	780003078	171237399

**Mycobacterium tuberculosis - M72**	0.5	0.7	10 years	10 years + boost every 10 years	TB	132847632	135894724	280395016	45140054.2	469584103	101373315
**Mycobacterium tuberculosis - Improved**	0.8	0.7	10 years	0 weeks + boost every 10 years	Total	252733720	257086255	461167606	76333378	780003078	171237399
**Mycobacterium tuberculosis - M72**	0.5	0.7	10 years	10 years + boost every 10 years	Total	132847632	135894724	280395016	45140054.2	469584103	101373315
**Non-typhoidal Salmonella**	0.8	0.7	5 years	6 weeks & 9 months	BSI	1229045.45	3506724.15	312218.041	1692460.01	20567213.1	8420928.38
**Non-typhoidal Salmonella**	0.8	0.7	5 years	6 weeks & 9 months	Cardiac infections	10500.0808	47392.3834	7171.00632	32766.5365	314166.183	300485.071
**Non-typhoidal Salmonella**	0.8	0.7	5 years	6 weeks & 9 months	Diarrhoea	2859495.11	2353310.11	118845.097	943351.019	22942363.7	7237789.99
**Non-typhoidal Salmonella**	0.8	0.7	5 years	6 weeks & 9 months	Total	9401752.4	8769690.48	502866.43	2989372.37	52634184.5	19645162.7
**Non-typhoidal Salmonella**	0.8	0.7	5 years	6 weeks & 9 months	Typhoid, paratyphoid, and iNTS	5302711.76	2862263.83	64632.2857	320794.804	8810441.45	3685959.3
**Pseudomonas aeruginosa**	0.7	0.7	5 years	6 weeks & elderly age group	Bacterial skin infections	0	0	0	0	0	0
**Pseudomonas aeruginosa**	0.7	0.7	5 years	All age groups	Bacterial skin infections	0	0	0	0	0	0
**Pseudomonas aeruginosa**	0.7	0.7	5 years	6 weeks & elderly age group	Bone and joint infections	0	0	0	0	0	0
**Pseudomonas aeruginosa**	0.7	0.7	5 years	All age groups	Bone and joint infections	0	0	0	0	0	0
**Pseudomonas aeruginosa**	0.7	0.7	5 years	6 weeks & elderly age group	BSI	29217548.5	28406060.1	13134327.9	52367752.7	23333537.3	18088120.9
**Pseudomonas aeruginosa**	0.7	0.7	5 years	All age groups	BSI	248267262	346735118	369532299	666151222	514440160	392941635
**Pseudomonas aeruginosa**	0.7	0.7	5 years	6 weeks & elderly age group	Cardiac infections	0	0	0	0	0	0
**Pseudomonas aeruginosa**	0.7	0.7	5 years	All age groups	Cardiac infections	0	0	0	0	0	0
**Pseudomonas aeruginosa**	0.7	0.7	5 years	6 weeks & elderly age group	Intra-abdominal infections	0	0	0	0	0	0
**Pseudomonas aeruginosa**	0.7	0.7	5 years	All age groups	Intra-abdominal infections	0	0	0	0	0	0

**Pseudomonas aeruginosa**	0.7	0.7	5 years	6 weeks & elderly age group	LRI and thorax infections	181539303	136946936	78704552.2	136794183	166915090	63611974.7
**Pseudomonas aeruginosa**	0.7	0.7	5 years	All age groups	LRI and thorax infections	487969232	504755348	557333451	764786208	807689816	292151698
**Pseudomonas aeruginosa**	0.7	0.7	5 years	6 weeks & elderly age group	Total	210756851	165352996	91838880.1	189161935	190248628	81700095.6
**Pseudomonas aeruginosa**	0.7	0.7	5 years	All age groups	Total	736236494	851490466	926865749	1430937429	1322129976	685093333
**Pseudomonas aeruginosa**	0.7	0.7	5 years	6 weeks & elderly age group	UTI	0	0	0	0	0	0
**Pseudomonas aeruginosa**	0.7	0.7	5 years	All age groups	UTI	0	0	0	0	0	0
**Salmonella paratyphi**	0.7	0.7	5 years	9 months	Total	468489.688	29853052.9	32256.4941	58177.4198	52609712.8	3664102.79
**Salmonella paratyphi**	0.7	0.7	5 years	9 months	Typhoid, paratyphoid, and iNTS	468489.688	29853052.9	32256.4941	58177.4198	52609712.8	3664102.79
**Salmonella Typhi**	0.85	0.7	15 years	9 months	BSI	91219577.9	49525382.8	3096543.8	11160878.6	19185014.3	5786727.05
**Salmonella Typhi**	0.85	0.7	15 years	9 months	Cardiac infections	1805793.79	1530758.4	94480.5062	496160.824	609733.518	210936.32
**Salmonella Typhi**	0.85	0.7	15 years	9 months	Total	335171141	691393089	5818652.8	12889906.5	1148585112	107132520
**Salmonella Typhi**	0.85	0.7	15 years	9 months	Typhoid, paratyphoid, and iNTS	242145769	640336947	2627628.49	1232867.09	1128790364	101134857
**Shigella**	0.6	0.7	5 years	6 months	Diarrhoea	74327415.4	29669522.8	1634146.82	4779186.31	42331762	4862984.46
**Shigella**	0.6	0.7	5 years	6 months	Total	74327415.4	29669522.8	1634146.82	4779186.31	42331762	4862984.46
**Staphylococcus aureus**	0.6	0.7	5 years	6 weeks & elderly age group	Bacterial skin infections	12688628.5	5764986.36	1246384.42	12122712.5	25714208.6	5746871.46
**Staphylococcus aureus**	0.6	0.7	5 years	All age groups	Bacterial skin infections	41039833.5	41202888	46435122.5	133943652	132137794	109282724
**Staphylococcus aureus**	0.6	0.7	5 years	6 weeks & elderly age group	Bone and joint infections	564245.192	751003.379	72411.0212	309700.42	488830.656	342308.461
**Staphylococcus aureus**	0.6	0.7	5 years	All age groups	Bone and joint infections	5389800.55	13275449.6	13715587.8	29182903.8	15836455	35334683.7
**Staphylococcus aureus**	0.6	0.7	5 years	6 weeks & elderly age group	BSI	19073242.4	29321658	26363480.4	94226043.4	18314948.3	67048653.9

**Staphylococcus aureus**	0.6	0.7	5 years	All age groups	BSI	173979312	358703254	730737723	1364246014	386854664	1623008199
**Staphylococcus aureus**	0.6	0.7	5 years	6 weeks & elderly age group	Cardiac infections	272132.981	779235.011	552849.268	2260648.82	297969.898	2401666.19
**Staphylococcus aureus**	0.6	0.7	5 years	All age groups	Cardiac infections	2276516.92	10314236.9	56361340.5	94919703	13582473.8	65286246.9
**Staphylococcus aureus**	0.6	0.7	5 years	6 weeks & elderly age group	CNS infections	49138862	36980678.7	2517206.64	11829825.4	18653446	9335381.43
**Staphylococcus aureus**	0.6	0.7	5 years	All age groups	CNS infections	111958496	97459280.7	18718564.7	50174139.7	89776479.7	41842432.3
**Staphylococcus aureus**	0.6	0.7	5 years	6 weeks & elderly age group	Intra-abdominal infections	22961904.8	16889840.9	9728042.18	47982201.8	23573033.3	32958662.3
**Staphylococcus aureus**	0.6	0.7	5 years	All age groups	Intra-abdominal infections	123842051	231482465	515368999	878965060	580223378	711676958
**Staphylococcus aureus**	0.6	0.7	5 years	6 weeks & elderly age group	LRI and thorax infections	373089074	362376205	138740337	271648762	287256616	180501929
**Staphylococcus aureus**	0.6	0.7	5 years	All age groups	LRI and thorax infections	875281816	1074243780	750796333	1561012091	1110941565	750388765
**Staphylococcus aureus**	0.6	0.7	5 years	6 weeks & elderly age group	Total	478591343	455592920	179840942	443906016	377423062	299374150
**Staphylococcus aureus**	0.6	0.7	5 years	All age groups	Total	1338944626	1852086397	2166904734	4178852887	2392212942	3384118518
**Staphylococcus aureus**	0.6	0.7	5 years	6 weeks & elderly age group	UTI	803252.954	2729312.29	620231.288	3526121.97	3124008.89	1038676.47
**Staphylococcus aureus**	0.6	0.7	5 years	All age groups	UTI	5176799.51	25405043.1	34771063.2	66409323.1	62860133	47298509.1
**Streptococcus pneumoniae - Improved**	0.7	0.9	5 years	6 weeks	BSI	88965547.8	91876067.1	46957418.6	174885803	48768089	104180061
**Streptococcus pneumoniae - Improved**	0.7	0.9	5 years	6 weeks & elderly age group	BSI	88965547.8	91876067.1	46957418.6	174885803	48768089	104180061
**Streptococcus pneumoniae**	0.58	0.9	5 years	6, 10, 14 weeks	BSI	71984426.8	74039662.2	37353616.8	141120946	39239283.8	83391053.3
**Streptococcus pneumoniae**	0.58	0.9	5 years	6, 10, 14 weeks & elderly age group	BSI	71984426.8	74039662.2	37353616.8	141120946	39239283.8	83391053.3
**Streptococcus pneumoniae**	current	current	5 years	6, 10, 14 weeks	BSI	58132822.7	64000037.2	37413963.6	129679971	9483918.38	64990564.5

**Streptococcus pneumoniae - Improved**	0.7	0.9	5 years	6 weeks	Cardiac infections	1179637.51	2013060.17	907620.329	3788300.07	771003.529	3602544.14
**Streptococcus pneumoniae - Improved**	0.7	0.9	5 years	6 weeks & elderly age group	Cardiac infections	1179637.51	2013060.17	907620.329	3788300.07	771003.529	3602544.14
**Streptococcus pneumoniae**	0.58	0.9	5 years	6, 10, 14 weeks	Cardiac infections	946831.591	1608251.68	729250.349	3045707.85	611558.453	2897211.06
**Streptococcus pneumoniae**	0.58	0.9	5 years	6, 10, 14 weeks & elderly age group	Cardiac infections	946831.591	1608251.68	729250.349	3045707.85	611558.453	2897211.06
**Streptococcus pneumoniae**	current	current	5 years	6, 10, 14 weeks	Cardiac infections	769280.736	1402559.62	727018.464	2829946.52	145826.188	2243350.49
**Streptococcus pneumoniae - Improved**	0.7	0.9	5 years	6 weeks	CNS infections	168383042	106625293	9332781.46	51254630.6	58261726.5	42736912.6
**Streptococcus pneumoniae - Improved**	0.7	0.9	5 years	6 weeks & elderly age group	CNS infections	168383042	106625293	9332781.46	51254630.6	58261726.5	42736912.6
**Streptococcus pneumoniae**	0.58	0.9	5 years	6, 10, 14 weeks	CNS infections	136784162	85824558.3	7518433.67	41275293.1	46681807.5	34267398.7
**Streptococcus pneumoniae**	0.58	0.9	5 years	6, 10, 14 weeks & elderly age group	CNS infections	136784162	85824558.3	7518433.67	41275293.1	46681807.5	34267398.7
**Streptococcus pneumoniae**	current	current	5 years	6, 10, 14 weeks	CNS infections	110276668	74618035.2	7463841.36	38358886.5	10863425.5	26554350.6
**Streptococcus pneumoniae - Improved**	0.5	0.9	5 years	6 weeks	LRI and thorax infections	1189980417	991075327	330423713	710904908	952661567	861632459
**Streptococcus pneumoniae - Improved**	0.5	0.9	5 years	6 weeks & elderly age group	LRI and thorax infections	1189980417	991075327	330423713	710904908	952661567	861632459
**Streptococcus pneumoniae**	0.27	0.9	5 years	6, 10, 14 weeks	LRI and thorax infections	641816681	534095531	177964084	383058580	513354693	464461579
**Streptococcus pneumoniae**	0.27	0.9	5 years	6, 10, 14 weeks & elderly age group	LRI and thorax infections	641816681	534095531	177964084	383058580	513354693	464461579
**Streptococcus pneumoniae**	current	current	5 years	6, 10, 14 weeks	LRI and thorax infections	520021175	464404707	177191422	356454630	124568820	362751662
**Streptococcus pneumoniae - Improved**	0.5	0.9	5 years	6 weeks	Total	1189980417	991075327	330423713	710904908	952661567	861632459

**Streptococcus pneumoniae - Improved**	0.5	0.9	5 years	6 weeks & elderly age group	Total	1189980417	991075327	330423713	710904908	952661567	861632459
**Streptococcus pneumoniae - Improved**	0.7	0.9	5 years	6 weeks	Total	258528227	200514421	57197820.4	229928734	107800819	150519518
**Streptococcus pneumoniae - Improved**	0.7	0.9	5 years	6 weeks & elderly age group	Total	258528227	200514421	57197820.4	229928734	107800819	150519518
**Streptococcus pneumoniae**	0.27	0.9	5 years	6, 10, 14 weeks	Total	641816681	534095531	177964084	383058580	513354693	464461579
**Streptococcus pneumoniae**	0.27	0.9	5 years	6, 10, 14 weeks & elderly age group	Total	641816681	534095531	177964084	383058580	513354693	464461579
**Streptococcus pneumoniae**	0.58	0.9	5 years	6, 10, 14 weeks	Total	209715420	161472472	45601300.9	185441947	86532649.7	120555663
**Streptococcus pneumoniae**	0.58	0.9	5 years	6, 10, 14 weeks & elderly age group	Total	209715420	161472472	45601300.9	185441947	86532649.7	120555663
**Streptococcus pneumoniae**	current	current	5 years	6, 10, 14 weeks	Total	689199947	604425339	222796245	527323434	145061990	456539927

S8. References

1. Wilkinson I, Wilkinson IB, Raine T, et al. Oxford handbook of clinical medicine: Oxford university press; 2017.
2. Knight GM. Drug Groups. 2019. https://github.com/gwenknight/empiricprescribing/blob/master/data/drug_groups.csv (accessed December 2019).
3. wikipedia. List of Antibiotics. 2019. https://en.wikipedia.org/wiki/List_of_antibiotics (accessed December 2019).
4. Rawat D, Nair D. Extended-spectrum β-lactamases in Gram Negative Bacteria. *J Glob Infect Dis* 2010; **2**(3): 263-74.
5. Somboro AM, Sekyere JO, Amoako DG, Essack SY, Bester LA. Diversity and Proliferation of Metallo-β-Lactamases: a Clarion Call for Clinically Effective Metallo-β-Lactamase Inhibitors. *Applied and Environmental Microbiology* 2018; **84**(18): e00698-18.
6. Queenan AM, Bush K. Carbapenemases: the versatile beta-lactamases. *Clin Microbiol Rev* 2007; **20**(3): 440-58, table of contents.
7. The World Bank Group. World Bank Country and Lending Groups. 2020.
8. The World Health Organisation. Cost effectiveness and strategic planning (WHO-CHOICE): Alphabetical List of WHO Member States 2020.
9. Shuster JJ. Cochrane handbook for systematic reviews for interventions, Version 5.1. 0, published 3/2011. Julian PT Higgins and Sally Green, Editors. Wiley Online Library; 2011.
10. Wan X, Wang W, Liu J, Tong T. Estimating the sample mean and standard deviation from the sample size, median, range and/or interquartile range. *BMC Medical Research Methodology* 2014; **14**(1): 135.
11. Piburn J. wbstats: Oak Ridge National Lab.(ORNL), Oak Ridge, TN (United States), 2016.
12. IBAN. Country currency codes. 2023. www.iban.com/currency-codes2023.

13. Naylor NR, Atun R, Zhu N, et al. Estimating the burden of antimicrobial resistance: a systematic literature review. *Antimicrob Resist Infect Control* 2018; **7**: 58.
14. Founou RC, Founou LL, Essack SY. Clinical and economic impact of antibiotic resistance in developing countries: A systematic review and meta-analysis. *PLoS One* 2017; **12**(12): e0189621.
15. Zhen X, Lundborg CS, Sun X, Hu X, Dong H. Economic burden of antibiotic resistance in ESKAPE organisms: a systematic review. *Antimicrobial Resistance & Infection Control* 2019; **8**: 1-23.
16. Wozniak TM, Barnsbee L, Lee XJ, Pacella RE. Using the best available data to estimate the cost of antimicrobial resistance: a systematic review. *Antimicrob Resist Infect Control* 2019; **8**: 26.
17. Cassini A, Högberg LD, Plachouras D, et al. Attributable deaths and disability-adjusted life-years caused by infections with antibiotic-resistant bacteria in the EU and the European Economic Area in 2015: a population-level modelling analysis. *The Lancet infectious diseases* 2019; **19**(1): 56-66.
18. Organization WH. Vaccines for antimicrobial resistance (AMR). 2021. https://www.who.int/teams/immunization-vaccines-and-biologicals/product-and-delivery-research/anti-microbial-resistance2023.
19. Economic Analysis and Evaluation Team Department of Health Systems Governance and Financing. WHO-CHOICE estimates of cost for inpatient and outpatient health service delivery. 2021. https://cdn.who.int/media/docs/default-source/health-economics/who-choice-estimates-of-cost-for-inpatient-and-outpatient-health-service-delivery.pdf?sfvrsn=b814d37e_3&download=true2022.
20. Incerti D. Probablistic Sensitivity Analysis in R. https://devinincerti.com/2018/02/10/psa.html#gamma-and-lognormal-distributions (accessed 2022).
21. Schwarzer G. meta: An R package for meta-analysis. *R news* 2007; **7**(3): 40-5.
22. Robinson D, Bryan J, Elias J. Package 'fuzzyjoin'. 2022.
23. World Health Organization. The 2019 WHO AWaRe classification of antibiotics for evaluation and monitoring of use. The 2019 WHO AWaRe classification of antibiotics for evaluation and monitoring of use; 2019.
24. The International Medical Products Price Guide. Medians Spreadsheet. In: Management Sciences for Health Inc, editor.; 2015.
25. Gotham D, Barber MJ, Hill AM. Estimation of cost-based prices for injectable medicines in the WHO Essential Medicines List. *BMJ open* 2019; **9**(9): e027780.
26. Hill AM, Barber MJ, Gotham D. Estimated costs of production and potential prices for the WHO Essential Medicines List. *BMJ global health* 2018; **3**(1): e000571.
27. Torres-Rueda S, Sweeney S, Bozzani F, et al. Stark choices: exploring health sector costs of policy responses to COVID-19 in low-income and middle-income countries. *BMJ global health* 2021; **6**(12): e005759.
28. Villarreal-Fuentes M, Ding S. R tools for ILOSTAT: Rilostat and SMART. *Romanian Statistical Review* 2019; (4).
29. Kim C, Holm M, Frost I, Hasso-Agopsowicz M, Abbas K. Global and regional burden of attributable and associated bacterial antimicrobial resistance avertable by vaccination: modelling study. *BMJ Glob Health* 2023; **8**(7).
30. Bojke L, Soares MO, Claxton K, et al. Reference Case Methods for Expert Elicitation in Health Care Decision Making. *Med Decis Making* 2022; **42**(2): 182-93.
31. Leal J, Wordsworth S, Legood R, Blair E. Eliciting expert opinion for economic models: an applied example. *Value in Health* 2007; **10**(3): 195-203.
32. Antimicrobial Resistance Collaborators. Global burden of bacterial antimicrobial resistance in 2019: a systematic analysis. *Lancet* 2022; **399**(10325): 629-55.

33. Ochalek J, Abbas K, Claxton K, Jit M, Lomas J. Assessing the value of human papillomavirus vaccination in Gavi-eligible low-income and middle-income countries. *BMJ global health* 2020; **5**(10): e003006.
34. Naylor NR. The Antimicrobial Resistance Unit Cost Respository. 2023. https://github.com/NikkiR08/AMR-UCR/tree/main.
35. The World Health Organisation. Life tables by WHO region (GHE: Life tables). 2022.
36. Pike J, Grosse SD. Friction Cost Estimates of Productivity Costs in Cost-of-Illness Studies in Comparison with Human Capital Estimates: A Review. *Appl Health Econ Health Policy* 2018; **16**(6): 765-78.
